# Supplementary material for: Growth and site-specific organization of micron-scale biomolecular devices on living mammalian cells
Source: Nat Commun. 2021 Sep 30;12:5729. doi: 10.1038/s41467-021-25890-z (PMC8484582; doi:10.1038/s41467-021-25890-z)
Supplement: Supplementary file 1 — Supplementary information [file 41467_2021_25890_MOESM1_ESM.pdf]

Supplementary Information

Growth and site-specific organization of micron-scale biomolecular devices on living mammalian cells

Sisi Jia[1], Siew Cheng Phua[2,$], Yuta Nihongaki[2], Yizeng Li[3,4], Michael Pacella[1], Yi Li[1], Abdul M. Mohammed[1], Sean Sun[3], Takanari Inoue[2], Rebecca Schulman[1,5*]

[1] Chemical and Biomolecular Engineering, Johns Hopkins University, Baltimore, Maryland 21218, USA.

[2] Cell Biology, Johns Hopkins University School of Medicine, Baltimore, Maryland 21205, USA.

[3] Mechanical Engineering, Johns Hopkins University, Baltimore, Maryland 21218, USA.

[4] Department of Mechanical Engineering, Kennesaw State University, Marietta, GA 30060

[5] Computer Science, Johns Hopkins University, Baltimore, Maryland 21218, USA.

[$]Current address: Institute of Molecular and Cell Biology, Agency for Science, Technology and Research (A*STAR), 138667, Singapore

* To whom correspondence can be addressed. E-mail: rschulm3@jhu.edu

Table of Contents

Supplementary Figure S1: Synthesis of a PEG-DNA conjugate for coating seeds with PEG………….11
Supplementary Figure S2: Coating DNA nanotube seeds with PEG…………………………………..13
Supplementary Figure S3: Bright field images of HeLa cells cultured in medium supplemented with 3, 6, 9, and 12 mM $MgSO_4$ for two days………………….……………………………….……..14
Supplementary Figure S4: Coating seeds with PEG reduces the extent of nonspecific binding between seeds and the HeLa cell membrane ……………………………………………….……………………19
Supplementary Figure S5: Sample epi-fluorescence micrographs and length distributions of seeded nanotube with 6 nucleotide sticky ends……………………….…………………….……………………23
Supplementary Figure S6: PEG-coated nanotubes do not bind nonspecifically to the HeLa cell surface ………..………………………………………………………………………………….......25
Supplementary Figure S7: Anchoring SpyTag-conjugated, seeded nanotubes to HeLa cells with GFP-integrin-SpyCatcher expressed on their membranes…………………………………………………30
Supplementary Figure S8: Temperature-dependent persistence of EGFR antibody labels on live cells………………………………………………………………………………………………..34
Supplementary Figure S9: Failure to attach secondary antibody-modified seeds to HeLa cell membranes labeled with EGFR antibodies………………….………………….………………..….36
Supplementary Figure S10: HeLa cells after attachment of PEG coated seeds to EGFR receptors via 6 BDC' strands on the seed barrel via AMDA……………….……….………………………………..43
Supplementary Figure S11: The fluorescence intensity of DNA nanotube seeds per HeLa cell after AMDA with EGFR antibodies using different nanotube seed concentrations……………………...….50
Supplementary Figure S12: Average fluorescence intensities of nanotube seeds per HEK293 cell after AMDA or after AMDA omitting BDC tag with 48pM seeded added.……………….……………...54
Supplementary Figure S13: Measurement of forward and side scatter of suspended HEK293 cells with nanotube seeds attached using EGFR AMDA during flow cytometry……………….……………...56
Supplementary Figure S14: The average fluorescence intensities of HeLa cells with seeded nanotubes or seeds attached via AMDA and AMDA with BDC tag addition omitted (Supplementary Note S28)…………………………………………………………………………………………..65
Supplementary Figure S15: Attachment of nanotube seeds and seeded nanotubes to integrin receptors on HeLa cells using integrin AMDA……………….….……………………………………………68
Supplementary Figure S16: Maximum projection images of HeLa cells at different times after seeded nanotube attachment using EGFR AMDA. …...…………………………………………………….70
Supplementary Figure S17: (a) Diagram of a DNA nanotube in a rectangular chamber. (b) Diagram of DNA nanotube deflection under flow Q. (c) The rotation of a DNA nanotube about its anchor from the top view…….………………………………………………………………………………72
Supplementary Figure S18: Shear stress on the lower surface of the chamber as a function of Q……………………………………………………………………………………..….…73
Supplementary Figure S19: Rotational angles θ and ϕ (in radians) as functions of Q for different G_θ and G_ϕ………………………………………………………………………………………………77
Supplementary Figure S20: Rotational angles θ and ϕ (in radians) as functions of ℓ for different G_θ and G_ϕ………………………………………………………………………………………………77
Supplementary Figure S21: Histograms of ϕ for different Q. Here $G_\theta = G_\phi = 1$ nN·nm………...…….78
Supplementary Figure S22: Model of the distribution of θ and ϕ and the MSD of θ and ϕ for different volume flow rates……………….……………………………………………………………79
Supplementary Figure S23: Total force on a DNA nanotube as a function of DNA nanotube length for different polar angles and flow rates……………….……………….………………...……………...80
Supplementary Figure S24: Model prediction (a simplified model) of the distribution of ϕ and the MSD of ϕ for different volume flow rates……………….…….……………………….……………82
Supplementary Figure S25: Measurement of the total angle of nanotube rotation Φ………..…..…90
Supplementary Figure S26: Measuring the rate of end-to-end joining of DNA nanotubes in solution………………………………………………………………………………………94
Supplementary Figure S27: Images of 4PEG nanotubes grow from seeds of type A (a) and 4PEG seeded nanotubes grown from seeds of type B (b) ……………………...…………………………..98
Supplementary Figure S28: Schematic of end-to-end joining of seeded nanotubes to nanotubes anchored to the cell membrane……………….……………………………………………………...99

Supplementary Figure S29: End-to-end joining of DNA nanotubes anchored to a glass surface to nanotubes in solution…………………………………………………………………………………...104
Supplementary Figure S30: Joined nanotubes can fracture in flow………………………………..106
Supplementary Figure S31: Length distributions of seeded nanotubes after end-to-end joining. ……107
Supplementary Figure S32: The rate of thermal motion of seeded nanotubes anchored to a glass surface is reduced in 0.6% methylcellulose medium……………………………………………………109
Supplementary Figure S33: 3-dimensional reconstructed images of HeLa cells with anchored nanotubes extended via end-to-end joining in 0.6% methylcellulose……………………………..……..111
Supplementary Figure S34: The architecture of the Cy3 labeled 6nt nanotube monomer (the SEs tile) with no PEG (a) and with PEG modification (b) ………………………………………………….112
Supplementary Figure S35: The architecture of the atto647 labeled 6nt nanotube monomer……………………………………………………………………………………113
Supplementary Figure S36: The architectures of Cy3 labeled 4nt monomers: SEd and REd tile with and without PEG…………………..……………………………………………………………….114
Supplementary Figure S37: Schematics showing the architectures of the 4nt nanotube monomers labeled with atto488 (a) and atto647 (b) by labeling the central strands (REd_3 and SEd_3) with atto488 or atto647……..……………………………………………………………………115
Supplementary Figure S38: The 4nt inactive REd monomer was activated by adding the “activation strand” ………………………………………………………………………………………...117
Supplementary Figure S39: Schematic for DNA nanotube seeds showing the positions of adapters, PEG modification sites, fluorescent labeling sites, and biotin attachment sites..…..…………………118
Supplementary Figure S40. Map showing the positions of the staple strands, PEG-DNA conjugate binding sites, and fluorescence labeling strand sites and fluorescence labeling strands on the seed………………………………………………………………………………..…………..119
Supplementary Figure S41: Structure of the 15 nucleotide DNA overhang on a staple that serves as an attachment site for a PEG-DNA conjugate……………………………………………………119
Supplementary Figure S42: Adapter organization on nanotube seeds…………………...…………125
Supplementary Figure S43: Structures of the assembled seed A adapters for 6 nt nanotubes……...…126
Supplementary Figure S44: Structures of the assembled seed B adapters for 6 nt nanotubes………..127
Supplementary Figure S45: Structures of the assembled seed A adapters for 4 nt nanotubes……..…129
Supplementary Figure S46: Structures of the assembled seed B adapters for 4 nt nanotubes…..……130
Supplementary Figure S47: Schematic showing the positions of the BDC strand attachment sites on the PEG coated seeds……………………………………………………………………………132
Supplementary Figure S48: Structure of the BDC tag attachment sites at the left ends of nanotube seeds A (a) and right ends of nanotube seed B (b) ……...………………………………………….132
Supplementary Figure S49: Structure of the extended BDC tag attachment sites at the right ends of nanotube seeds…………………………………………………………………………….133
Supplementary Figure S50: Structure of the amino attachment sites at the left ends of nanotube seeds……………………………………………………………………………………...…135

Section 1. Nonspecific attachment of nanotube seeds/seeded nanotube to cells

Supplementary Note S1: Prepare the DNA nanotube seeds without Poly(ethylene) glycol (PEG) coating. ……………………………………...…………………………………………………….7
Supplementary Note S2: Preparation of PEG-DNA conjugates………………………………………..9
2.1 Synthesis of PEG-DNA conjugates……………………………………………………………9
2.2 Purification of the PEG-DNA conjugate……………………………………………………10
2.3 Measurement of the concentration of PEG-DNA conjugate in stock solutions……………........11
Supplementary Note S3: Coating nanotube seeds with PEG…………………………………..……12
Supplementary Note S4: Culturing HeLa cells in a medium with high Mg2+ concentrations………...14
Supplementary Note S5: Characterization of the extent of nonspecific binding between DNA nanotube seeds and live HeLa cells……………………………………………………...………….…15
Supplementary Note S6: Quantifying the average amount of nanotube seeds attached nonspecifically per HeLa cell…………………...…………………………………………………………………16
6.1 Measurement of the average fluorescent intensity of seeds per cell ………………..……..….16
6.2 Measurement of the average number of seeds per cell……………………………………….17
Supplementary Note S7: Protocol for growing 6nt seeded nanotubes…………………………..……..20

7.1 Assembly of DNA nanotubes without PEG coating……………………….……………………20
7.2 Assembly of DNA nanotubes with PEG coating……………………………..………………21
7.3 Protocols for measuring the lengths of nanotubes………………..…………………………...22
Supplementary Note S8: Characterizing the extent of nonspecific binding between seeded DNA nanotubes and live HeLa cells……………………………………….……………………...….24
Section 2: SpyTag-SpyCatcher chemistry
Supplementary Note S9: Conjugation of the SpyTag peptide to a DNA strand……………….…….26
Supplementary Note S10: Transfection of HeLa cells with GFP-integrin-SpyCatcher, GFP-integrin plasmids………………………………………………………………………………….…….27
Supplementary Note S11: Attachment of SpyTag-modified DNA seeded nanotubes to HeLa cells expressing the GFP-integrin-SpyCatcher transgene……………………………….………..….28
Supplementary Note S12: Estimation of the binding flux of DNA nanotubes on the cell surface using SpyCatcher-SpyTag binding………………………………………………………………...….31
Section 3. Antibody-mediated DNA nanotube anchoring (AMDA) method
Supplementary Note S13: Staining fixed HeLa cells with EGFR antibodies at room temperature……32
Supplementary Note S14: Staining live HeLa cells with EGFR antibodies at room temperature...…...33
Supplementary Note S15: Staining live cells with EGFR antibodies at 4ºC………………..…...…..33
Supplementary Note S16: Attachment of secondary antibody-labeled DNA nanotube seeds to EGFR receptors labeled with primary antibodies on the HeLa cell membrane…………………………..…35
Supplementary Note S17: The binding affinity of the BDC sequence to its complement...……..…….37
Supplementary Note S18: Summary of reagents used for antibody-mediated DNA nanotube anchoring (AMDA) and their incubation processes…………………………………………………….……38
Supplementary Note S19: Attachment of nanotube seeds with 6 BDC' strands on the seed barrel to HeLa cells using EGFR AMDA……………………………………………...…………...…40
Supplementary Note S20: Attachment of nanotube seeds with 6 BDC' strands on the seed barrel and 30 BDC' strands on the scaffold loop to HeLa cells using EGFR AMDA……………………….……44
Supplementary Note S21: Stain live HeLa cells with DiD dye…………………………..…………48
Supplementary Note S22: Measuring the number of seeds attached to an average HeLa cell after EGFR AMDA………………………………………………………………………………………49
Supplementary Note S23: Attachment of nanotube seeds with 6 BDC' strands on the seed barrel and 30 BDC' strands on the scaffold loop to suspended HEK293 cells……………………………………..51
Supplementary Note S24: Quantification of the number of nanotube seeds attached to suspended HEK293 cells by EGFR AMDA using flow cytometry……………………………..……………53
24.1 Measurement of the average fluorescent intensity of seeds per cell using confocal images……………..……..……..……..……..……..……..……..……..……..……..……..53
24.2 Measurement of the average fluorescent intensity of seeds per cell by flow cytometry……….54
Supplementary Note S25: Measuring the correlation in locations of nanotube seeds and antibodies on cell membranes using confocal micrographs…………………………………………………..…57
Supplementary Note S26: Attachment of seeded nanotubes to EGFR receptors on HeLa cells using the EGFR AMDA protocol……………………………………..……………………………….58
Supplementary Note S27: Quantifying the number of seeded nanotubes attached to the HeLa cell membranes……………………………………………………………………………………..…61
Supplementary Note S28: Attachment of seeded nanotubes to EGFR receptors on suspended HEK293 cells using EGFR AMDA…………………………………………………………………….....63
Supplementary Note S29: Quantifying the number of seeded nanotubes attached to suspended HEK293 cells…..……………………………………………..……………………………….……63
29.1 Measurement of the average fluorescent intensity of seeded nanotube on a cell ……………..63
29.2 Measurement of the average number of seeded nanotubes per cell ………………….……..65
Supplementary Note S30: Attachment of nanotube seeds and seeded nanotubes to integrin receptors on HeLa cells using integrin AMDA…………………………………………………………………66
Supplementary Note S31: Measurement of the rate of seed import into or detachment from HeLa cells…..…..………………………………………………………………………………………69
Supplementary Note S32: Measurement of the rate of seeded nanotube import into or detachment from HeLa cells……………………………………………………………………………………...…70
Supplementary Note S33: Measuring the persistence time of seeds and seeded nanotubes on the HeLa cell membrane after EGFR AMDA……………………………………………………………………71
Section 4: Nanotubes as flow meters on the live cell membrane
Supplementary Note S34: Simulation of nanotube position in a flow field……………………………72

34.1 The flow in a fluid cell chamber……………………………….…………………………72
34.2 A full model of the bending of a DNA nanotube in a flow field……………………….……74
34.3 A simplified model……………..………………………………………………………….81
Supplementary Note S35: Determination of the shear stresses induced by different laminar flow rates in different flow cells……………………………………………………………………………...83
Supplementary Note S36: Protocols for treating glass bottom channels to anchor seeded nanotube on their surfaces……………………….……………………………………………………………...84
Supplementary Note S37: Protocol for attaching seeded nanotubes to the glass bottom of a μ-slide channel…………………………………...…………………………………………………..85
Supplementary Note S38: Characterizing the responses of seeded nanotubes anchored on a glass surface to differing amounts of shear stress induced by laminar fluid flow……………………………86
Supplementary Note S39: Protocol for attaching seeded nanotubes to EGFR receptors on HeLa cells using the EGFR AMDA in μ-slide channels……………………………………………...……87
Supplementary Note S40: Characterizing the responses of seeded nanotubes anchored on HeLa cell membrane to shear stress induced by laminar fluid flow……………………………………………89
Supplementary Note S41: Measuring the total angles of rotation of nanotubes under different amounts of shear stress induced by laminar fluid flow……………………………………………..……90
Section 5. Growing nanotubes on living cells by nanotube end-to-end joining
Supplementary Note S42: 6nt seeded nanotube end-to-end joining in solution………………………91
Supplementary Note S43: Assembly of 4PEG nanotubes for nanotube joining…………………...….95
43.1. Assembly of 4PEG seeds and capping seeds…………………………………….…...…….95
43.2. Protocol for growing 4PEG nanotubes……………………………..………………………97
Supplementary Note S44: Assembly of inactive monomers used as nano-glue…………………..……99
Supplementary Note S45: Measuring the joining rate between 4PEG nanotubes anchored to a glass surface and 4PEG nanotubes in solution in the presence of additional monomers (nanoglue) ………101
Supplementary Note S46: Joining of nanotubes anchored to the HeLa cell surface…………………..105
Supplementary Note S47: Visualizing nanotubes on the cell surface by adding methylcellulose to reduce thermal motion………………………………..……………………………………………108
Supplementary Note S48: Visualizing joined nanotubes on the cell surface by adding methylcellulose to reduce the rates of nanotubes' thermal motion……………………………………..……………110
Section 6. DNA seeded nanotube structure and sequence
Supplementary Note S49: Nanotube monomer structures and sequences ……………………………112
49.1 Nanotube monomers with 6 nucleotide sticky end overhangs (6nt monomers) with and without polyethylene glycol (PEG) modification……………………………………………………..112
49.2 4nt monomers with and without PEG modification…………………….…………………114
49.3 4nt inactive monomer design………………………………………….……………………117
Supplementary Note S50: Design and sequences of DNA nanotube seeds…………………………..118
50.1 DNA nanotube seeds staple design for coating with PEG……………………………………119
50.2 Strands for fluorescently labeling DNA nanotube seeds……………………………………...122
50.3 Adapter design and sequences………………………..…………………….…………………125
50.4 Biotin attachment strand design for AMDA…………………………..…………………..…132
50.5 Amino attachment linker strands for SpyTag……………………….…………………...135
Supplementary Note S51: Sequence of the the plasmid DNA Integrin-SpyCatcher-GFP……..……..136

Supplementary Table S1: 6nt nanotube seeds with 6 biotin modifications………………….……….…7
Supplementary Table S2: Recipe for synthesis of the PEG-DNA conjugates…………………………..9
Supplementary Table S3: Reagents used to coat nanotube seeds with PEG……………….……..…12
Supplementary Table S4: Recipe for 6nt monomer (Cy3 labeled) without PEG………………………20
Supplementary Table S5: Recipe for 6nt monomers (Cy3 labeled) with PEG……………...…………21
Supplementary Table S6: Reagents for the synthesis of the DNA strand-DIBAC conjugate………..…26
Supplementary Table S7: 6nt nanotube seeds with SpyTag modification……………….……………..28
Supplementary Table S8: 6nt nanotube seeds without SpyTag modification…………………………..29
Supplementary Table S9: Reagents for modifying nanotube seeds with secondary antibodies..…...…35
Supplementary Table S10: Reagents used in AMDA and how cells were incubated with each reagent…………………………………………………………………………………………..38
Supplementary Table S11: 6nt nanotube seeds with 6 BDC' tag attachment sites…………..…….....40
Supplementary Table S12: 6nt nanotube seed B with 36 BDC tag attachment sites (atto488

labeled) …………………………………………………………………………………………………44
Supplementary Table S13: 6nt nanotube seeds with no BDC tag attachment sites…………………….45
Supplementary Table S16: 6nt nanotube seed B with 36 BDC tag attachment sites (atto647 labeled) ……………………………………………………………………………..……..…….58
Supplementary Table S15: Default parameters in the full model…………..…………………….…..80
Supplementary Table S16: Default parameters in the simplified model………..…………………...82
Supplementary Table S17: Fluid flow shear stresses and flow rates for nanotubes on cell membranes and glass surfaces………..………..……….……………..………..…………………......83
Supplementary Table S18: 6nt nanotube seed A with 36 BDC tag attachment sites (atto488 labeled) …………………………………………………………………………………………91
Supplementary Table S19: Recipe for 6nt monomer (atto647 labeled) without PEG…………………92
Supplementary Table S20: Recipe for unlabeled seeds of type A for 4PEG nanotubes with 36 biotin attachment sites………..………..………..………..………..……………………………..95
Supplementary Table S21: Recipe for Atto647 labeled seeds of type A for 4PEG nanotubes with 36 biotin attachment sites………..………...………..………..…..…………………….……..96
Supplementary Table S22: Recipe for unlabeled seeds of type B for 4PEG nanotubes with no biotin attachment sites………..………..………..……….……………………………..……....96
Supplementary Table S23: Recipe for 4nt monomers with PEG………..……………………...…97
Supplementary Table S24: Recipe for Atto488 labeled inactive monomers……………...……..…100
Supplementary Table S25: Recipe for Atto647 labeled inactive monomers…..………..…………....100
Supplementary Table S26: Mixture of capped seeded nanotubes and nanotube monomers for end-to-end joining……………………………………………………………………………..……..101
Supplementary Table S27: Sequences of 6nt SEs nanotube monomer for SEs tiles.………..…..…….113
Supplementary Table S28: Sequences for the 4nt nanotube monomer for SEd and REd tiles.……….116
Supplementary Table S29: Sequences for inactive 4nt REd monomer and the activation strand…….117
Supplementary Table S30: Stable sequences of the DNA nanotube seeds with 15 nucleotide DNA overhang for PEG-DNA attachment and sequence for the amino-modified DNA strand used to produce the PEG-DNA conjugate that binds to seeds. ………………………………………..………120
Supplementary Table S31: Sequences of the fluorescent labeling strands and the fluorescence attachment strands on M13 ………………………………………………………………...122
Supplementary Table S32: Sequences of 6nt seed A adapters and 6nt seed B adapters. ……………..127
Supplementary Table S33: Sequences of 4nt seed A adapters and 4nt seed B adapters. ……………..130
Supplementary Table S34: Sequences of the biotin attachment strands which included the biotin left-side attachment strands for A seeds, the biotin right-side attachment strands for B seeds, the extended biotin attachment strand sequences for B seeds and the additiona l 30 biotin attachment strands on extra M13. ……………………………………………………………………………………..133
Supplementary Table S35: Sequences of the amino attachment strands and amino modified DNA for conjugating with SpyTag…….…………………………………………………………………..135

Section 1. Nonspecific attachment of nanotube seeds/seeded nanotube to cells

Supplementary Note S1: Preparation of DNA nanotube seeds without Poly(ethylene) glycol (PEG) coating.

To assemble each type of DNA nanotube seed used in this work, a 100 µl mixture (recipes delineated for each type in each protocols below) was made containing M13mp18 scaffold, staple strands, adapter strands, fluorescent strands, fluorescent attachment strands, and amine or biotin attachment strands in TAE-Mg^{2+} buffer (40mM Tris-Acetate, 1mM EDTA and 12.5mM magnesium acetate). A variety of attachment strategies were tested for attaching nanotube seeds or seeded nanotubes to the cell membrane, each of which necessitated a different design for the seeds. Here we describe the process of preparing the seeds for the study of nonspecific attachment of seeds to cell as an example.

(1) Prepared the seeds strands mixtures:

6nt DNA nanotube seeds labeled with atto488 were assembled by first mixing the components listed in Supplementary Table S1 below. The design for this seed was adopted from Mohammed *et al*[1]. It has 6 BDC tags at the right end of the seed's barrel (as illustrated) and labeled with atto488. Nanotubes grow from the left sides (as illustrated) of these seeds.

Supplementary Table S1: 6nt nanotube seeds with 6 biotin modifications

Seed Assembly Mixture	Final desired concentration (nM or fold)	Stock concentration (nM or fold)	To add (µl)
H_2O	--	--	71.4 µl
TAE-Mg^{2+} buffer	1x	10x	10 µl
Seed staple strands mix	250 nM	4167 nM	6 µl
Seed B adapter strands mix (6nt)	100 nM	4167 nM	2.4 µl
M13mp18 scaffold strand	5 nM	100 nM	5 µl
biotin right attachment strands mix	20 nM	1000 nM	2 µl
BDC tag	120 nM	10000 nM	1.2 µl
96Seed labeling attachment strands mix	10 nM	1000 nM	1 µl
Labeling strand ATTO488	1000 nM	100000 nM	1 µl
Total			100 µl

Seed staple strands mix: A mixture containing the 72 seed staple strands (Supplementary Note S50.1, Supplementary Figure S40) in water each at concentration 4167nM (300 µM/72).

Seed B adapter strand mix (6nt): A mixture containing all 24 Seed B adapter strands for 6nt nanotubes (Supplementary Note S50.3 and Supplementary Figure S44) in water each at 4176nM (100µM/24).

Biotin right attachment strands mix: A mixture containing all six biotin attachment

linker strands for seed B (BDC' strand_rightside_01 to BDC' strand_rightside_06) in water each at 1000nM (see Supplementary Note S50.4 and Supplementary Figure S48b).

BDC tag: The strand *BDC tag* in water at 10000nM (Supplementary Note S50.4).

96 Seed labeling attachment strands mix: A mixture containing all 96 labeling attachment strands (Supplementary Note S50.2 and Supplementary Figure S40); Unused_m13mp18_01_OLS to Unused_m13mp18_96_OLS) in water each at a concentration of 1000nM.

Labeling strand ATTO488: The strand *Labeling strand ATTO488* in water at 100000nM (Supplementary Note S50.2)

(2) 6 nucleotide sticky end-nanotube seed annealing protocol

The seeds strands mixture was annealed in a thermocycler (Eppendorf Mastercycler) using the following annealing schedule, as described in Yi *et al* [2]:

- 5 mins at 90°C
- 90°C to 45°C at 1°C/min
- 45°C for 60 mins
- 45°C to 37°C at 1°C/10mins
- 37°C hold until sample retrieval

(3) Purification of seeds and measurement of seed concentrations in stock solution

To remove the excess staple, adapter, biotin attachment, fluorescent attachment, and labeling strands, the solution of nanotube seeds was purified using the method adopted from *Agrawal et al* [3] by using a 100kDa Amicon ultra-0.5mL centrifugal filter (UFC510096, MilliporeSigma).

100 µl of seed solution and 300 µL TAE-Mg^{2+} buffer was added to the filter and centrifuged at 3000xg for 4 minutes in a fixed-angle centrifuge to remove excess DNA strands. Then the sample was washed 4 times by adding 400 µl of TAE-Mg^{2+} buffer to the remaining solution and repeating centrifugation. The remaining solution was recovered by spinning the inverted filter in a fresh tube, after which the purified mixtures was stored at 4 °C until use.

The concentration of seeds in the stock solution was measured after purification using the method developed in *Agrawal et al.*[3] After purification, 0.3 µL of the solution containing seeds was added to a 19.7 µL monomer mix solution (Supplementary Table S4). 6 µL of this diluted seed solution was pipetted onto an 18x18 cm cover slip (48366045, VWR, 0.13-0.16mm) to image under a fluorescence microscope. Dilution of the purified seeds continued until there were 100-200 seeds per field of view (87µm x 87µm). This number of seeds in this size field of view corresponds to a concentration of about 6 pM. The concentration of seeds in the stock solution was calculated from the fold-dilution needed to achieve this density of seeds during imaging.

Supplementary Note S2: Preparation of PEG-DNA conjugates

2.1 Synthesis of PEG-DNA conjugates

The N-hydroxylsuccinimide (NHS) functionalized polyethylene glycol valeric acid (NHS-PEG20k) was covalently conjugated to amino modified DNA strands (Amino_DNA) through the reaction between NHS-ester and primary amine which yield stable amide bond (Supplementary Figure S1a). The recipe used is shown as Supplementary Table S2.

To reduce nonspecific interactions between seeded DNA nanotubes and the cell membrane, both the seeds and nanotubes were modified with PEG which required conjugating PEG to corresponding DNA strands. To coat the nanotube seeds with PEG, the amino modified DNA strand Seed PEG-attachment strand (Supplementary Note S50.1 and Supplementary Figure S40, S41) was conjugated with PEG using the recipe in Supplementary Table S2, where the AminoDNA is the Seed PEG-attachment strand. The resulting PEG-DNA conjugate could hybridize to all the 72 binding sites extended from the seeds forming a PEG coating for the nanotube seeds. To coat the 6nt nanotubes with PEG, the SEs tile monomer was modified with PEG by conjugating the amino modified central strand, SEs_3-5'Cy3-3'amine sequence (Supplementary Note S49.1 and Supplementary Figure S34b), with PEG using the recipe in Supplementary Table S2, where the Amino DNA is SEs_3-5'Cy3-3'amine. Similarly, to coat the 4nt nanotubes with PEG, both REd and SEd tile monomers were modified with PEG by conjugating the amino modified central strands, REd_3-5'Cy3-3'amine and SEd_3-5'Cy3-3'amine (Supplementary Note S49.2 and Supplementary Figure S36b), with PEG using two mixtures following this recipe where in the first the Amino DNA is 5'Cy3-3'amine and in the second the Amino DNA is SEd_3-5'Cy3-3'amine

Supplementary Table S2: Recipe for synthesis of the PEG-DNA conjugates

	Final desired concentration (µM or fold)	Stock concentration (nM or fold)	To add (µl)
H_2O			40 µl
Amino DNA	50 µM	100 µM	50 µl
NHS-PEG20k	4000 µM		8mg
PBS buffer (pH 7.2)	1x	10x	10 µl
Total			100 µl

Amino DNA: For 6nt nanotubes, the SEs_3-5'Cy3-3'amine sequence (Supplementary Note S49.1 and Supplementary Figure S34b) was used to conjugate with PEG by this recipe for assembling PEG modified 6nt nanotube monomers. For 4nt nanotubes, the REd_3-5'Cy3-3'amine and SEd_3-5'Cy3-3'amine (Supplementary Note S49.2 and Supplementary Figure S36b) were conjugated with PEG individually by this recipe. For nanotube seeds, the Seed PEG-attachment strand (Supplementary

Note S50.1 and Supplementary Figure S41) was used to prepare PEG-DNA conjugates to coat nanotube seeds with PEG.

NHS-PEG20k: Succinimidyl valeric acid PEG, mPEG-SVA (PG1-SVA-20k, NANOCS). The N-hydroxylsuccinimide (NHS) functionalized on polyethylene glycol valeric acid (NHS-PEG20k) will react with the amino (-NH2) group on the amino-modified DNA strand.

PBS buffer (28372, ThermoFisher) was prepared to 10x.

The Amino_DNA, H_2O and the PBS buffer were first combined in amounts and at concentrations as listed in the recipe above. 8mg of NHS-PEG20k was then added and the mixture was agitated at room temperature (19-20°C) overnight to allow the reaction (Supplementary Figure S1a) to complete.

2.2 Purification of the PEG-DNA conjugate

(1) After incubation overnight, the mixture was loaded into a 7% PAGE gel (running buffer TAE-Mg^{2+}, loading buffer 1x gel loading dye blue). The gel was run at 150V for 1h. During purification of the seed PEG-attachment strand-PEG conjugate, the gel was stained with Sybr gold for 30 minutes to allow for visualization under gel imager (Supplementary Figure S1b). The SEs_3-5'Cy3-3'amine-PEG conjugate strand has a Cy3 label, so did not need to be stained with DNA dye to see the desired band.

(2) The conjugate migrates much more slowly in the gel than the unconjugated amino DNA strand, so could be easily identified. It was cut from the gel and chopped into as small of pieces as possible. The pieces were then transferred into a 1.5 mL Eppendorf tube and MilliQ water was added to immerse the gel. The sample was then shaken for 2-4 days to allow the PEG-DNA conjugate to diffuse into the water.

(3) The sample was centrifuged at 6000xg for 5 minutes. All of the solution was then carefully pipetted into a new 1.5mL Eppendorf tube. The PEG-DNA conjugate and some very small pieces of gel were in the solution. To get rid of these small pieces of gel, the sample was centrifuged again at 6000xg for 5minutes and the gel pieces all moved to the bottom of the tube. The supernatant was carefully pipetted into a new 1.5mL tube without disturbing the gel pieces at the bottom. This supernatant contained the purified PEG-DNA conjugate.

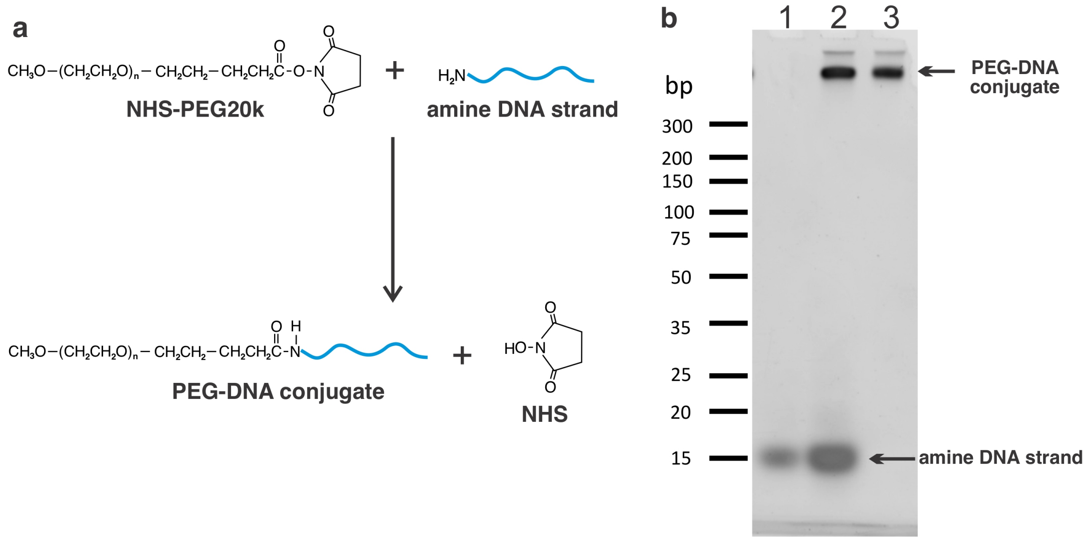

Supplementary Figure S1: Synthesis of a PEG-DNA conjugate for coating seeds with PEG. a. Schematic of the conjugation reaction. **b.** PAGE gel electrophoresis of the "Seed PEG-attachment strand" (Supplementary Note 50.1) after PEG-DNA conjugation. L1: amino DNA strand, L2: the reaction mixture after conjugation of PEG and amino DNA before gel purification, L3: the conjugate after purification. This experiment was repeated more than three times independently with similar results.

2.3 Measurement of the concentration of PEG-DNA conjugate in stock solutions

The concentration of Cy3-labeled PEG-DNA conjugates was measured using fluorescence absorbance as described in Li *et al*[2]. The concentration of unlabeled PEG-DNA conjugates was measured by running a PAGE gel with a series of amine-DNA concentrations (from 0.5-4 μM) to determine the band intensity of a particular strand of DNA along with the PEG-DNA conjugate. The concentration of the PEG-DNA conjugate was determined by comparing its intensity in the gel to those of the calibration bands.

Supplementary Note S3: Coating nanotube seeds with PEG

PEG-coated seeds were prepared by first folding the origami seeds as described in Supplementary Note S1 and then combining them with PEG-DNA conjugates that hybridized to tags on their exterior as shown in Supplementary Figure S2a. We first assembled the nanotube seeds with the 72 binding sites for the PEG-DNA conjugate[2] as described in Supplementary Note S1. For the 6nt unmodified seeds with the desired adapter strands, BDC' tag and labeling were assembled by combining the reagents as listed in the recipes described in Supplementary Table S1 in Supplementary Note S1 step 1, then annealing as described in Supplementary Note S1 step 2, purifying the seeds and determine the seeds concentrations as in Supplementary Note S1 step 3.

In the second step, proper amount of the PEG-DNA conjugate was added to the unmodified seed solution prepared in the first step to form the PEG coated nanotube seeds. The PEG-DNA conjugate for attachment to nanotube seeds was synthesized as described in Supplementary Note S3. They will bind to the 72 binding sites on the seeds by DNA hybridization to coat the whole nanotube seeds with PEG. To determine the concentration of the PEG-DNA conjugate that should be added to seeds to reliably attach to all binding sites for the DNA sequence, we assumed that the purified seed concentration is n Molar. The seeds present at 72 binding sites for the conjugates (Supplementary Figure S40), so the concentration of PEG-DNA should be at least $72n$ Molar. To ensure the seeds were coated with the conjugate, we used a 30-fold excess of PEG-DNA over the number of binding sites: $72n*30=2160n$. To coat 100 μL of purified seed solution at 0.8nM with PEG, the recipe was shown in Supplementary Table S3.

Supplementary Table S3: Reagents used to coat nanotube seeds with PEG

	Final desired concentration	Stock concentration	Volume
PEG-DNA conjugate	1.8 μM	10 μM	18 μL
TAE-Mg^{2+} buffer	1x	10x	1.8 μL
Purified seeds	0.67 nM	0.8nM	100 μL

1.8 μL 10x TAE-Mg^{2+} buffer was added to 18 μL of the PEG-DNA solution. 100 μL of the purified seed solution (prepared as described in Supplementary Note S1) was then added to this mixture. The mixture was then incubated at room temperature for 30min. After 30min, the seeds were directly used to grow seeded nanotubes without further purification.

To verify that the PEG-DNA conjugates attached to the seeds, the resulting structures and unmodified seeds were run in a 1% agarose gel (1xTAE-Mg^{2+} buffer), 100V for 1.5 hours. The gel was stained with Sybr gold for 30 minutes. The seeds coated with PEG ran much more slowly than both of the seeds without a PEG coating and the seeds only coated with the amino DNA strand (Supplementary Figure S2b).

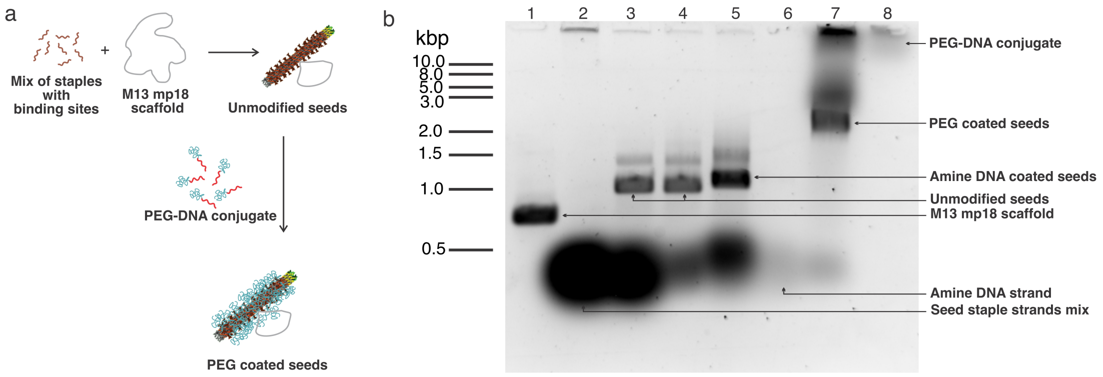

Supplementary Figure S2: Coating DNA nanotube seeds with PEG. a. Scheme for attaching DNA-PEG conjugates to nanotube seeds *via* DNA hybridization. **b.** Results of agarose gel electrophoresis comparing the mobility of seeds with and without PEG coating. Lane 1: M13mp18, Lane 2: Seed staple strands mix (Supplementary Note S1), Lane 3: nanotube seeds before purification (Supplementary Note S1 steps 1 and 2), Line 4: purified nanotube seeds (Supplementary Note S1 step 3), Line 5: purified nanotube seeds coated with amine DNA strand *Seed PEG-attachment strand* (Supplementary Note S50.1 and Supplementary Figure S41), Line 6: amine DNA strand *Seed PEG-attachment strand*, Line 7, purified nanotube seeds with PEG coating, Lane 8: PEG-DNA conjugate. This experiment was repeated more than three times independently with similar results.

Supplementary Note S4: Culturing HeLa cells in a medium with high Mg^{2+} concentrations.

DNA nanostructures have generally been prepared in media containing divalent cations, in particular Mg^{2+}. These divalent cations are thought to stabilize multi-helical structures by acting as salt bridges, and either significant Mg^{2+} (3-20 mM) or very high Na^{+} (1 M) concentrations are required to stabilize many DNA origami structures.[4,5] In order to attach DNA nanostructures to cells, we therefore sought to culture cells under conditions where DNA nanostructures would be stable. We chose to do this by altering the concentrations of Mg^{2+} in the cell growth medium by adding[5] different concentrations of $MgSO_4$ to DMEM medium containing 10% FBS and 1% penicillin-streptomycin. HeLa cells were cultured in media with 3, 6, 9, and 12 mM $MgSO_4$ overnight and imaged under a microscope the next morning to check cell viability by characterizing cell shape. Cells were viable in cell media containing all of the tested concentrations of $MgSO_4$ (Supplementary Figure S3).

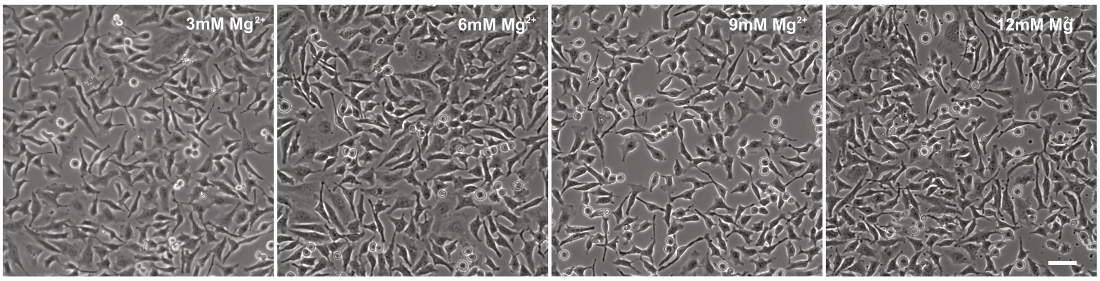

Supplementary Figure S3: Bright field images of HeLa cells cultured in medium supplemented with 3, 6, 9, and 12 mM $MgSO_4$ for two days. Scale bar 100μm. This experiment was repeated more than three times independently with similar results.

Supplementary Note S5: Characterization of the extent of nonspecific binding between DNA nanotube seeds and live HeLa cells

In this experiment, different concentrations of otherwise unmodified DNA nanotube seeds with and without PEG coating were directly added to the HeLa cells.

(1) Preparation of nanotube seeds with and without PEG coating:

6nt DNA nanotube seeds labeled with atto488 were assembled by mixing the components listed in Supplementary Table S1 as in Supplementary Note S1 step 1, then annealed as described in Supplementary Note S1 step 2, and purified as in Supplementary Note S1 step 3. This seed solution was aliquoted: one aliquot was used as a solution of seeds without PEG coating and the other aliquot was used to prepare PEG-coated seeds as described in Supplementary Note S3. The concentrations of the seeds with and without PEG coating were determined as described in Supplementary Note S1 step 3.

(2) HeLa cell preparation:

The HeLa cells were cultured and passaged as described in the online methods. The cell concentration was determined by counting with a hemacytometer. Then the cells were diluted to 1.6×10^5 cell per mL in cell growth medium (DMEM medium containing 10% FBS and 1% penicillin-streptomycin). For each well of the Lab-Tek 8-well chambers (155411PK, ThermoFisher, referred to as 8-well chamber), 250 μL of diluted cell were added, *i.e.* 4×10^4 cell per well. Shaking of the cells was avoided to prevent the cells from becoming inhomogeneously distributed. The chamber was carefully transferred back to cell culture incubator. The cells were kept in the incubator overnight and for use the next morning.

The next morning, the covered-glass chambers with the cells were moved into the refrigerator (4°C) and incubated for 10 minutes. All the buffers (DMEM-12mM $MgSO_4$, 1% BSA (DMEM) buffer and 1% BSA (DMEM)-12mM $MgSO_4$ buffer) were kept on ice during this process.

(3) Incubation of cells with 1% BSA solution:

To reduce nonspecific binding of antibodies to cells, the cells in each well were first incubated with 1% BSA solution. The medium in each well containing cells was exchanged to cold 250 μL 1% BSA (DMEM) buffer. When exchanging buffer in the wells with cell, caution was used to always keep the pipette tip against the corner of the well when removing or adding solution as pipetting buffer directly onto the cells could cause cell death. The cells were then incubated in the refrigerator (4°C) for 5 minutes.

(4) Nanotube seeds incubation with HeLa cells:

Cold 1% BSA(DMEM)-12mM $MgSO_4$ buffer was used to dilute the seeds with and without PEG coating to make individual 250 μL solutions containing 8 pM, 16 pM, 32 pM or 64 pM of seeds. The 1% BSA(DMEM) in each well of cells was exchanged for one of the diluted seed solutions. The samples were then incubated in the

refrigerator (4°C) for 30 minutes.

(5) Washing of cells to remove unattached nanotube seeds:

After 30 minutes, each well was washed with cold DMEM-12mM $MgSO_4$ buffer 3 times to remove the seeds not attached to the cells. During each wash cycle, the solution in the well was carefully removed and 250 µL fresh cold DMEM-12mM $MgSO_4$ buffer was immediately added with a pipette. The cells were kept on ice and gently shaken for 5 minutes after the second and third wash cycles.

(6) Fixing cell for imaging:

The cells were fixed with 4% paraformaldehyde by exchanging the buffer in each well with 250uL 4% paraformaldehyde (in PBS) with 12mM $MgSO_4$. The samples were then incubated on a bench (19-21°C) for 10 minutes in the dark (covered with foil). After 10 minutes, the cells were washed with PBS buffer containing 12mM $MgSO_4$ 3 times. During each wash cycle, the solution in the well was carefully removed and 250 µL fresh PBS buffer with 12mM $MgSO_4$ was immediately added.

(7) Cell imaging under a spinning disk confocal microscope:

The cells were imaged using a Zeiss AxioObserver Yokogawa CSU-X1 spinning disk confocal microscope with a 60x oil objective. Images at 15 randomly selected locations were captured by ZEN2 (blue edition) of each well. For each location, a stack of images was taken from the bottom of the cells to the top of the cells with a stack height of 0.27 µm.

Supplementary Note S6: Quantifying the average amount of nanotube seeds attached nonspecifically per HeLa cell

Here we used two methods to quantify the amount of nanotube seeds that nonspecifically attached to a cell membrane: (1) use the average fluorescent intensity of seeds per cell as a measure of the amount of seeds nonspecifically attached to a cell membrane, (2) use the average number of seeds per cell as a measure of the amount of seeds nonspecifically attached to a cell membreane.

6.1 Measurement of the average fluorescent intensity of seeds per cell

From z-stack images taken at a given position, one stack image was chosen near the center of the cell in which the fluorescence from the seeds attached on the substrates was not visible but the cross-sectional area of the cell was sufficiently large to allow for accurate quantification.

The total number of cells (N_{cell}) in the chosen stack image at each location was first counted manually. Although the cells were not stained in these samples, the cell showed visible autofluorescence under the 488nm laser which we used to locate the positions of each cell and to count the number of cells. For the cells crossing the image edge such that they were only partially in the image, we only counted those which were on the top and right edges of the image and where the fraction of the cell in the image was obviously larger than half.[6]

Then the total fluorescence intensity of seeds ($I_{total,S}$) at each location were quantified by processing the chosen stack image at each location by custom MATLAB scripts. The chosen stack image at each location was imported for analyzing and the thresholds were set as below:

$$Threshold_{min,S} = 700 \tag{1.1}$$

$$Threshold_{max,S} = 3000 \tag{1.2}$$

The $Threshold_{min,S}$ was the background from cell autofluorescence in seeds channel in example images from the area of cell without seeds attachment. The $Threshold_{max,S}$ was the intensity of aggregated seeds. Only pixels with intensity in this range were counted.

For each pixel x in this range, its intensity I_x included both background intensity and additional intensity that was due to the fluorescence of seeds:

$$I_x = I_{x,S} + Threshold_{min,S} \tag{1.3}$$

$I_{x,seeds}$ was the fluorescence intensity due to seeds in pixel x.

The total fluorescence intensity of seeds at each location was calculated by summing the seeds fluorescence intensity of all pixels in the chosen stack image:

$$\begin{aligned} I_{total,S} &= \sum I_{x,S} \\ &= \sum (I_x - Threshold_{min,S}) \end{aligned} \tag{1.4}$$

For each location, the chosen stack image was processed by a MATLAB script as described above to get $I_{total,S}$. The average fluorescence intensity per cell $I_{average,S}$ at each location was then calculated as:

$$I_{average,S} = \frac{I_{total,S}}{N_{cell}} \tag{1.5}$$

For samples of nanotube seeds with and without PEG coating at each concentration (4 pM, 8 pM, 16 pM and 32pM), 15 chosen stack images from different locations for each sample were processed simultaneously by a MATLAB script and the average fluorescence intensity per cell at each location was calculated as described above. Error bars for the average fluorescent intensity per cell for each sample represent the 95% confidence intervals of proportions.

6.2 Measurement of the average number of seeds per cell

From z-stack images taken from the cell bottom to the cell top at a given position, a maximun intensity z projection image was generated using ImageJ software. The first 1-4 stack images were excluded during projection image generation to exclude both

seeds that were attached to the substrate (*i.e.* glass) and seeds for which it was hard to determine whether they were attached to the cell membrane or the substrate.

The total number of cells (N_{cell}) in the z projextion image at each location was determined as described above in Supplementary Note 6.1.

The total number of seeds (N_{seeds}) in the z projection image at each location was counted using TrackMate[7] software (v3.4.2) incorporated into ImageJ. TrackMate is designed to be used as a spot detector for single particle localization and single particle tracking and has been used as a tool to count the number of nanoparticles and their brightnesses in other work.[7] To count the total number of seeds, the z projection image at each location was imported into ImageJ and analyzed in the TrackMate panel. Since the approximate size of a seed was 5 or 6 pixels (1 pixel=0.212 microns), the LoG detector was selected, which is a method designed for intermediate spot sizes, *i.e.* those between 5 and 20 pixels in diameter. The LoG detector applied plain LoG (Laplacian of Gaussian) segmentation to the image. The "Estimated blob diameter" was initially set to 1.06 microns to detect the seeds. This parameter was adjusted to check that the filter identified all of the seeds that were visible without identifying false positives (as deemed manually).

The average number of seeds per cell was then calculated by dividing the total number of seeds by the total number of cell.

$$N_{average,Seed} = \frac{N_{seeds}}{N_{cell}} \qquad (1.6)$$

For samples of nanotube seeds with and without PEG coating at each concentration (4pM, 8pM, 16pM and 32pM), 15 chosen stack images from different locations for each sample were processed and the average number of seeds per cell at each location was calculated as described above. Error bars for the average fluorescent intensity per cell for each sample represent 95% confidence intervals of proportions.

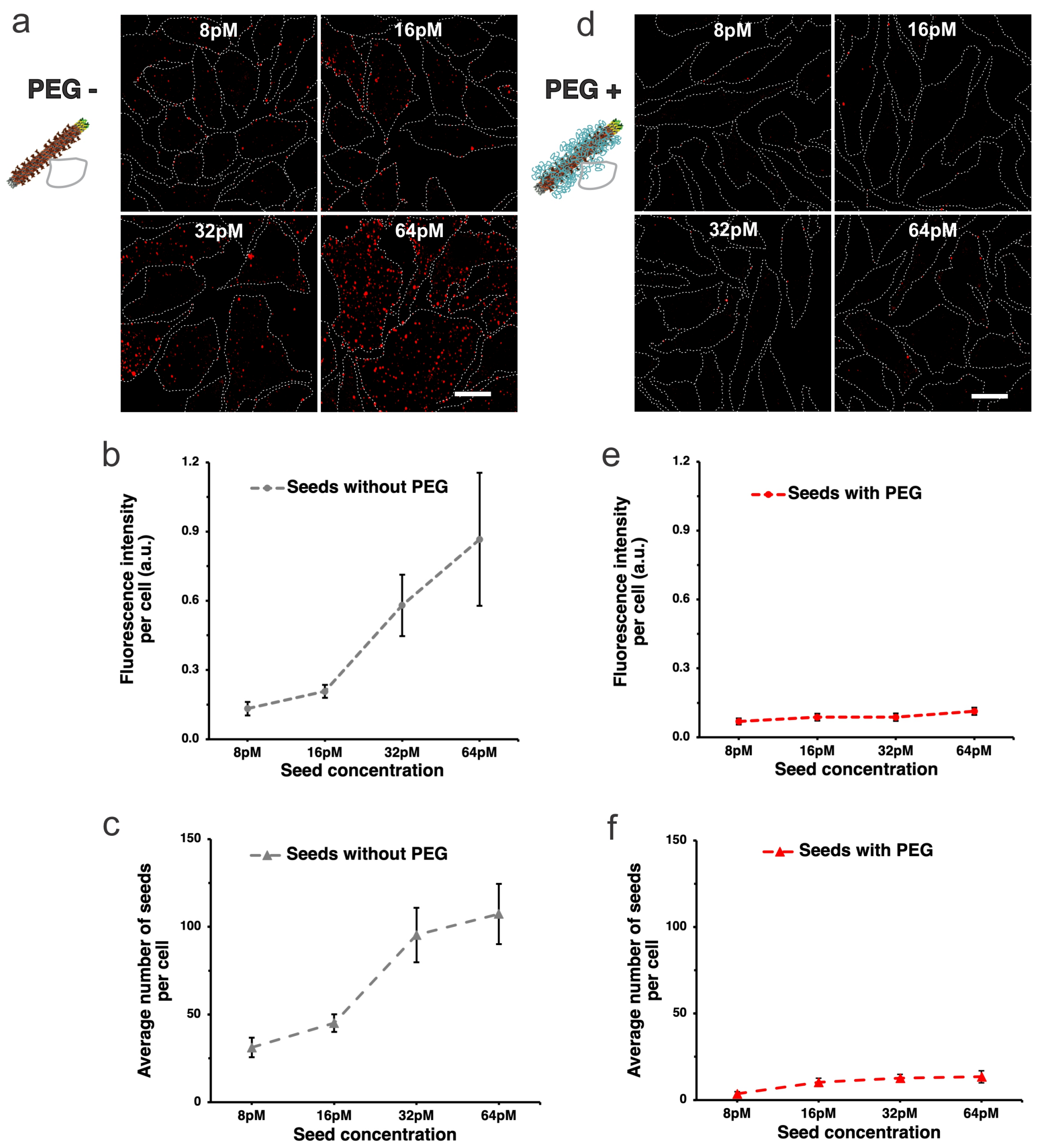

Supplementary Figure S4: Coating seeds with PEG reduces the extent of nonspecific binding between seeds and the HeLa cell membrane. The experiment was performed as described in Supplementary Note S5. Scale bars: 20µm. **a.** 3D projection images of HeLa cells after being incubated with different concentrations (8, 16, 32, 64 pM) of nanotube seeds without PEG coating. Seeds were labeled with Atto488. The cells were located by their autofluorescence. **b.** The average fluorescence intensity of HeLa cells after seeds without PEG coating were attached, as measured by mean fluorescence intensity per cell (N=15 fields of view). **c.** The average number of seeds per Hela cell after seeds without PEG coating were attached (N=15 fields of view). **d.** 3D projection images of HeLa cells after being incubated with different concentrations of nanotube seeds with PEG coating (8, 16, 32, 64pM). **e.** The average fluorescence intensity of HeLa cells after seeds with PEG coating were attached, as measured by mean fluorescence intensity per cell (N=15 fields of view).

f. The average number of seeds per Hela cells after seeds with PEG coating were attaced (N=15 fields of view). Error bars here are 95% confidence intervals.

Supplementary Note S7: Protocol for growing 6nt seeded nanotubes

The process for growing 6nt seeded nanotubes consists of two steps. First, annealing nanotube monomer strand mixes are annealed until the temperature reaches 37°C, to form monomers. Second, nanotube seeds are added to the monomers to template the growth of nanotubes on the seeds' ends.

7.1 Assembly of DNA nanotubes without PEG coating

Supplementary Table S4: Recipe for 6nt monomer (Cy3 labeled) without PEG

Monomer mix	Final desired concentration (nM or fold)	Stock concentration (nM or fold)	To add (µl)
H_2O			14.7µl
TAE-Mg^{2+} buffer	1x	10x	2µl
Full 6nt monomer strands (Cy3)	150nM	1000nM	3µl
Total			19.7µl

Full 6nt monomer strands (Cy3): A mixture containing all five SEs strands (see Supplementary Figure S34a) in water each at a concentration of 1µM, except for the strands with sticky ends (SEs_2 and SEs_4) each at a concentration of 2 µM.

The monomer solution was first mixed according to the recipe above (no seeds were added).

Purified seeds were prepared and their concentration measured as described in Methods Supplementary Note S1.

19.7µl of the monomer solution was annealed from 90°C to 37°C using the same annealing schedule as for annealing seeds (Supplementary Note S1 step 2). When the solution reached 37°C, 0.3 µl of seed solution (0.4nM) was added to 19.7 µl of monomer solution so that the monomer concentration was 147.8 nM after the addition of seeds and the final concentration of seeds was 6 pM. The mixture was then held at 37°C for another 15 hours to let the nanotubes grow from the seeds.

7.2 Assembly of DNA nanotubes with PEG coating

Nanotube seeds were assembled and coated with PEG as described in Supplementary Notes S1 and S3. The concentration of the seed solution was adjusted to 0.4nM by adding 1xTAE- Mg^{2+} buffer.

The monomer solution was mixed according to the recipe below.

Supplementary Table S5: Recipe for 6nt monomers (Cy3 labeled) with PEG

	Final desired concentration (nM or fold)	Stock concentration (nM or fold)	Volume added
H_2O			7.8 µl
TAE-Mg^{2+} buffer	1x	10x	2 µl
SEs_3-PEG	450 nM	10000 nM	0.9 µl
The remaining 6nt monomer strands (1,2,4,5)	450 nM	1000 nM	9 µl
Total			19.7 µl

SEs_3_PEG: The strand SEs_3-5'Cy3-3'amine (Supplementary Figure S34b) conjugated with PEG in water.

The remaining 6nt monomer strands (1,2,4,5): A mixture containing the SEs strands SEs_1 and SEs_5 in water each at a concentration of 1µM, and the SE strands SEs_2 and SEs_4 each at a concentration of 2 µM (see Supplementary Note S50.1 and Supplementary Figure S34b).

19.7µl of monomer solution was annealed from 90°C to 37°C using the annealing schedule in Supplementary Note S1 step 2. When the solution reaches 37°C, 2 µl of PEG coated seed solution (0.4nM) was added to 19.7 µL of monomer solution so that the monomer concentration was 415 nM after the addition of seeds and the seeded final concentration is 37 pM. The mixture was held at 37°C for at least another 3 days to let the nanotubes grow from the seeds.

7.3 Protocols for measuring the lengths of nanotubes

Seeded nanotubes with and without PEG coating were imaged under an epi-fluorescence microscope and the nanotubes' lengths were measured manually in these images.

The seeded nanotubes without PEG coating were prepared as described in Supplementary Note 7.1 and imaged under epi-fluorescence microscope after addition of nanotube seeds (no PEG coating) and incubation at 37°C for 1 day. To avoid a significantly number of nanotubes that overlapped one another in an image, nanotube samples were diluted 10-fold with TAE-Mg^{2+} buffer before they were added to the coverslip. The seeded Supplementary Note 7.2 and imaged after addition of nanotube seeds (with PEG coating) and incubation at 37°C for 3 days. The nanotubes were diluted 50-fold with TAE-Mg^{2+} buffer before they were added to the coverslip. Both kinds of diluted seeded nanotubes were imaged under an inverted epi-fluorescence microscope (Olympus IX71) with a 60x/1.45 NA oil immersion objective lens by using Andor SOLIS (Oxford Instruments) software. Images of seeds and nanotubes were taken under corresponding fluorescence channels (atto647 and Cy3 individually) at several random locations for each kind of seeded nanotube.

The lengths of seeded nanotubes were measured using the method described in Li *et al*[2]. Composite two-color images of seeded nanotubes for each location were created by merging the images from the atto647 and Cy3 channels. To maximize the accuracy of the length determination process, the contrast of each color in the composite image was enhanced using linear histogram stretching with ImageJ software. Nanotubes without seeds attached and those few that were tangled together, making individual nanotubes hard to distinguish, were both excluded. Seeded nanotubes that were not entirely within the field of view were also excluded. The length of a seeded nanotube in a given composite image was measured by manually drawing segmented lines along the nanotube curves, from the seeds to the tip of nanotube, in ImageJ software. Seeded nanotubes whose measured length were less than 0.5μm (about 3 pixels) were counted as having length 0.

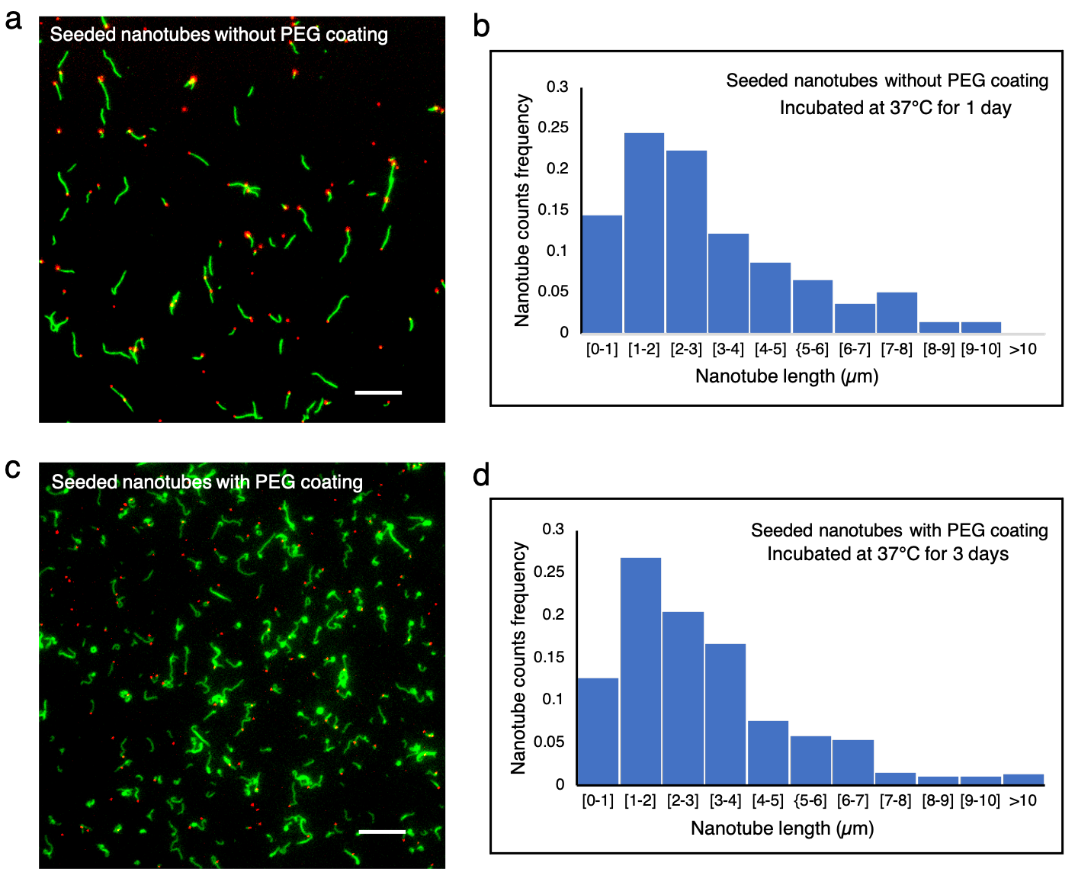

Supplementary Figure S5: Sample epi-fluorescence micrographs and length distributions of seeded nanotube with 6 nucleotide sticky ends. Seeds were labeled with atto647 (red); monomers with Cy3 (green). Scale bars are 20 µm. **a-b.** Epi-fluorescence micrograph (**a**) and histogram graph of length distribution (**b**) of nanotubes without PEG coating grown and incubated in 37°C after adding nanotube seeds for 1 day. (N=139 nanotubes in three fields of view were measured). In other studies, nanotubes without PEG grown did not increase significantly in length when incubation was extended more than 1 day.[8,9] **c-d.** Epi-fluorescence micrograph (**c**) and histogram graph of length distribution (**d**) of nanotubes with PEG coating grown and incubated in 37°C after adding nanotube seeds for 3 days. (N=396 nanotubes in ten fields of view were measured).

Supplementary Note S8: Characterizing the extent of nonspecific binding between seeded DNA nanotubes and live HeLa cells

(1) Seeded nanotube preparation:

The PEG coated nanotubes were grown from seeds coated with and without PEG. 6nt DNA nanotube seeds labeled with atto488 were assembled by mixing the components listed in Supplementary Table S1 as in Supplementary Note S1, then annealed as described in Supplementary Note S1 step 2, and purified as in Supplementary Note S1 step 3. The seeds were then separated into two aliquots. One aliquot was used as it was as the seeds without PEG coating and the other aliquot was used to prepare PEG-coated seeds as described in Supplementary Note S3. The concentrations of the seeds with and without PEG coating were measured and calculated as described in Supplementary Note S1 step 3 and adjusted to 0.4nM by diluted with 1x TAE-Mg^{2+} buffer.

To grow PEG coated seeded nanotubes, 14 aliquots of 19.7μL monomer mix was prepared as described in Supplementary Note 7.2 (Supplementary Table S5). When the temperature reached 37°C, 2μL seeds without PEG coating (0.4nM) were added to 7 of the aliquots of 19.7 μL monomer individually. And 2μL seeds with PEG coating (0.4nM) were added to each of the other 7 aliquots of 19.7 μL monomer individually. All of the seeded nanotubes were then incubated at 37°C for 3 days.

(2) HeLa cell preparation:

HeLa cells were cultured and prepared as described in step 2 of Supplementary Note S5 and all buffers were kept on ice.

(3) Incubation of cells with 1% BSA solution:

The medium in each well of cells was exchanged to cold 250 μL 1% BSA (DMEM) buffer. The cells were then incubated in the refrigerator (4°C) for 5 minutes.

(3) Incubation of seeded DNA nanotubes with HeLa cells:

Cold 125 μL 1% BSA (DMEM) buffer with 12mM $MgSO_4$ was added to 125 μL of the seeded nanotubes grown from seeded with and without PEG coated individually (*i.e.* the seeds were diluted one-fold). The 1% BSA (DMEM) buffer in each well was exchanged with 250 μL of the diluted nanotube solution individually. The cells were incubated in a refrigerator (4°C) for 2 hours with gentle pipetting every 30min to re-suspend the nanotubes.

(4) Washing of cells to remove unattached seeded nanotubes:

After 2 hours, each well with cells was washed with cold DMEM-12mM Mg SO_4 buffer 3 times in the same manner as described in step 5 of Supplementary Note S5 to remove the nanotubes not attached to the cells.

(5) Fixing of cells and imaging under a spinning disk confocal microscope:

The cells were fixed and imaged under spinning disk confocal microscope as described in Supplementary Note S5 steps 6-7.

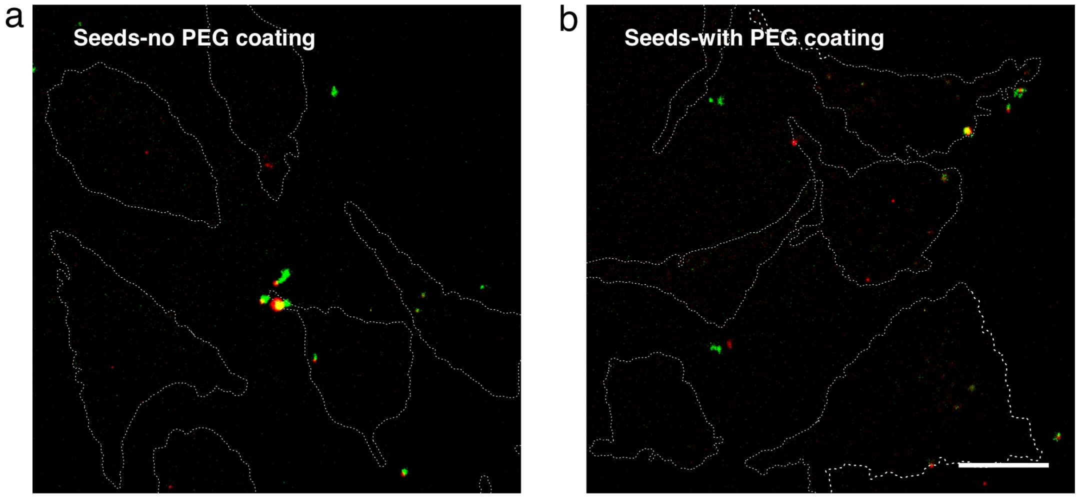

Supplementary Figure S6: PEG-coated nanotubes do not bind nonspecifically to the HeLa cell surface. Experiments were performed as described in Supplementary Note S8. The nanotubes were labeled with Cy3 (green). The cells were located by their autofluorescence. Scale bars 20μm. **a.** Maximum intensity projection images of HeLa cells incubated with nanotubes grown from unmodified seeds (no PEG coating). Less than one seeded nanotube nonspecifically attached on each cell averagely (0.70±0.27 nanotube per cell (N=58)) **b.** Confocal stack micrograph of HeLa cells incubated with nanotubes grown from PEG-coated seeds. Also, less than one seeded nanotube nonspecifically attached on each cell averagely (0.77±0.20 nanotube per cell (N=75))

Section 2: SpyTag-SpyCatcher chemistry:

Supplementary Note S9: Conjugation of the SpyTag peptide to a DNA strand

The azide modified SpyTag peptide was conjugated to an amine-modified DNA strand that binds to the origami seed, Amine_DNA_SpyTag (see Supplementary Note 41.5) using a "copper-free click" reaction. These methods were adopted from Stephanopoulos *et al.*[10] The scheme used here is shown in Supplementary Figure S7a. The azide-SpyTag peptide was synthesized by BioSynthesis (Lot No. P3130-1) and its sequence is F^{az}-GGAHIVMVDAYKPTK, where Faz denotes the unnatural amino acid 4-azido-L- phenylalanine.

(1) The 3' amine modified DNA strand was first reacted with dibenzocyclooctyne-sulfo-N-hydroxysuccinimidyl ester (DIBAC-sulfo-NHS) to generate the DNA-DIBAC conjugate by mixing the reagents in Supplementary Table S6.

Supplementary Table S6: Reagents for the synthesis of the DNA strand-DIBAC conjugate.

Reagents name	Stock concentration	To add
Amino_DNA_SpyTag (Supplementary Note 41.5)	1mM in water	20 µL
phosphate buffer (pH 8.5)	100 mM	4 µL
dibenzocyclooctyne- sulfo-N-hydroxysuccinimidyl ester (DIBAC-sulfo-NHS)	100 mM in DMSO	2.67 µL
Total		26.67µL

The mixture was incubated for 2 hours at room temperature via vigorous shaking.

(2) After 2 hours, the excess DIBAC-sulfo-NHS molecules was removed using a size exclusion spin column (Illustra Microspin G-25, GE Healthcare) which was pre-equilibrated with 20 mM of phosphate buffer (pH: 7.5) before adding the reaction mixture. Approximately 27µL of the purified DNA-DIBAC was recovered.

(3) 50 µL of the azido-Spytag (1 mM solution in water) and 8.5 µL sodium chloride solution (1M in water) were added to the above purified DNA-DIBAC solution and then the reaction mixture was gently agitated overnight.

(4) The Spytag-DNA conjugate was purified by PAGE gel by following the methods for purifying the DNA-PEG conjugate given in Supplementary Note 2.2.

(5) The concentration of the SpyTag-DNA conjugate was measuring by following the methods used to measure the concentration of the PEG-DNA conjugate for coating seeds given in Supplementary Note 2.3.

Supplementary Note S10: Transfection of HeLa cells with GFP-integrin-SpyCatcher, GFP-integrin plasmids

Materials:

Plasmid DNA: The GFP-integrin-SpyCatcher plasmids were constructed by inserting the SpyCatcher DNA sequence into the GFP-integrin construct using the NotI restriction site. The backbone of the plasmid is a Clontech vector with kanamycin resistance. The sequence of the plasmids is in the Supplementary Note S51. The plasmid was transformed and amplified in DH5alpha bacteria, and amplified using a Qiagen miniprep kit. Both plasmids DNA were diluted to 1mg/mL for the following transfection process. The GFP-integrin plasmids were used as controls.

(1) Plasmid DNA preparation:

Both the GFP-integrin-SpyCatcher plasmids and GFP-integrin plasmids were prepared. For each plasmid DNA, 2 μL of the 1 mg/mL plasmid DNA was added to 38 μL 1x Opti-MEN (31985062, ThermoFisher) in a 1.5mL Eppendorf tube and pipetted several times gently to mix well. To this solution, 2 μL of the X-tremeGENE 9 DNA Transfection Reagent (6365779001, Sigma Aldrich) was added and again mixed well with a pipette. The mixture was incubated at room temperature for 30 minutes.

(2) HeLa cell preparation:

The HeLa cells were passaged as described in the online methods. The cells were counted by hemacytometer and diluted to a cell concentration of 2.4×10^5 cells per mL by cell growth medium (DMEM medium containing 10% FBS and 1% Penicillin-Streptomycin).

(3) Transfection of Hela cell:

After 30 minutes, 42 μL of the GFP-integrin-SpyCatcher plasmids mix from step 1 was combined with 500 μL of the diluted cells from step 2 in a 1.5mL Eppendorf tube. The solution was mixed well by gently inverting the tube around 20 times. This cell solution was then pipetted into the wells of an 8-well chamber with 250 μL for each well. These steps were repeated to add the GFP-integrin plasmids to the cell and seed them into the wells as control. All the cells were then returned to the incubator for 2 days and used as cultured after this incubation period. Cells were transfected with both the GFP-integrin-SpyCatcher plasmids and GFP-integrin plasmids.

Supplementary Note S11: Attachment of SpyTag-modified DNA seeded nanotubes to HeLa cells expressing the GFP-integrin-SpyCatcher transgene

(1) Seeded nanotube preparation:

Atto647 labeled 6nt nanotube seeds with and without SpyTag on the seeds' ends were assembled by mixing the components listed in Supplementary Table S7 and S8 individually, then annealed as described in Supplementary Note S1 step 2, purified as in Supplementary Note S1 step 3. The concentrations of the seeds with and without SpyTag were determined as described in Supplementary Note S1 step 3 and adjusted to 0.4nM by diluting with 1xTAE-Mg^{2+} buffer.

Supplementary Table S7: 6nt nanotube seeds with SpyTag modification

Seed Assembly Mixture	Final desired concentration (nM or fold)	Stock concentration (nM or fold)	To add (µl)
H_2O	--	--	60.6 µl
TAE-Mg^{2+} buffer	1x	10x	10 µl
Seed staple strands mix	250nM	4167nM	6 µl
Seed A adapter strands mix (6nt)	100nM	4167nM	2.4 µl
M13mp18 scaffold strand	5nM	100nM	5 µl
Amine left side attachment strands mix	20nM	1000nM	2 µl
SpyTag-DNA conjugate	120nM	1000nM	12 µl
96 Seed labeling attachment strands mix	10nM	1000nM	1 µl
Labeling strand ATTO647	1000nM	100000nM	1 µl
Total			100 µl

Seed A adapter strand mix (6nt): A mixture containing all 24 Seed A adapter strands for 6nt nanotubes (see Supplementary Note S50.3 and Supplementary Figure S43) in water each at concentration 4.176µM (100µM/24).

Amine left side attachment strands mix: A mixture containing all six amine left side attachment linker strands for seed A (Amine_leftside_01 to Amine_leftside_06) in water each at 1000nM (see Supplementary Note S50.5 and Supplementary Figure S50).

SpyTag-DNA conjugate: The strand Amine_DNA_SpyTag (Supplementary Note S50.5) conjugated with SpyTag peptide in water.

Labeling strand ATTO647: The strand Labeling strand ATTO647 in water at 100000nM. (see Supplementary Note S50.2)

The nanotube seeds assembled using the recipe in Supplementary Table S7 have 6 SpyTag groups on the left end of each seed barrel (as illustrated) and were labeled with atto647. Nanotubes grow from the right sides (as illustrated) of these seeds.

Supplementary Table S8: 6nt nanotube seeds without SpyTag modification

Seed Assembly Mixture	Final desired concentration (nM or fold)	Stock concentration (nM or fold)	To add (µl)
H_2O	--	--	72.6 µl
TAE-Mg^{2+} buffer	1x	10x	10 µl
Seed staple strands mix	250nM	4167nM	6 µl
Seed A adapter strand mix (6nt)	100nM	4167nM	2.4 µl
M13mp18 scaffold strand	5nM	100nM	5 µl
Amine left side attachment strands mix	20nM	1000nM	2 µl
96 Seed labeling attachment strands mix	10nM	1000nM	1 µl
Labeling strand ATTO647	1000nM	100000nM	1 µl
Total			100 µl

The nanotube seeds assembled using the recipe in Supplementary Table S8 have 6 DNA binding sites for SpyTag conjugates on the left end of each seed barrel (as illustrated) and were labeled with atto647. Nanotube grow from the right sides (as illustrated) of these seeds.

To grow seeded nanotubes, 10 aliquots of 19.7µL monomer mix were prepared as described in Supplementary Note 7.2 (Supplementary Table S5). When the temperature reached 37°C, 2µL seeds without SpyTag (0.4nM) were added to each of 5 aliquots of the 19.7 µL monomer solutions. 2µL seeds with SpyTag (0.4nM) was added to each of the other 5 aliquots of 19.7 µL monomer. All of the seeded nanotubes were then incubated at 37°C for 3 days.

(2) HeLa cell preparation:

HeLa cells transfected with GFP-integrin-SpyCatcher and GFP-integrin (as a control) were each added to two wells of 8-well chambers individually as described in Supplementary Note S10.

(3) Incubation of the seeded nanotubes with and without SpyTag with cell.

To reduce the nonspecific attachment of seeded nanotubes to the Eppendorf tubes, 1mL 1% BSA (suspended in water) as added to each of the two 1.5mL Eppendorf tubes and incubated at room temperature for 5min. After 5min, the 1% BSA solution was removed from the two Eppendorf tubes and 300 µL of DPBS (14190144, ThermoFisher) with 12 mM $MgSO_4$ buffer was added to each tube. Then either 200 µL seeded nanotubes with SpyTag or 200 µL seeded nanotubes without SpyTag was added to to each Eppendorf tube.

Before the seeded nanotube were added to the cells, the wells of cells transfected with either GFP-integrin-SpyCatcher or GFP-integrin were washed with 250 µL DPBS buffer for 3 times. During each wash cycle, the solution in a well was carefully removed and 250 µL fresh DPBS buffer was immediately added. When the DPBS buffer was removed during the third wash cycle, 250 µL of the diluted solutions of

nanotubes grown from seeds with and without SpyTag was added to each well of cells transfected with GFP-integrin-SpyCatcher and GFP-integrin. Then all the wells of cells were put back into the cell culture incubator and incubated at 37 ºC for 30 minutes. After 30 minutes, the wells of cells were washed with DPBS containing 12 mM with $MgSO_4$ 3 times. During each wash cycle, the solution in the well was carefully removed and 250 µL fresh DPBS buffer with 12mM $MgSO_4$ was immediately added.

(4) Fixing of cells and imaging under a spinning disk confocal microscope:

The cells were fixed by 4% paraformaldehyde and imaged under spinning disk confocal microscope as described in Supplementary Note S5 steps 6-7.

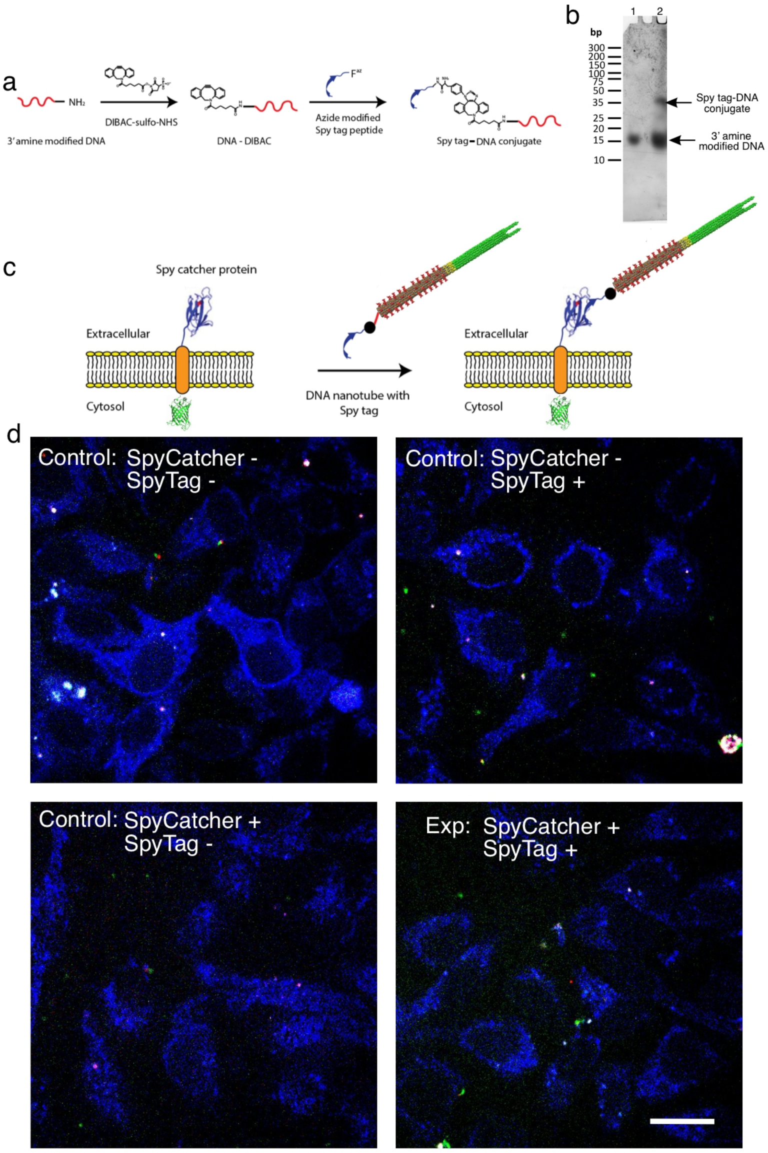

Supplementary Figure S7: Anchoring SpyTag-conjugated, seeded nanotubes to HeLa cells with GFP-integrin-SpyCatcher expressed on their membranes. Experiments were performed as described in Supplementary Note S11. Blue: GFP, red: seeds labeled with atto647, green: nanotubes labeled with Cy3. Scale bar: 20µm. **a.**

Scheme for conjugating a DNA strand to the SpyTag peptide. **b.** A PAGE gel showing that the Spytag-DNA conjugation runs more slowly than the unconjugated amine-modified DNA. L1: 3' amine modified DNA strand, L2: the reaction mixture after conjugation of Spy-tag and 3'amino DNA before gel purification. This experiment was repeated more than three times independently with similar results. **c.** Scheme for anchoring seeded nanotubes to the cell membrane via the SpyCatcher-SpyTag interaction. The cells were transfected with a GFP-integrin-SpyCatcher plasmid designed to present the SpyCatcher protein outside the cell membrane. The SpyTag-DNA conjugate was attached to the ends of nanotube seed ends (Supplementary Figure S46). **d.** Confocal stack micrographs of HeLa cells after attaching the seeded nanotubes to the cell membrane by following the protocol in Supplementary Note S6. Controls: either SpyCatcher was not present on the cell membrane or SpyTag was not present at the ends of the seeded nanotubes or both. Experiment: SpyCatcher is presented by the cell and SpyTag is presented at the ends of the seeded nanotubes. No significant difference in the number of nanotubes attached per cell was observed between any of the control groups and the experimental group. This experiment was repeated more than three times independently with similar results.

Supplementary Note S12: Estimation of the binding flux of DNA nanotubes on the cell surface usming SpyCatcher-SpyTag binding

SpyCatcher and SpyTag form a covalent bond whose unbinding rate was assumed to be negligible. The rate of binding of SpyTag modified seeded nanotubes to the cell surface should proceed via the reaction:

$$S + R \rightarrow Conjugate \tag{2.1}$$

where S is the SpyTag labeled, seeded nanotube, R is the cell receptor presenting the SpyCatcher protein and the conjugate is the receptor with a bound nanotube.

Our first goal was to estimate the rate at which a nanotube would bind to a particular receptor to form a conjugate. That rate is given by $k[S]$ where k is the previously measured second-order rate constant for SpyTag and SpyCatcher at the cell surface k = $1.4\text{x}10^3 \pm 43$ M^{-1} s^{-1}.[11] 15-20 pM of seeds for seeded nanotubes were added to cells (Supplementary Note S18). When 20 pM of seeds are added, the rate of attachment should be $2.1\text{x}10^{-8}$ s^{-1} or $7.6\text{x}10^{-5}$ hr^{-1}.

We estimate the concentration of the SpyCatcher at 10^4 copies per cell.[12] Using this value and the rate of seed attachment, we estimate that net rate of nanotube attachment to cells would be 0.76 nanotubes/cell/hr.

The attachment rate of the seeds is particularly low because the concentration of seeds added was very small. These small concentrations of seeds are consistent with standard methods of preparing seeded DNA nanotubes.[8,13] While nanotubes might conceivably be concentrated after preparation, their very large size means that it would be challenging to increase these concentrations by more than 1-2 orders of magnitude.

Section 3. Antibody-mediated DNA nanotube anchoring (AMDA) method:

Supplementary Note S13: Staining fixed HeLa cells with EGFR antibodies at room temperature

(1) HeLa cell preparation:

HeLa cells were seeded in 2 wells of an 8-well chamber overnight with 4 x10^4 cells per well as described in step 2 of Supplementary Note S5.

(2) Fixing of cells:

The next morning, two wells of cells were each washed twice with 250 µL DPBS buffer and fixed with 250 µL 4% paraformaldehyde (in PBS) buffer as described in the step 6 of Supplementary Note S5.

(3) Incubation of cell with 1% BSA solution:

The solution in each well containing cell was exchanged to 250 µL 1% BSA in DPBS buffer and incubated at room temperature for 30 minutes.

(4) Stain HeLa cell with EGFR primary antibody:

2.5µL of EGFR primary antibodies stock solution at 0.2mg/mL was diluted to 2µg/mL by adding it to 247.5µL 1% BSA in a DPBS buffer. After 30 minutes, the solution in one well of fixed HeLa cells were replaced by this 250 µL diluted EGFR primary antibodies solution, while the other well was replaced by 250 µL 1% BSA in a DPBS buffer as control. Both wells of cells were then incubated at room temperature for 1 hour.

(5) Cell washing:

After 1 hour of incubation, each well was washed with fresh DPBS buffer 3 times to remove extra unattached EGFR primary antibody. For each wash cycle, the solution in the well was carefully removed and 250 µL fresh DPBS buffer was immediately added with pipette. The cells were gently shaken at room temperature for 5 minutes after adding fresh DPBS buffer in the second and third wash cycles.

(6) Staining of HeLa cells with Alexa 647 labeled secondary antibody:

2.5µL of Alexa 647 labeled secondary antibody stock solution at 2mg/mL was diluted to 10µg/mL by adding it to 497.5µL of 1% BSA in a DPBS buffer. The solution in each well with fixed HeLa cells was replaced by 250 µL diluted secondary antibodies solutions. Both wells of cells were then incubated at room temperature for 1 hour and washed with DPBS buffer 3 times as described in step 5 to remove the unattached secondary antibody.

(6) Imaging of cells under a spinning disk confocal microscope:

Each of the wells of cells were imaged using a spinning disk confocal microscope as described in the step 7 of Supplementary Note S5. 5 random locations were imaged in each well.

Supplementary Note S14: Staining live HeLa cells with EGFR antibodies at room temperature

The protocol for staining live HeLa cells with EGFR antibodies at room temperature was the same as the protocol described in Supplementary Note S13 except that the two wells of cells were each only washed twice with DPBS buffer and were not fixed by 4% paraformaldehyde (in PBS) buffer in step 2.

Supplementary Note S15: Staining live cells with EGFR antibodies at 4ºC

(1) HeLa cell preparation:

HeLa cells were seeded in two wells of an 8-well covered-glass chamber overnight, with 4 x10^4 cells per well as described in step 2 of Supplementary Note S5. The next morning, the cells were moved to the refrigerator (4ºC) and incubated for 10 minutes. All buffers used in the following steps (DPBS, 1% BSA in DPBS buffer) and antibody solutions were then put on ice. The chambers were moved out of the refrigerator and placed on ice. They were also kept on ice during all of the following steps when the cells were not in the refrigerator for incubation.

(2) Incubation of cells with 1% BSA solution:

The medium in each well was replaced by 250 µL cold 1% BSA in a DPBS buffer and both wells of cells were incubated in the refrigerator for 30 minutes.

(3) Staining of live HeLa cell with EGFR primary antibody at 4ºC

2.5µL of EGFR primary antibody stock solution at 0.2mg/mL was diluted to 2µg/mL by adding it to 247.5µL 1% BSA in a DPBS buffer. After 30 minutes, the solution in one well of live HeLa cells were replaced by this 250 µL diluted EGFR primary antibodies solution, while the other well was replaced by 250 µL 1% BSA in a DPBS buffer as control. Both wells of cells were incubated in the refrigerator (4ºC) for 1 hour.

(4) Washing of live HeLa cells on ice:

After 1 hour of incubation, the wells with cells were moved onto ice and each well was washed with cold fresh DPBS buffer 3 times to remove the extra unattached EGFR primary antibody. For each wash cycle, the solution in the well was carefully removed and 250 µL cold fresh DPBS buffer was immediately added using a pipette. The cells on ice were gently shaken for 5 minutes after adding the fresh DPBS buffer during the second and third wash cycles.

(5) Staining of live HeLa cells with Alexa 647 labeled secondary antibody at 4ºC:

2.5µL of Alexa 647 labeled secondary antibody stock at 2mg/mL was diluted to10µg/mL by adding it to 497.5µL of 1% BSA in a DPBS buffer. The solution in each well of live HeLa cells were replaced by 250 µL diluted secondary antibodies solutions individually. Both wells of cells were incubated in the refrigerator for 1 hour and washed by DPBS buffer 3 times on ice as described in step 4.

(6) Imaging of cells under a spinning disk confocal microscope:

Each of the wells of cells were imaged using the spinning disk confocal microscope as described in the step 7 of Supplementary Note S5. 5 random locations were imaged in each well.

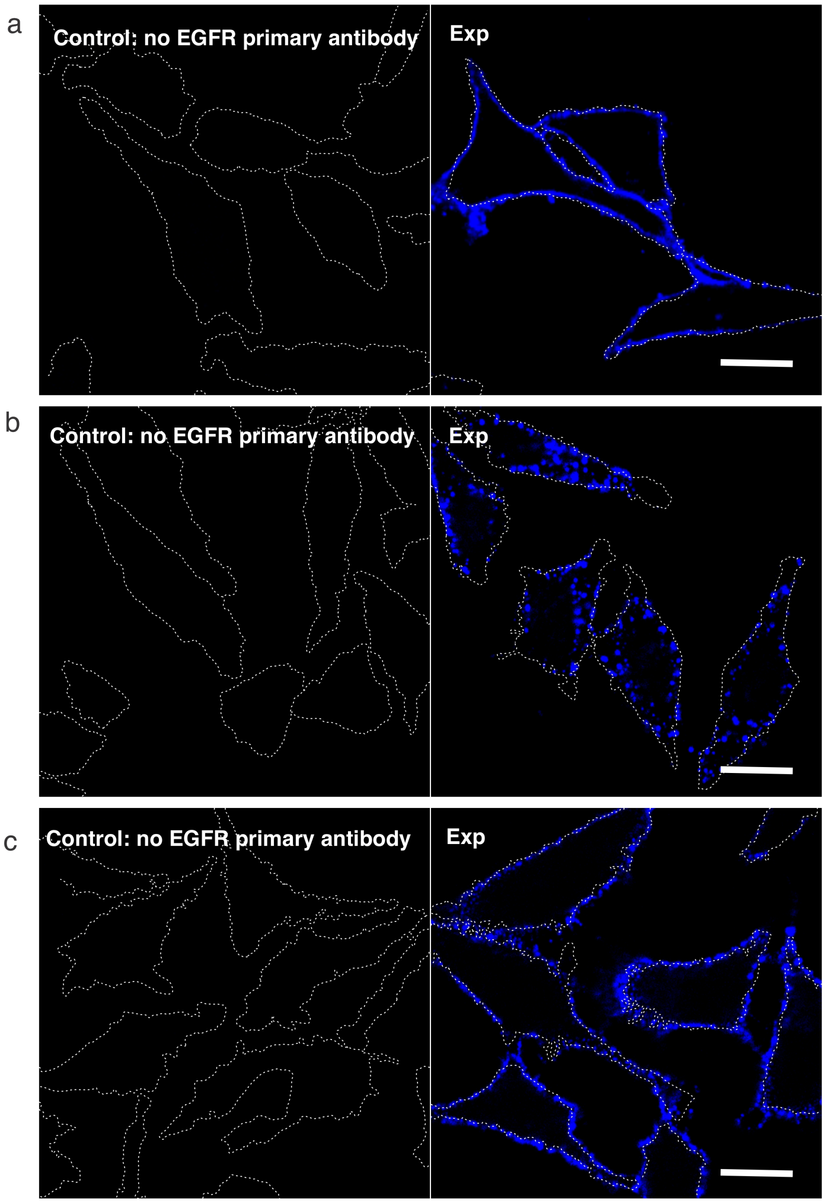

Supplementary Figure S8: **Temperature-dependent persistence of EGFR antibody labels on live cells.** Experiments were performed as described in Supplementary Notes S13, S14 and S15. Confocal stack images of HeLa cells stained with primary EGFR antibodies and Alexa647 labeled secondary antibodies (blue). The cells were located by their autofluorescence. Scale bars 20μm. **a.** Micrographs of HeLa cells that were first fixed and then labeled. **b.** Micrographs of HeLa cells that were labeled and then imaged at room temperature (about 19~21°C). **c.** Micrographs of HeLa cells that were labeled at 4°C and imaged immediately afterwards. This experiment was repeated more than three times independently with similar results.

Supplementary Note S16: Attachment of secondary antibody-labeled DNA nanotube seeds to EGFR receptors labeled with primary antibodies on the HeLa cell membrane

(1) Preparation of DNA nanotube seeds:

DNA nanotube seeds with 6 biotinylated DNA strands on the seeds' free ends were assembled by mixing the components listed in Supplementary Table S1 as in Supplementary Note S1, then annealed as described in Supplementary Note S1 step 2, purified as in Supplementary Note S1 step 3 and coated with PEG as described in Supplementary Note S3. The concentrations of the seeds with PEG coating were determined as described in Supplementary Note S1 step 3 and adjusted to 0.4nM by diluting with 1x TAE-Mg^{2+} buffer.

(2) Conjugating secondary antibody to streptavidin

Alexa 647 labeled secondary antibody (2AB) was conjugated to streptavidin (STA) using a commercial streptavidin conjugation kit (abcam, ab102921). The secondary antibody was diluted to 1mg/mL with PBS buffer and conjugated with streptavidin following the instructions in the kit.

(3) Modify the seeds with secondary antibody:

The secondary antibodies conjugated to streptavidin (2AB-STA) were prepared as described in step 2. The DNA nanotube seeds prepared in step 1 were then combined with the 2AB-STA using the solutions given in Supplementary Table S9. The resulting mixture was incubated at room temperature for 1 hour.

Supplementary Table S9: Reagents for modifying nanotube seeds with secondary antibodies

	Final desired concentration	Stock concentration	To add
DNA nanotube seeds (Step 1)	0.3 nM	0.4 nM	75 µl
Secondary antibodies with streptavidin (2AB-STA)	1.8 nM	67 nM	1.61 µl
TAE-Mg^{2+} buffer		1x	23.39 µl
Total			100 µl

(4) Cell preparation and staining of cells with EGFR primary antibody:

In the next step, these antibody-labeled seeds were attached to EGFR primary antibody-labeled HeLa cells.

HeLa cells were prepared and labeled with EGFR primary antibodies as described in Supplementary Note S23 (1)-(4); one well of cells was left unlabeled as a control: only 1% BSA (in DPBS) buffer was added to this well in place of an EGFR primary antibody solution.

(5) Incubation of the secondary antibody modified nanotube seeds with cells:

The secondary antibody-labeled DNA nanotube seeds prepared in step 2 were diluted

to 12 pM using 1% BSA in DPBS buffer with 12mM $MgSO_4$. The solution in each well of live HeLa cells was replaced by 250 µL diluted DNA nanotube seeds solutions prepared above. Both wells of cells were incubated in the refrigerator for 1 hour, which was followed by 3 washes with 250 µL DPBS-12mM $MgSO_4$ buffer.

(6) Fixing of cells and imaging under a spinning disk confocal microscope:

The cells were fixed and imaged under a spinning disk confocal microscope, as described in Supplementary Note S5 steps 6-7.

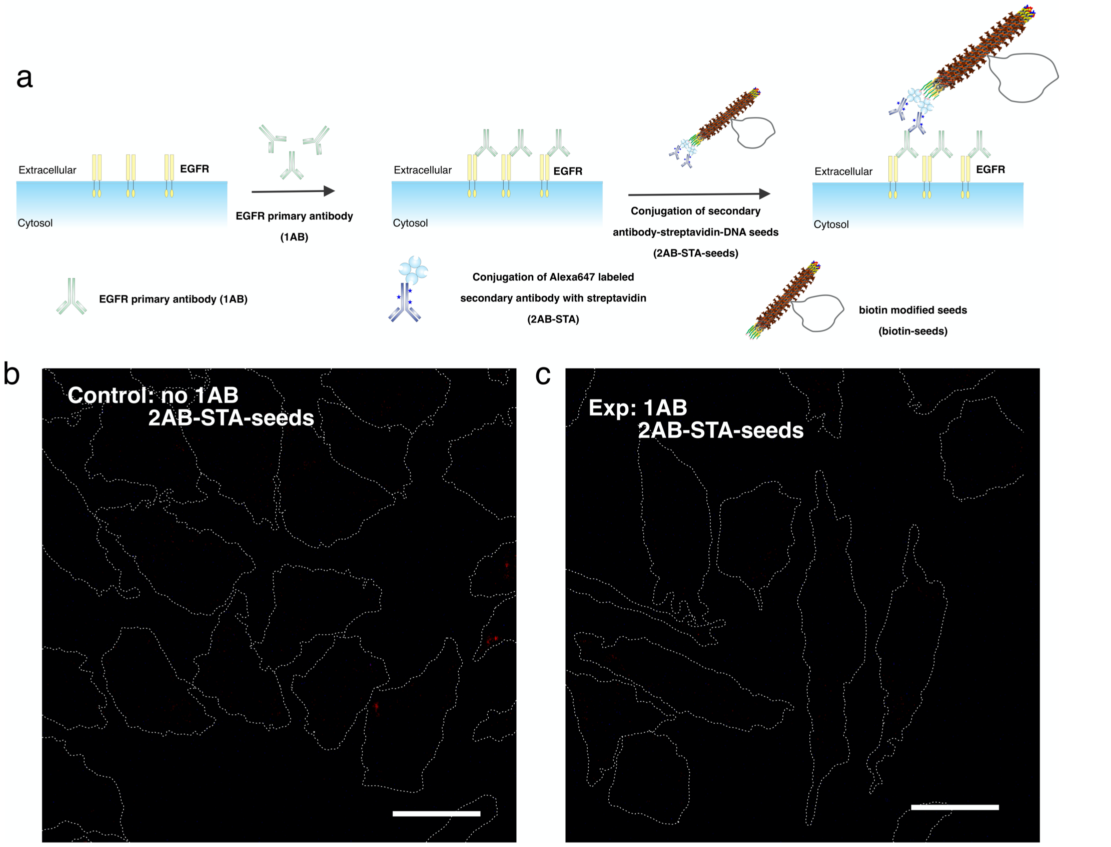

Supplementary Figure S9: Failure to attach secondary antibody-modified seeds to HeLa cell membranes labeled with EGFR antibodies. The experiment was performed as described in Supplementary Note S16. The HeLa cells were fixed before imaging and identified using their autofluorescence. The seeds were labeled with atto488 (red) and the 2AB-STA conjugate was labeled with Alexa647 (blue). Scale bar: 20µm. **a.** Schematic of the attachment process. Cells were labeled with the EGFR primary antibodies (1AB) and the nanotube seeds were modified by attaching secondary antibody-streptavidin (2AB-STA) molecules to biotin groups at the seeds' ends. **b.** Confocal stack micrograph of the control group to which no EGFR primary antibodies were added. **c.** Confocal stack micrograph of the experimental group. There was no observable fluorescence signal from either the nanotube seeds or the 2AB-STA conjugate in either the experimental group or the control group, indicated that no nanotube seeds attached on the cell membrane (specifically or nonspecifically). This experiment was repeated more than three times independently with similar results. The conclusion of this experiment was that the scheme for attaching seeds to cell receptors

in (a) did not work.

Supplementary Note S17: The binding affinity of the BDC sequence to its complement

NUPACK was used to calculate the free energies and equilibrium concentrations of DNA complexes as described in Dirks, *et al.* [14]. In dilute solution, strands A and B can interact to form an ordered complex AB. The ratio of the relative numbers of species in bound and unbound states at equilibrium obeys the relation

$$\frac{x_{AB}}{x_A x_B} = e^{-(\Delta G_{AB}-\Delta G_A-\Delta G_B)/kT}$$

$$= \frac{[AB]/\rho_{H_2O}}{([A]/\rho_{H_2O})([B]/\rho_{H_2O})} \qquad (3.1)$$

$$= \frac{[AB]\rho_{H_2O}}{[A][B]}$$

where for each ordered complex i, x_i is the mole fraction, $[i]$ is the concentration of species in (e.g. in units of Molar), ΔG_i is the free energy of the species i, k is the Boltzmann constant, T is absolute temperature and ρ_{H_2O} ($\approx$ 55.14 mol/L at 37.0°C) is the molar fraction of water.

The dissociation constant K_d is defined as:

$$K_d = \frac{[A][B]}{[AB]} \qquad (3.2)$$

Combining Equations (3.1) and (3.2) yields:

$$K_d = e^{(\Delta G_{AB}-\Delta G_A-\Delta G_B)/kT}\rho_{H_2O} \qquad (3.3)$$

Let A be the BDC tag with sequence AGGAGTGACGATGTG, B the BDC' strand with sequence CACATCGTCACTCCT, and AB the complex consisting of A hybridized to B.

At 37°C (T= 310.15K), NUPACK 3.2.2[15] predicts that for a buffer contains [Na^+] =146mM and [Mg^{2+}] =12.8mM (DMEM-12mM $MgSO_4$), the free energy of ordered complexes for A, B, and AB are:

$$\Delta G^{\circ}_{AB} = -20.06\ kcal/mol$$

$$\Delta G^{\circ}_{A} = -0.28\ kcal/mol$$

$$\Delta G^{\circ}_{B} = -0.15\ kcal/mol$$

Inserting these values and the value of in ρ_{H_2O} into Equation (4.3) yields

$$K_d = 8.1 \times 10^{-13}\ M.$$

Supplementary Note S18: Summary of reagents used for antibody-mediated DNA nanotube anchoring (AMDA) and their incubation processes

AMDA was performed using two types of primary antibodies (EGFR and integrin). The choice of primary antibody directs which receptor nanotube seeds or seeded nanotubes will attach to. AMDA was also performed using multiple cell types (HeLa and HEK293 cells), although the protocol steps for AMDA were similar. Supplementary Table S10 details how each reagent used in AMDA was prepared and how cells were incubated during AMDA.

Supplementary Table S10: Reagents used in AMDA and how cells were incubated with each reagent

Reagent name	Stock concentration	Final concentration of reagent in cell culture	Volume added	Incubation time at 4°C after reagent addition
EGFR primary antibody	200 µg/mL	2 µg/mL (13.3 nM)	2.5 µL	30 min
Integrin β1 primary antibody	200 µg/mL	4 µg/mL (26.7 nM)	2.5 µL	60 min
Alexa-647 labeled secondary antibody-streptavidin conjugate (2AB-STA)	1 mg/mL	10 µg/mL (66.5 nM)	2.5 µL	30 min
Biotinylated secondary antibody	500x	1x	0.5 µL	30 min
Alexa488-labeled streptavidin	2 mg/mL	3 µg/mL (54.5nM)	0.375 µL	30 min
Neutravidin	1 mg/mL	3 µg/mL (50nM)	0.75 µL	30 min
BDC tag	100 µM	1 µM	2.5 µL	30 min
Total			250 µL	

The EGFR primary antibody, *integrin β1 primary antibody*, *biotinylated secondary antibody*, *alexa488-labeled streptavidin* stock were all used as purchased.

The alexa647- labeled secondary antibody-streptavidin conjugate (2AB-STA) stock was prepared as described in step 2 of Supplementary Note S16.

The Neutravidin stock was prepared at 1 mg/mL in PBS buffer.

The BDC tag stock was prepared at 100 µM in water.

Buffers summary:

(1) DMEM: Used as purchased.

(2) Cell growth medium: DMEM containing 10% FBS and 1% penicillin-streptomycin.

(3) Cell growth medium-12mM $MgSO_4$: Cell growth medium with addition of 12mM $MgSO_4$. 1M $MgSO_4$ in water was prepared and then the proper volume of 1M $MgSO_4$ buffer was directly added to the cell growth medium to reach 12mM final concentration right before use.

(4) DMEM-12mM $MgSO_4$: DMEM with addition of 12mM $MgSO_4$. The proper volume of 1M $MgSO_4$ buffer was directly added to the DMEM to reach 12mM final concentration right before use.

(5) 1% BSA (DMEM) buffer: DMEM with addition of 1% BSA (M/V). 10mg BSA was directly added to 1mL DMEM right before use.

(6) 1% BSA (DMEM) -12mM $MgSO_4$ buffer: 1% BSA (DMEM) buffer with addition of 12mM $MgSO_4$. The proper volume of 1M $MgSO_4$ buffer was directly added to the 1% BSA (DMEM) buffer to reach 12mM final concentration right before use.

Supplementary Note S19: Attachment of nanotube seeds with 6 BDC' strands on the seed barrel to HeLa cells using EGFR AMDA

(1) DNA nanotube seeds preparation:

Nanotube seeds with 6 BDC' strands on the ends of their barrels were prepared by combining the reagents in Supplementary Table S11, annealed the mixtures as described in Supplementary Note S1 step 2, purifying the products as in Supplementary Note S1 step 3 and coating the nanotube seeds with PEG as described in Supplementary Note S3. These seeds were labeled with atto488.

The nanotube seeds assembled using this recipe in Supplementary Table S11 had 6 BDC' strands (BDC tag attachment sites) at the right end of each seed's barrel (as illustrated) and were labeled with atto647. Nanotubes grow from the left sides of these seeds.

Supplementary Table S11: 6nt nanotube seeds with 6 BDC' tag attachment sites

Seed Assembly Mixture	Final desired concentration (nM or fold)	Stock concentration (nM or fold)	To add (µl)
H_2O	--	--	72.6 µl
TAE-Mg^{2+} buffer	1x	10x	10 µl
Seed staple strands mix	250nM	4167nM	6 µl
Seed B adapter strands mix (6nt)	100nM	4167nM	2.4 µl
M13mp18 scaffold strand	5nM	100nM	5 µl
biotin right attachment strands mix	20nM	1000nM	2 µl
99Seed labeling attachment strands mix	10nM	1000nM	1 µl
Labeling strand ATTO647	1000nM	100000nM	1 µl
Total			100 µl

(2) Hela cell preparation:

HeLa cells were seeded in 4 wells of an 8-well chamber with 4 x10^4 cells per well overnight as described in step 2 of Supplementary Note S5. The next morning, the chamber was moved into a refrigerator (4°C) for 10 minutes. All buffers used in the following steps (DMEM, 1% BSA (DMEM) buffer, DMEM-12mM $MgSO_4$ and 1% BSA (DMEM) -12mM $MgSO_4$ buffer) and antibody solutions were then put on ice. The chambers were moved out of the refrigerator and placed on ice. They were kept on ice for all following steps when not in the refrigerator for incubation.

(3) Incubation of cells with 1% BSA solution:

The medium in each well containing cells was replaced by 250 µL 1% BSA (DMEM) buffer. The wells with cells were then incubated in a refrigerator (4°C) for 5 minutes.

(4) Staining of live HeLa cells with EGFR primary antibody at 4°C:

10 µL EGFR primary antibody stock at 0.2mg/mL was diluted to 2 µg/mL by adding it to 990 µL cold 1% BSA (DMEM) buffer. After 5 minutes, the solution in each well

containing cells was replaced by 250 µL of the diluted EGFR antibody solution. The cells were then incubated in a refrigerator (4°C) for 30 minutes.

(5) Washing of live HeLa cells on ice:

After 30 minutes of incubation, the wells with cells were placed on ice and each well was washed with cold fresh DMEM 3 times to remove the extra unattached EGFR primary antibody. For each wash cycle, the solution in the well was carefully removed and 250 µL cold fresh DMEM was immediately added with pipette. The cells on ice were gently shaken for 5 minutes after adding fresh DMEM in the second and third wash cycles. The cells in each well were covered by fresh, cold DMEM after wash.

(6) Staining of live HeLa cell with biotinylated secondary antibody at 4°C:

2 µL biotinylated secondary antibody stock was diluted 500-fold by adding it to 998 µL cold 1% BSA (DMEM) buffer. The solution in each well containing cells was removed and 250µl of the diluted biotinylated secondary antibody solution was added immediately. The cells were then incubated in a refrigerator (4°C) for 30 minutes. After incubation, each well containing cells was washed by cold fresh DMEM 3 times in the manner as described in step 5 to remove the extra biotinylated secondary antibodies in the solution.

(7) Staining of live HeLa cell with Alexa 488 labeled streptavidin at 4°C:

3 µL Alexa 488 labeled streptavidin stock at 1mg/mL in PBS buffer was diluted to 3 µg/mL by adding it to 997 µL cold 1% BSA (DMEM) buffer. The solution in each well containing cells was removed and 250µl of this diluted streptavidin solution was added immediately. The cells were then incubated in a refrigerator (4°C) for 30 minutes. After incubation, each well containing cells were washed by cold fresh DMEM 3 times in the manner as described in step 5 to remove the unattached streptavidin in solution.

(8) Coating of live HeLa cells with BDC tag at 4°C:

5 µL BDC tag at 100 µM was diluted to 1 µM by adding it to 495µL cold 1% BSA (DMEM) buffer. The solution in two wells containing cells was removed and 250 µl of this diluted BDC tag was added to each well immediately. The solution in each of the other two wells was replaced by 250 µl cold 1% BSA (DMEM) buffer as control. The cells were then incubated in a refrigerator (4°C) for 30 minutes. After incubation, each well containing cells was washed by cold fresh DMEM 3 times in the manner as described in step 5 to remove the extra BDC tag in the solution.

(9) Attachment of PEG-coated DNA nanotube seeds to live HeLa cells at 4°C:

PEG-coated DNA nanotube seeds were diluted to 16 pM or 64 pM, depending on the desired concentration, by cold 1% BSA (DMEM) -12mM $MgSO_4$ buffer. The solutions in the wells with and without addition of BDC tag were replaced by 250µl of the diluted 16pM nanotube seed solution. The solutions in the other wells with and without addition of BDC tag were replaced by 250µl of the 64pM nanotube seed solution. The 4 wells with cells were then incubated in a refrigerator (4°C) for 30

minutes. After incubation, each well containing cells was washed by cold fresh DMEM-12mM $MgSO_4$ buffer 3 times in the manner described in step 5 to remove the unattached seeds in the solution.

(10) Imaging of the cells under a spinning disk confocal microscope:

Each of the wells of cells were imaged using the spinning disk confocal microscope as described in the step 7 of Supplementary Note S5 and 12 random locations in each well of cells were imaged.

(11) Quantification of the amount of seeds attached to cells:

The methods described in Supplementary Note S6 were used to quantify the amount of seeds attached on cells with changes as described below. The average fluorescence intensity of seeds per cell ($I_{average,S}$) was used to represent the amount of seeds attached to the cell membrane receptor. For each location, one stack image was chosen near the center of the cell to be analyzed to quantify $I_{average,S}$.

First, the total number of cells (N_{cell}) in the chosen stack image at each location was counted manually. As in Supplementary Note S6, we used the visible autofluorescence of cell under 488nm laser to locate the cell positions and count the number of cells.

The total fluorescence intensity of seeds ($I_{total,S}$) at each location was then quantified by processing the chosen stack image of seeds in 488nm channel at each location using the custom MATLAB scripts as described in Supplementary Note S6 with the fluorescence thresholds set at:

$$Threshold_{min,S} = 800 \quad (3.4)$$

$$Threshold_{max,S} = 5000 \quad (3.5)$$

Only pixels with intensities in this range ($800< I_{x,S} < 5000$) were counted. After determining the total fluorescent intensity of seeds ($I_{total,S}$) at each location B, the average fluorescence intensity per cell at each location was calculated using Equation 1.5.

This process was performed on one image from the stacks captured at each of the 12 random locations for each of the samples, i.e. the cells after AMDA with the different seed concentrations (16 pM and 64 pM) and for the control samples where BDC tag was omitted. Error bars for the average fluorescent intensity per cell for each sample represent the standard deviations over the fields of view analyzed (N=12).

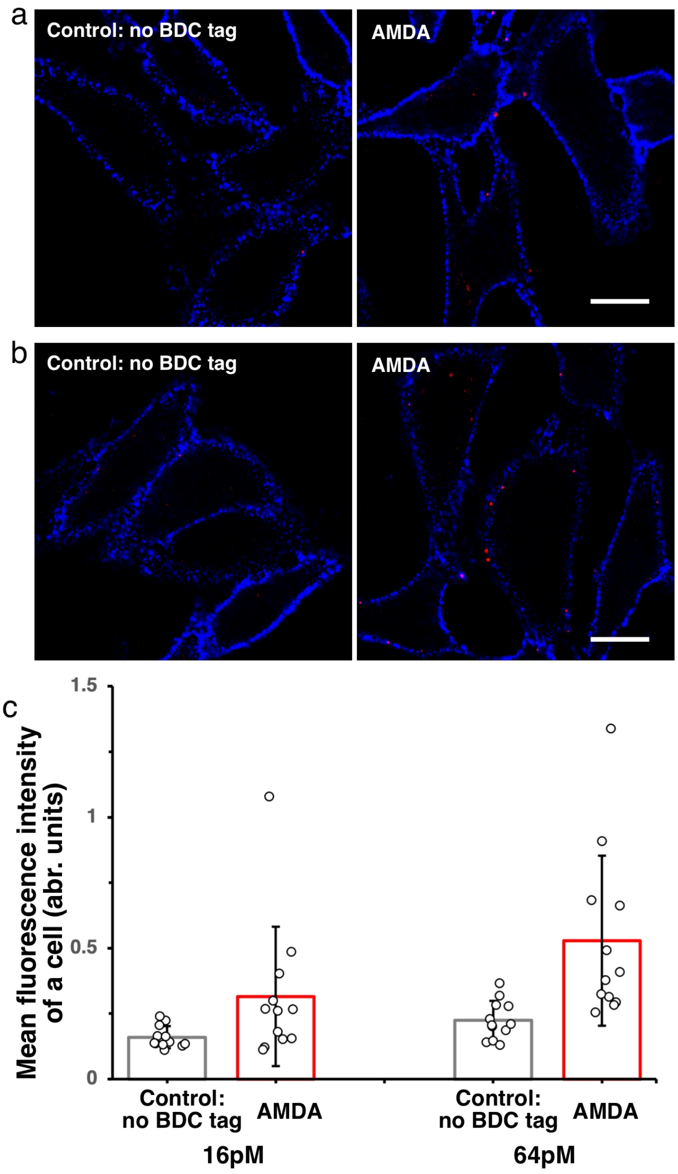

Supplementary Figure S10: HeLa cells after attachment of PEG coated seeds to EGFR receptors via 6 BDC' strands on the seed barrel via AMDA. Experiments were performed as described in Supplementary Note S19. **(a-b)** Confocal stack micrographs of HeLa cells after AMDA or AMDA without the addition of BDC tag as control using PEG coated seeds concentration 16pM (**a**) and 64pM (**b**). Seeds are labeled with Atto647 (red) and neutravidin with Alexa 488 (Blue). Scale bars 20µm. (**c**) Quantification of the average fluorescence intensity per cell after either AMDA and after a control for AMDA processes in which 16 pM (left) or 64 pM (right) of seeds are incubated with cells. The average 647 nm fluorescence intensity of cells after AMDA using 16 pM of seeds was 2.0-fold greater than the average fluorescence intensity of cells after the AMDA control. The average fluorescence intensity of cells after AMDA using 64 pM of seeds was 2.4-fold greater than the average fluorescence intensity of cells after the AMDA control. Error bars are standard deviations over N=12 fields of view analyzed.

Supplementary Note S20: Attachment of nanotube seeds with 6 BDC' strands on the seed barrel and 30 BDC' strands on the scaffold loop to HeLa cells using EGFR AMDA

(1) DNA nanotube seed preparation:

Nanotube seeds with 6 BDC' strands on the ends of their barrels and 30 BDC' strands on the scaffold loop were prepared by combined the reagents in Supplementary Table S12. Seeds for the control, which were prepared without any BDC' strands (without any BDC tag attachment sites), were assembled using the recipe in Supplementary Table S13. Both types of nanotube seeds were then annealed as described in Supplementary Note S1 step 2, purified as in Supplementary Note S1 step 3 and coated with PEG as described in Supplementary Note S3.

Supplementary Table S12: 6nt nanotube seed B with 36 BDC tag attachment sites (atto488 labeled)

Seed Assembly Mixture	Final desired concentration (nM or fold)	Stock concentration (nM or fold)	To add (µl)
H_2O	--	--	71.6 µl
TAE-Mg^{2+} buffer	1x	10x	10 µl
Seed staple strands mix	250nM	4167nM	6 µl
Seed B adapter strands mix (6nt)	100nM	4167nM	2.4 µl
M13mp18 scaffold strand	5nM	100nM	5 µl
Extended biotin right attachment strands mix	20nM	1000nM	2 µl
30 Biotin attachment strands on unused M13 segment	10nM	1000nM	1 µl
66Seed labeling attachment strands mix	10nM	1000nM	1 µl
Labeling strand ATTO488	1000nM	100000nM	1 µl
Total			100 µl

Extended biotin attachment strands mix: A mixture containing all six extended biotin attachment linker strands for seed A (Extended BDC' strand_rightside_01 to Extended BDC' strand_rightside_06) in water each at 1000nM (see Supplementary Note S50.4 Supplementary Figure S49).

30 Biotin attachment strands on unused M13 segment: A mixture containing all 30 biotin attachment strands (BDC' strand on unused_m13mp18_36 to BDC' strand on unused_m13mp18_65) in water each at a concentration of 1000nM (see Supplementary Note S50.4).

66 Seed labeling attachment strands mix: A mixture containing the 66 labeling attachment strands (Unused_m13mp18_01_OLS to Unused_m13mp18_35_OLS and Unused_m13mp18_66_OLS to Unused_m13mp18_96_OLS) in water each at a concentration of 1000nM (see Supplementary Notes S50.2).

The nanotube seeds assembled using the recipe in Supplementary Table S12 had 6 BDC' strands (BDC tag attachment sites) at the right end of each seed's barrel (as illustrated) and 30 BDC' strands (BDC tag attachment sites) in the middle of the unused section of M13 scaffolds (Supplementary Figure S47). These seeds were labeled with atto488 and nanotubes grow from the left sides (as illustrated) of the seeds.

Supplementary Table S13: 6nt nanotube seeds with no BDC tag attachment sites

Seed Assembly Mixture	Final desired concentration (nM or fold)	Stock concentration (nM or fold)	To add (µl)
H_2O	--	--	74.6 µl
TAE-Mg^{2+} buffer	1x	10x	10 µl
Seed staple strands mix	250nM	4167nM	6 µl
Seed B adapter strands mix (6nt)	100nM	4167nM	2.4 µl
M13mp18 scaffold strand	5nM	100nM	5 µl
99Seed labeling attachment strands mix	10nM	1000nM	1 µl
Labeling strand ATTO488	1000nM	100000nM	1 µl
Total			100 µl

The nanotube seeds assembled using the recipe in Supplementary Table S13 had no BDC' strands at the right end of each seed's barrel (as illustrated). These nanotube seeds were labeled with atto488. Nanotubes grow from the left sides (as illustrated) of these seeds.

(2) Hela cell preparation:

HeLa cells were seeded in wells of 8-well chambers with 4 x10^4 cells in 250µL DMEM medium per well overnight as described in step 2 of Supplementary Note S5. The next morning, the chambers were moved into a refrigerator (4°C) for 10 minutes. As in Supplementary Note S28, all the buffers used in the steps below were kept on ice and the cells were also kept on ice when not in the refrigerator.

(3) Incubation of cells with 1% BSA solution:

The medium in each well containing cells was replaced by 250 µL 1% BSA (DMEM) buffer. The cells were then incubated in a refrigerator (4°C) for 5 minutes.

(4) Staining of live HeLa cells with EGFR primary antibodies at 4°C:

EGFR primary antibody stock at 0.2mg/mL was diluted to 2 µg/mL by cold 1% BSA (DMEM) buffer. After 5 minutes, the solution in each well containing cells was removed and 250 µL of diluted EGFR antibody solution was added immediately. The cells were then incubated in a refrigerator (4°C) for 30 minutes.

(5) Washing of live HeLa cells on ice:

After 30 minutes incubation, the wells with cells were moved onto ice and each well was washed with cold fresh DMEM 3 times to remove the extra unattached EGFR

primary antibody. For each wash cycle, the solution in the well was carefully removed and 250 µL cold fresh DMEM was immediately added with pipette. The cell on ice were gently shook for 5 minutes after adding fresh DMEM in the second and third wash cycles. The cells in each well were covered by fresh, cold DMEM after wash.

(6) Staining of live Hela cell with Alexa 647 labeled 2AB-STA conjugate at 4°C:

Alexa 647 labeled secondary antibody-streptavidin conjugate (2AB-STA conjugate), was prepared and quantitated as described in step 2 of Supplementary Note S16 and then was diluted to 10µg/mL by cold 1% BSA (DMEM) buffer. The solution in each well containing cells was removed and 250 µl of this diluted 2AB-STA conjugate solution was added immediately. The cells were then incubated in a refrigerator (4°C) for 30 minutes. After incubation, the wells containing cells were washed with cold fresh DMEM 3 times in the manner as described in step 5 to remove the unattached 2AB-STA conjugate in solution.

(7) Coating of live HeLa cells with BDC tags at 4°C:

100 µM BDC tag solution was diluted to 1 µM by cold 1% BSA (DMEM) buffer. The solution in each well containing cells was removed and 250 µl of this diluted BDC tag solution was added immediately. The cells were then incubated in a refrigerator (4°C) for 30 minutes. After incubation, the wells containing cells were washed by cold fresh DMEM 3 times in the manner as described in step 5 to remove the unattached BDC tag in solution.

(8) Incubation of nanotube seeds with cells:

The seeds with 6 BDC' strands on the ends of their barrels and 30 BDC' strands prepared in step 1 were diluted to the desired concentration (4 pM, 8 pM, 16 pM or 32 pM) by cold 1% BSA(DMEM) -12mM $MgSO_4$ buffer. The solution in each well containing cells was removed and 250 µl of the diluted seed solution with desired concentration was added immediately. Afterwards, the cells were incubated in a refrigerator (4°C) for 30 minutes and washed with cold fresh DMEM-12mM $MgSO_4$ buffer 3 times in the manner described in step 5 to remove the unattached seeds in solution. The cells in each well were covered by fresh, cold DMEM-12mM $MgSO_4$ buffer after wash.

(9) Imaging of the cells under a spinning disk confocal microscope:

Each well containing cells was imaged using the spinning disk confocal microscope as described in the step 7 of Supplementary Note 5. 10 random locations in each well were imaged. For each location, stacks of images were taken from the bottoms of the cell to the tops of the cells with a stack height of 0.27 µm.

(10) Control experiment:

A series of control experiments in which one attachment reagent was omitted were also performed to show the specificity of the AMDA:

For the control experiment in which EGFR primary antibody was eliminated (no 1 AB), instead of adding 250 µL of diluted EGFR antibody solution, 250 µL 1% BSA

(DMEM) buffer was added immediately to the well with cell after the solution in the well been removed in step 4. The other steps were kept the same.

For the control experiment in which the addition of 2AB-STA conjugate was eliminated (no 2AB-STA conjugate), instead of adding 250 μL of diluted 2AB-STA conjugate solution, 250 μL 1% BSA (DMEM) buffer was added immediately to the well with cell after the solution in the well was removed in step 6. The other steps were kept the same.

For the control experiment in which the addition of BDC tag was eliminated (no BDC tag), instead of adding 250 μL of diluted BDC tag solution, 250 μL 1% BSA (DMEM) buffer was added immediately to the well with cell after the solution in the well was removed in step 7. The other steps were kept the same.

For control experiment in which no BDC' strands and biotin attachment sites on the nanotube seeds were added (no BDC' on seeds), the seeds for control prepared in step (1) were diluted to the desired concentration (4 pM, 8 pM, 16 pM and 32 pM) by cold 1% BSA in DMEM-12mM $MgSO_4$ buffer individually. These seeds were added immediately to each well with cells after the DMEM was removed in step 8. The other steps were kept the same.

Supplementary Note S21: Staining of live HeLa cells with DiD dye

The protocol for staining live HeLa cells with DiD dye was adopted from the protocol provided by the company (ThermoFisher).

(1) HeLa cell preparation:

The HeLa cells were seeded in wells of the 8-well chamber with 4 x10^4 cells per well overnight as described in step 2 of Supplementary Note S5.

(2) DiD dye preparation:

Staining medium was prepared by adding 5µL of the DiD dye labeling solution to 1 mL of normal cell growth medium (DMEM medium with 10% FBS and 1% Penicillin-streptomycin), which was a 1:200 dilution.

(3) Staining of live HeLa cell with DiD dye:

The growth medium in each cell wells was removed and 250µL of the diluted DiD solution was added. The cells were put back in the incubator (37°C) and incubated for 8 minutes.

(4) Washing of cells:

After incubation, the staining medium was drained off and the cells in the well were washed by fresh growth medium three times. For each wash cycle, the solution in each well was replaced by 250 µL fresh warmed growth medium and the cells were incubated for 10 minutes in the cell culture incubator. After three cycles of washing, the cells in each well were covered with 250 µL fresh warmed growth medium and at this point are ready for the next step experiment, *i.e.* attaching PEG coated nanotube seeds to suspended HEK293 cell surface through EGFR AMDA (Supplementary Note S20).

Supplementary Note S22: Measuring the number of seeds attached to an average HeLa cell after EGFR AMDA

Here, the average fluorescence intensity of seeds per cell was used to quantify the amount of seeds specifically attached to live HeLa cells using the same methods described in Supplementary Note S6.

As described in Supplementary Note S20, for each of the four seed concentrations, we had prepared 4 control group (No 1AB, no 2AB-STA conjugate, no BDC tag, no BDC' on seeds) and 1 experimental group samples. Z-stack images from 10 locations of each sample were taken under a spinning disk confocal microscope. For each location, one stack image was chosen near the center of the cell to be analyzed to quantify the average fluorescence intensity of seeds per cell.

The total number of cells (N_{cell}) in the chosen stack image at each location was first determined by manual counting. Here the cells were stained by the Alexa 647 labeled secondary antibody-streptavidin conjugates to show the cell outlines clearly in order to count the number of cells in each image described in Supplementary Note S6.

The total fluorescence intensity of seeds ($I_{total,S}$) at each location was then quantified by processing the chosen stack image at each location by custom MATLAB scripts as described in Supplementary Note S6 and by setting the fluorescence thresholds to:

$$Threshold_{min,S} = 750 \quad (3.6)$$

$$Threshold_{max,S} = 5000 \quad (3.7)$$

Only pixels with intensity in this range ($700 < I_{x,S} < 5000$) were counted. After determining the total fluorescence intensities of seeds ($I_{total,S}$) at each location, the average fluorescent intensity per cell at each location was calculated by using Equation 1.5.

This process was performed on one image from the stacks captured at each of the 10 random locations for each of the samples, i.e. the cells after AMDA with the different seed concentrations (4 pM, 8 pM, 16 pM and 32 pM) and for each of the control samples where one of the AMDA components was omitted, *i.e.* no EGFR primary antibody, no 2AB-STA conjugate, no BDC tag, or no BDC' on the seeds. Error bars for the average fluorescent intensity per cell for each sample represent the over the number of fields of view analyzed (N=10).

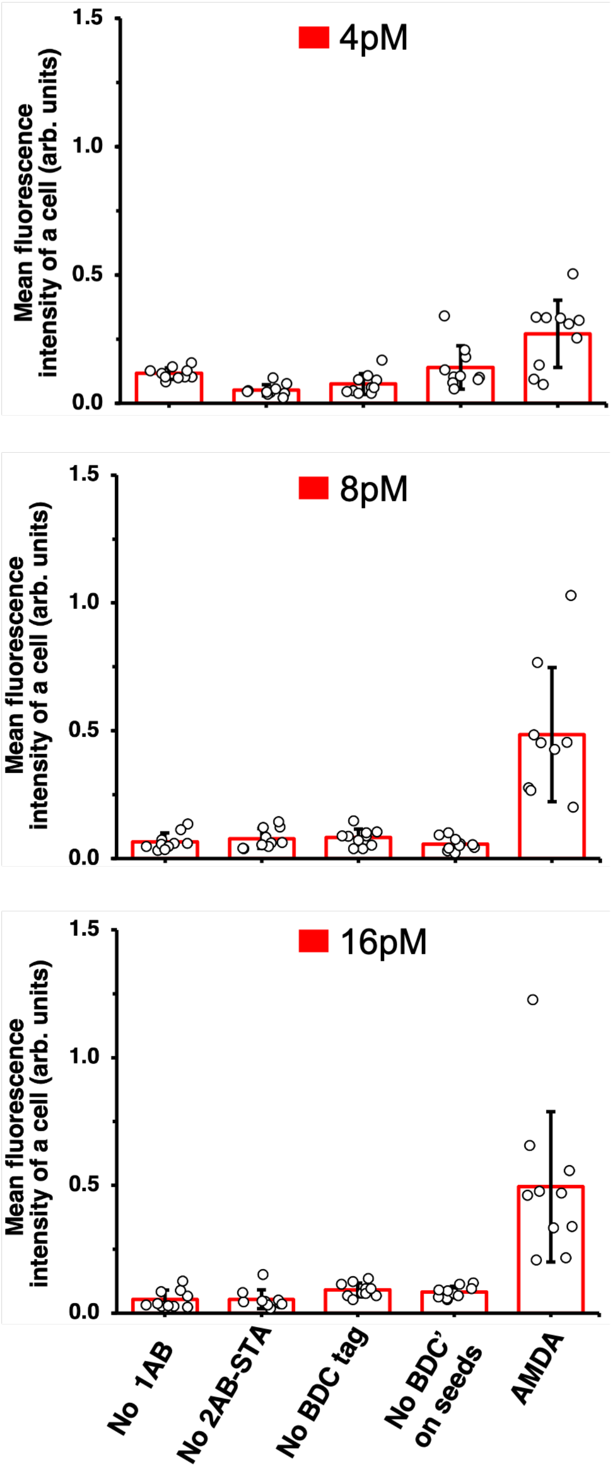

Supplementary Figure S11: The average fluorescence intensity of DNA nanotube seeds per HeLa cell after AMDA with EGFR antibodies using different nanotube seed concentrations. Control experiments each omitted one of the components needed for AMDA, as described by X axis labels. Here, no 1AB: only the addition of EGFR primary antibody was omitted during the AMDA, no 2AB-STA: only the addition of secondary antibody and streptavidin conjugate (2AB-STA) was omitted, no BDC tag: only the addition of the DBC tag was omitted, no BDC' tag on seeds: there

were not BDC' tags modified on nanotube seeds. Error bars are standard deviations of intensity values over the number of fields of view analyzed (N=10 fields of view).

Supplementary Note S23: Attachment of nanotube seeds with 6 BDC' strands on the seed barrel and 30 BDC' strands on the scaffold loop to suspended HEK293 cells

(1) Nanotube seed preparation:

Nanotube seeds with 6 BDC' strands on the ends of their barrels and 30 BDC' strands on their scaffold loops were prepared by combining the reagents in Supplementary Table S12, then annealing as described in Supplementary Note S1 step 2, purifying as described in Supplementary Note S1 step 3 and coating them with PEG as described in Supplementary Note S3. The concentration of the PEG coated nanotube seeds was measured as described in Supplementary Note S1 step 3.

(2) Preparation of suspended HEK293 cells:

HEK293 cells were released from a flask surface using 0.05% Trypsin-EDTA. The concentration of the suspended HEK293 cells was by counted with a hemacytometer and adjusted it to 10^6-10^8 cells per mL before proceeding. For the suspended HEK293 cells, a centrifuge was used for buffer exchange and cell washes in all the steps that follow. As in Supplementary Note S15, all the buffers used in below were kept on ice and the cells were also kept on ice when not in the refrigerator.

(3) Incubation of the suspended HEK293 cells in 1% BSA buffer:

500 μL of suspended HEK293 cells were transferred to a 1.5mL Eppendorf tube and centrifuged at 300xg for 5 minutes. The supernatant was removed by pipetting, being careful to not disturb the cell pellet at the bottom of the tube. The cell pellet was then re-suspended in 500μl of cold 1% BSA (DMEM) buffer. This suspended cell solution was incubated on ice for 5min.

(4) Staining the suspended HEK293 cells with EGFR primary antibodies at 4°C:

5 μL EGFR primary antibody stock at 0.2mg/mL was diluted to 2μg/mL by adding it to 495 μL cold 1% BSA (DMEM) buffer. 500 μL of cells were centrifuged at 300xg for 5 minutes, the supernatant removed carefully by pipetting, and the cells re-suspended in this 500 μL of the diluted EGFR primary antibody solution. The cells were then incubated in a refrigerator (4°C) for 30 minutes with gentle pipetting 5-10 times every 15 minutes. The suspended HEK293 cell can begin to settle in the Eppendorf tube bottom after several minutes. This settling may affect the EGFR primary antibody binding efficiency in solution; gentle pipetting was thus used to ensure the HEK293 cells remained suspended.

(5) Washing the suspended HEK293 cells by centrifugation:

After 30 minutes, the cells were washed by cold DMEM by centrifuging them to remove unattached EGFR primary antibody in the solution. 500 by μL cell were centrifuged at 300xg for 5 minutes and the supernatant was removed carefully by pipetting, then the cell pellet was re-suspended in 1 mL of cold DMEM.

Centrifugation was repeated after which the cells were resuspended in diluted Alexa 647 labeled 2AB-STA conjugate as prepared in next step.

(6) Staining the suspended HEK293 cell with Alexa 647 labeled 2AB-STA conjugate at 4°C:

Alexa 647-labeled secondary antibody-streptavidin conjugate (2AB-STA conjugate) was prepared and quantitated as described in step 2 of Supplementary Note S16. After the final centrifugation procedure in step 5, 5µL Alexa 647 labeled 2AB-STA conjugate stock at 1mg/mL was diluted to 10 µg/mL by adding it to 495 µL cold 1% BSA (DMEM) buffer. After the centrifugation finished and the supernatant was removed, the cell pellet was immediately resuspended in this 500 µL diluted Alexa 647 labeled 2AB-STA conjugate solution and incubated in a refrigerator (4°C) for 30 minutes with gentle pipetting every 15 minutes prevent the cells from settling to the bottom of the tube.

After 30 minutes, the cells were washed by DMEM by centrifuging them to remove unattached Alexa 647-labeled 2AB-STA conjugate. 500 µL of cells were centrifuged at 300xg for 5 minutes and the supernatant was removed carefully by pipetting. The cell pellet was then re-suspended in 1 mL of cold DMEM. This 1 mL of cell solution was aliquoted into two tubes with 500µL per tube and the each tube was centrifuged at 300xg for 5 minutes. After centrifuging, the cell pellet in one tube was re-suspended in BDC tag solution while the other was suspended in buffer without BDC tag as a control in the next step 7.

(7) Coating the suspended HEK293 cells with BDC tag at 4°C:

During the centrifugation in step 6, 2.5 µL of the BDC tag at 100 µM was diluted to 1 µM by adding it to 247.5 µL cold 1% BSA (DMEM) buffer. Once the centrifugation had finished and the supernatant was removed, the cell pellet in one of the tubes was immediately re-suspended in 250µL of the diluted BDC tag solution, while the other tube of cell pellet in 250 µL of 1% BSA (DMEM) buffer, as a control. Both tubes of cells were incubated in a refrigerator (4°C) for 30 minutes with gentle pipetting 5-10 times every 15 minutes. After 30 minutes, both tubes of cells were washed with cold DMEM via a centrifuge wash as described in step 5.

(8) Incubation of nanotube seeds with suspended HEK293 cell at 4°C:

During the centrifugation in step 7, the PEG-coated DNA nanotube seeds with 36 biotin attachment sites from step 1 were diluted to the concentration of 48pM by cold 1% BSA (DMEM) -12mM $MgSO_4$ buffer. Once the centrifugation finished and the supernatant was removed, the cell pellet in both tubes were immediately re-suspended in 250 µL of diluted nanotube seed solution and incubated in a refrigerator (4°C) for 30 minutes with gentle pipetting 5-10 times every 15 minutes. After the incubation, both tubes of cells were washed with cold DMEM-12.5mM $MgSO_4$ buffer via a centrifugation wash as described in step 5 to remove the extra seeds not attached to cells.

(9) Imaging of suspended HEK293 cells under a spinning disk confocal microscope:

250 μL of suspended HEK 293 cells prepared in step 8 was added to one well of the 8-well chambers. They were left for several minutes to allow the cells settle down on the bottom of well. If there were too many cells for imaging, DMEM-12.5mM $MgSO_4$ buffer was added to the well. A spinning disk confocal microscope was used to take images of HEK cells with attached nanotube seeds at random locations in each well as described in Supplementary Note S5 step 7. At each location, stacks of images were taken from the bottoms of the cells to the tops of the cells with a stack height of 0.5 μm.

Supplementary Note S24: Quantification of the number of nanotube seeds attached to suspended HEK293 cells by EGFR AMDA using flow cytometry

We used two methods to measure the average fluorescent intensity of seeds per cell as a measure of the number of nanotube seeds attached to the suspended HEK293 cell: (1) using confocal images to measure the average fluorescent intensity of seeds per HEK293 cell, (2) using flow cytometry to measure the average fluorescence intensity of the seeds on each HEK293 cell.

24.1 Measurement of the average fluorescent intensity of seeds per cell using confocal images:

Here, the average fluorescence intensity of seeds per cell was used to quantify the number of seeds specifically attached to suspended HEK293 cells using the same methods described in Supplementary Note S6.

We prepared the control group (no BDC tag) and the experimental group of cells as described in Supplementary Note S23. Z-stack images from 8 locations in each sample were taken using a spinning disk confocal microscope. For each location, one stack image was chosen near the center of the cell to be analyzed to quantify the average fluorescence intensity of seeds per cell.

The total number of cells (N_{cell}) in the chosen stack image at each location was first counted manually. The cells were stained with Alexa 647 labeled 2AB-STA conjugate to clearly show the cells' outlines in the 647nm channel. These outlines were used to count the number of cells as described in Supplementary Note S6.

The total fluorescence intensity of seeds ($I_{total,S}$) at each location was then quantified by processing the chosen stack image of seeds in the 488nm channel at each location using custom MATLAB scripts, as described in Supplementary Note S6. Here the fluorescence thresholds were set as:

$$Threshold_{min,S} = 2500 \qquad (3.8)$$

$$Threshold_{max,S} = 8000. \qquad (3.9)$$

Only pixels with intensities in this range ($2500 < I_{x,S} < 8000$) were counted. The average fluorescence intensity of seeds per cell at each location was then calculated using Equation 1.5.

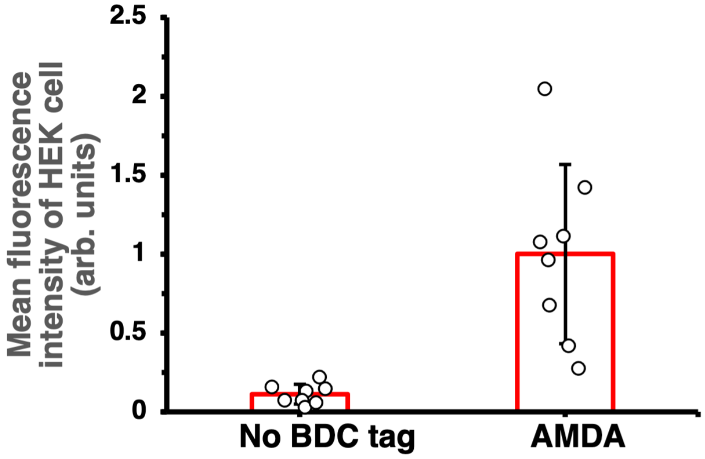

Supplementary Figure S12: Average fluorescence intensities of nanotube seeds per HEK293 cell after AMDA or after AMDA omitting BDC tag with 48pM seeded added. Nanotube seeds were labelled with Atto488 and secondary antibody-streptavidin conjugate with Alexa647. Experiments were performed as described in Supplementary Note S23. The fluorescence intensity was 9-fold higher after seeds were attached by AMDA than after a control process. Error bars are standard deviations of intensity values per field of view (N=8 fields of view).

24.2 Measurement of the average fluorescent intensity of seeds per cell by flow cytometry:

Flow cytometry was used to quantify the number of DNA nanotube seeds attached to suspended HEK293 cell after EGFR AMDA. Three samples were prepared for the measurement: an experimental group sample for which all the AMDA reagents were added to specifically attached nanotube seeds to the surfaces of suspended HEK293 cells, a control group sample for which EGFR AMDA was performed excepted for the addition of the BDC tag (No BDC tag) and a blank sample that contained HEK293 cells that had not undergone AMDA (no AMDA steps).

(1) Nanotube seeds preparation:

PEG coated nanotube seeds were prepared as described in Supplementary Note S23 step 1.

(2) Suspended HEK293 cell preparation:

Suspended HEK293 cells were prepared as described in Supplementary Note S31 step 2 to a concentration between 10^6-10^8 cells per mL. 200 µL of the suspended HEK cell solution was transferred to a 1.5mM Eppendorf tube as the blank sample and stored in refrigerator (4°C).

(3) Incubation of suspended HEK293 cells in 1% BSA buffer:

Another 500 µL of suspended HEK293 cells were exchanged into cold 1% BSA

(DMEM) buffer by centrifugation as described in Supplementary Note S23 step 3 and incubated on ice for 5 minutes.

(4) Staining of the suspended HEK293 cells with EGFR primary antibody at 4°C:

The suspended HEK293 cell were stained with EGFR primary antibody as described in Supplementary Note S23 step 4 and washed with cold DMEM *via* centrifugation as described in Supplementary Note S23 step 5 to remove the unattached EGFR primary antibodies.

(5) Staining of the suspended HEK293 cell with biotinylated secondary antibodies at 4°C:

During the centrifugation in step 4, 1 μL biotinylated secondary antibody stock was diluted 500 fold by adding it to 499 μL cold 1% BSA in DMEM. Once the centrifugation finished and the supernatant was removed, the cell pellet was immediately re-suspended in this diluted biotinylated secondary antibody solution and then incubated in a refrigerator (4°C) for 30 minutes with gentle pipetting 5-10 times every 15 minutes. After 30 minutes, the cells were washed with cold DMEM *via* centrifugation as described in Supplementary Note S23 step 5 to remove unattached biotinylated secondary antibodies.

(6) Staining of suspended HEK293 cells with neutravidin at 4°C:

During the centrifugation of step 5, 1.5 μL of the neutravidin stock at 1mg/mL was diluted to 3μg/mL by adding it to 498.5 μL cold 1% BSA (DMEM) buffer. Once the centrifugation finished and the supernatant was removed, the cell pellet was immediately re-suspended in this diluted neutravidin solution and then incubated in a refrigerator (4°C) for 30 minutes with gentle pipetting 5-10 times every 15 minutes. After 30 minutes, the cells were washed by cold DMEM *via* centrifugation and aliquoted into two tubes as described in Supplementary Note S23 step 6.

(7) Coating of the suspended HEK293 cells with BDC tag at 4°C:

One tube of suspended HEK293 cell was coated with BDC tag as described in Supplementary Note S23 step 7 and the other tube was prepared as a control: no BDC tag (BDC tag-) was added.

(8) Incubate nanotube seeds with suspended HEK293 cell at 4°C:

The PEG-coated DNA nanotube seeds with 36 biotin attachment sites were added to both the experimental and control samples as described in Supplementary Note S23 step 8.

(9) Flow cytometry

For each cell sample, the cell suspension was transferred to tubes from which cells are sucked into the flow cytometer. ~10000 cells were analyzed using a FACSCanto flow cytometer (BD Biosciences, USA) with BD FACSDiva software v8.0. Cells were excited using a 488 nm and fluorescence emission were collected using a GFP filter to measure the fluorescence of the seeds. The forward scatter (FSC, light scatter along the path of the laser) and the side scatter (SSC, light scatter at a night degree angle

relative to the laser) were also collected. The FSC allows for discrimination of cells by size and SSC provided information about the internal complexity (i.e. granularity) of the cells. Based on the FSC and SCC, the fluorescence signals of cells with proper size and granularity were selected to analyze by software FlowJo 10.4.

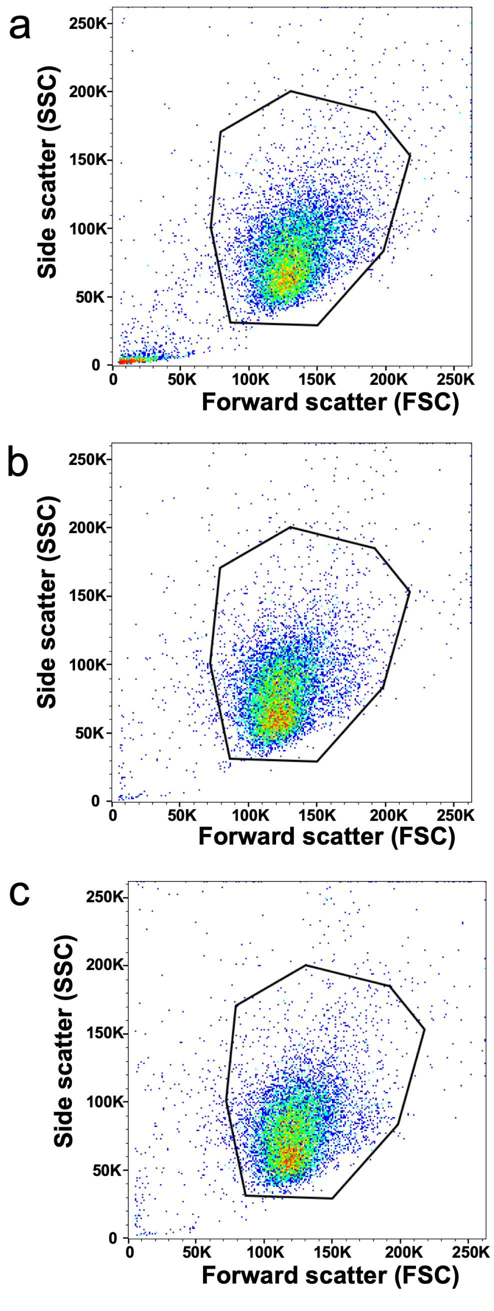

Supplementary Figure S13: Measurement of forward and side scatter of suspended HEK293 cells with nanotube seeds attached using EGFR AMDA during flow cytometry. Cells were prepared and flow cytometry measurements were performed as described in Supplementary Note S24. The nanotube seeds were labeled with atto488. The HEK293 cells were otherwise unlabeled. Flow cytometry analysis of the blank sample (no AMDA steps) (**a**) (84.1% of cells gated), the control sample (all AMDA steps except the addition of the BDC tag) (**b**) (95.6% of cells gated) and the experimental group sample (all AMDA steps) (**c**) (94.8% of cells

gated). Based on cell size and granularity (forward and side scatter), the fluorescence signals of the cells inside the black polygons were selected to analyze.

Supplementary Note S25: Measuring the correlation in locations of nanotube seeds and antibodies on cell membranes using confocal micrographs

To determine the extent of correlation in the locations of the attached nanotube seeds and EGFR receptors, we repeated the AMDA process on HeLa cells using EGFR antibodies but by using many fewer antibodies to allow each attached side to be precisely resolved with respect to others. Specifically, the procedure for AMDA on HeLa cells in Supplementary Note S20 was followed except that the EGFR primary antibodies were diluted to a concentration 0.05 µg/mL by cold 1% BSA (DMEM) buffer for use in step 4. This solution was 40-fold more dilute than the solution used in Supplementary Note S20. The seeds with 6 BDC' strands on the ends of their barrels and 30 BDC' strands prepared in step 1 were diluted to 32 pM by cold 1% BSA(DMEM) -12mM $MgSO_4$ buffer in step 8 here.

To determine the binding fraction of nanotube seeds with antibodies in our fluorescence microscopy images, we employed an image processing script utilizing algorithms from the Python library scikit-image[16]. First, edge detection was performed using the Canny algorithm[17] to trace an outline of each cell membrane. Small gaps in the detected edges were closed by applying a dilation (to join adjacent edges) followed by an erosion (to restore a single pixel-width edge). For the purposes of our analysis, all points within 10 pixels of this edge are defined to be part of the cell membrane. Next, we detected both nanotube seeds and antibodies using the Laplacian of Gaussians blob detection algorithm. The list of nanotube seed and antibody coordinates was then culled to remove seeds and antibodies that were not within our definition of the cell membrane. Finally, for each nanotube seed on the culled list, we calculated the distance to each antibody. If a seed had an antibody within 5 pixels of it, we counted it as "bound." If no antibody was within 5 pixels of the nanotube seed, we counted the seed as "unbound." The number of bound seeds divided by the total number of seeds on the culled list represents the experimentally measured binding fraction.

To determine whether the proximity of seeds and antibodies was due to physical interaction and not simply the result of random overlap between the two, we simulated what the binding fraction would be if the nanotube seeds were positioned randomly within our definition of the membrane. To do this, we randomly selected a number of points within the membrane equal to the number of seeds detected and calculated the seed binding fraction using those points as seeds, rather than the actual position of the seeds. We repeated this process 1000 times in order to estimate the "null" binding fraction that would result from simple overlap.

Supplementary Note S26: Attachment of seeded nanotubes to EGFR receptors on HeLa cells using the EGFR AMDA protocol

(1) Seeded nanotube preparation:

PEG coated nanotube seeds with 6 BDC' strands on the ends of their barrels and 30 BDC' strands on each scaffold loop were prepared by combining the reagents in Supplementary Table S14, then annealing as described in Supplementary Note S1 step 2, purifying the seeds as in Supplementary Note S1 step 3 and coating them with PEG as described in Supplementary Note S3. The concentrations of PEG coated nanotube seeds were determined as described in Supplementary Note S1 step 3 and adjusted to 0.4nM by diluting them with 1x TAE-Mg^{2+} buffer.

The nanotube seeds assembled by the recipe in Supplementary Table S14 had 6 BDC' strands (BDC tag attachment sites) at the right end (as illustrated) of each seed's barrel and 30 BDC' strands (BDC tag attachment sites) in the middle of the unused section of M13 scaffolds (Supplementary Figure S47). These seeds were labeled with atto647 and nanotubes grow from the left sides (as illustrated) of the seeds.

Supplementary Table S14: 6nt nanotube seed B with 36 BDC tag attachment sites (atto647 labeled)

Seed Assembly Mixture	Final desired concentration (nM or fold)	Stock concentration (nM or fold)	To add (µl)
H_2O	--	--	71.6 µl
TAE-Mg^{2+} buffer	1x	10x	10 µl
Seed staple strands mix	250nM	4167nM	6 µl
Seed B adapter strands mix (6nt)	100nM	4167nM	2.4 µl
M13mp18 scaffold strand	5nM	100nM	5 µl
Extended biotin right attachment strands mix	20nM	1000nM	2 µl
30 Biotin attachment strands on unused M13 segment	10nM	1000nM	1 µl
66Seed labeling attachment strands mix	10nM	1000nM	1 µl
Labeling strand ATTO647	1000nM	100000nM	1 µl
Total			100 µl

Using these nanotube seeds, the PEG coated seeded nanotubes were assembled by the method described in Supplementary Note S7.2. 14 aliquots of 19.7 µL monomers were prepared as described in Supplementary Note 7.2 (Supplementary Table S5). When the temperature reached 37°C, 2µL PEG coated seeds prepared as described above (0.4nM) were added to each aliquot and all of the samples were incubated at 37°C for 3 days.

(2) HeLa cell preparation:

The HeLa cells were seeded in 2 wells of an 8-well chambers with 4 x10^4 cells

overnight as described in step 2 of Supplementary Note S5. The next morning, the cell were moved into a refrigerator (4°C) for 10 minutes. As in Supplementary Note S15, all the buffers and AMDA reagents (all antibodies, Alexa 488 labeled streptavidin, and BDC tags) and stocks used in the following steps were kept on ice and the cells were also kept on ice when they were not in the refrigerator.

(3) Incubation of the cells with 1% BSA solution:

The medium in each well containing cells was replaced by 250 µL 1% BSA (DMEM) buffer. The cells were then incubated in a refrigerator (4°C) for 5 minutes.

(4) Staining the live cells with EGFR primary antibodies at 4°C:

5 µL of EGFR primary antibody stock solution (0.2 mg/mL) was diluted to 2 µg/mL by adding it to 495µL cold 1% BSA (DMEM) buffer. After 5 minutes, the solution in each well containing cells was removed and 250 µL of diluted EGFR antibody solution was added to each well immediately. The cells were then incubated in a refrigerator (4°C) for 30 minutes.

(5) Washing live HeLa cells on ice:

After 30 minutes of incubation, the wells with cell were moved onto ice and each well was washed with cold fresh DMEM 3 times to remove the extra unattached EGFR primary antibody. For each wash cycle, the solution in the well was carefully removed and 250 µL cold fresh DMEM was immediately added with pipette. The cells were gently shaken while on ice for 5 minutes after the second and third wash cycles. The cells in each well were covered by fresh, cold DMEM after the washes.

(6) Staining of live cells with biotinylated secondary antibodies at 4°C:

1µL of the biotinylated secondary antibody stock was diluted 500-fold by adding it to 499 µL of cold 1% BSA (DMEM) buffer. The solution in each well containing cells was removed and 250 µl of this diluted biotinylated secondary antibody solution was added to each well immediately. The cells were then incubated in a refrigerator (4°C) for 30 minutes. After incubation, the wells containing cells were washed by cold fresh DMEM 3 times in the manner as described in step 5 to remove the unattached biotinylated secondary antibody in solution.

(6) Staining of live cells with Alexa 488 labeled streptavidin at 4°C:

0.75 µL of the Alexa 488 labeled streptavidin stock (2 mg/mL) was diluted to 3 µg/mL by adding it to 499.25µL cold 1% BSA (DMEM) buffer. The solution in each well containing cells was removed and 250 µl of the diluted Alexa 488 labeled streptavidin solution was added to each well immediately. The cells were then incubated in a refrigerator (4°C) for 30 minutes. After incubation, the wells containing cells were washed with cold fresh DMEM 3 times in the manner as described in step 5 to remove the unattached Alexa 488 labeled streptavidin in solution.

(7) Coating of live HeLa cell with BDC tags at 4°C:

2.5µL of the BDC tag sock (100 µM) was diluted to 1 µM by adding it to 247.5µL cold 1% BSA (DMEM) buffer. The solution in one well containing cells was replaced

by 250 µL of the diluted BDC tag solution, while the solution in the other well was replaced by 250 µL of cold 1% BSA (DMEM) buffer, as a control. The cells were then incubated in a refrigerator (4°C) for 30 minutes. After incubation, the wells containing cells were washed with cold fresh DMEM 3 times in the manner as described in step 5.

(8) Incubation of seeded nanotubes with cells:

250 µL of the PEG-coated DNA seeded nanotubes prepared in step 1 were diluted 1-fold by adding it to 250 µL cold 1% BSA in DMEM-12mM $MgSO_4$ buffer. The solution in each well containing cells was removed and 250µL of the diluted seeded nanotube solution was added to each well immediately. The cells were incubated in a refrigerator (4 °C) for 2 hours. After incubation, the wells containing cells were washed with cold fresh DMEM-12mM $MgSO_4$ buffer 3 times in the manner as described in step 5 to remove the unattached seeded nanotubes in solution.

(9) Imaging of the cells under a spinning disk confocal microscope:

Each of the wells containing cells was imaged using a spinning disk confocal microscope as described in the step 7 of Supplementary Note 5. 10 random locations in each well were imaged. For each location, stacks of images were taken from the bottoms of the cell to the tops of the cells with a stack height of 0.27 µm. Time lapse video was also captured at one height at a rate of 2 seconds per frame.

Supplementary Note S27: Quantifying the number of seeded nanotubes attached to the HeLa cell membranes

The average fluorescence intensity of seeds per cell and the average fluorescence intensity of nanotubes per cell were each used to quantify the amount of seeded nanotube specifically attached to live HeLa cell using the same methods described in Supplementary Note S6.

We prepared the control group (no BDC tag) and the experimental group as described in Supplementary Note S26. Z-stack images from 10 locations in each sample were taken using a spinning disk confocal microscope. For each location, one stack image was chosen near the center of the cell to be analyzed to quantify the average fluorescence intensity of seeds per cell.

The total number of cells (N_{cell}) in the chosen stack image at each location was first counted manually. The cells were stained with antibodies and Alexa 488 labeled streptavidin to clearly show the cells' outlines in the 488nm channel. These outlines were used to count the number of cells as described in Supplementary Note S6.

The total fluorescence intensity of seeds ($I_{total,S}$) at each location was then quantified by processing the chosen stack image of seeds in the 647nm channel at each location using custom MATLAB scripts, as described in Supplementary Note S6. Here the fluorescence thresholds were set as:

$$Threshold_{min,S} = 800 \tag{3.10}$$

$$Threshold_{max,S} = 5000. \tag{3.11}$$

Only pixels with intensities in this range ($800< I_{x,S} < 5000$) were counted. The average fluorescence intensity of seeds per cell at each location was then calculated using Equation 1.5.

The total fluorescence intensity of nanotube ($I_{total,NT}$) at each location was then quantified by processing the chosen stack image of seeds in the Cy3 channel at each location using custom MATLAB scripts as described in Supplementary Note S6. Here the fluorescence thresholds were set as:

$$Threshold_{min,NT} = 1050 \tag{3.12}$$

$$Threshold_{max,NT} = 5000. \tag{3.13}$$

The $Threshold_{min,NT}$ value was determined by looking at the intensities of areas within the background without nanotubes. The $Threshold_{max,NT}$ value was determined by looking at the intensities of areas containing aggregated nanotubes. Only pixels with intensity between these values were counted.

For each pixel x, its intensity I_x included the background and the fluorescence of nanotube:

$$I_x = I_{x,NT} + Threshold_{min,NT} \quad (3.14)$$

Where $I_{x,NT}$ is the nanotube fluorescence intensity at pixel x.

The total fluorescence intensity of nanotubes of the chosen stack image for each location was calculated by summing the nanotube fluorescence intensities of all pixels:

$$\begin{aligned} I_{total,NT} &= \sum I_{x,NT} \\ &= \sum (I_x - Threshold_{min,NT}) \end{aligned} \quad (3.15)$$

For each location, the chosen stack image was processed using MATLAB script as described above to get $I_{total,NT}$. The average fluorescent intensity of nanotubes per cell at each location was then:

$$I_{average,NT} = \frac{I_{total,NT}}{N_{cell}} \quad (3.16)$$

This process was performed on one image from the stacks captured at each of the 10 random locations for both the control and experiment group. Error bars for the average fluorescent intensity per cell for each sample represent the 95% confidence intervals of proportions.

Supplementary Note S28: Attachment of seeded nanotubes to EGFR receptors on suspended HEK293 cells using EGFR AMDA

PEG coated seeded nanotubes were assembled as described in Supplementary Note S26 step 1. The suspended HEK293 cells were prepared and stained with EGFR primary antibody and biotinylated secondary antibody as described in Supplementary Note S24 step 2-5. Then the suspended HEK293 cell were stained with Alexa 488 labeled streptavidin instead of neutravidin: 0.75 µL Alexa 488 labeled streptavidin stock (2 mg/mL) was diluted to 3µg/mL by adding it to 499.25 µL cold 1% BSA (DMEM) buffer. The cell pellet was then re-suspended in this diluted Alexa 488 labeled streptavidin solution, after which time the cells were incubated in a refrigerator (4°C) for 30 minutes with gentle pipetting 5-10 times every 15 minutes. After 30 minutes, the cells were washed with cold DMEM *via* centrifugation and aliquoted into two tubes as described in Supplementary Note S23 step 6. The suspended HEK293 cells in one tube were coated with BDC tags while the HEK293 cells in the other tube were prepared as controls with no addition of BDC tags (BDC tag-), as described in Supplementary Note S23 step 7. Then we incubated the nanotube solution with the suspended HEK293 cells as follows:

The prepared PEG coated DNA seeded nanotubes were diluted 1-fold in cold 1% BSA in DMEM-12mM $MgSO_4$ buffer. Both tubes of cells from above were centrifuged and re-suspended in 250 µL of the diluted seeded nanotube solution. The cells were then incubated on ice for 2 hours and pipetted gently every 15 minutes during the incubation period. After incubation, the cells were washed by centrifuging and re-suspending them in 250 µL of cold DMEM-12.5mM $MgSO_4$ buffer. The seeded nanotubes anchored on the suspended HEK293 cells were imaged using a spinning disk confocal microscope as described in Supplementary Note S23, steps 9; random positions were imaged within each well. At each location, stacks of images were taken from the bottoms of the cell to the tops of the cells with a stack height of 0.5 µm.

Supplementary Note S29: Quantifying the number of seeded nanotubes attached to suspended HEK293 cells

Here we used two methods to quantify the amount of seeded nanotubes attached to suspended HEK293 cell membrane: (1) use the average fluorescent intensity of seeds per cell as a measure of the amount of seeded nanotubes nonspecifically attached to a cell membrane, (2) use the average number of seeds per cell as a measure of the amount of seeds nonspecifically attached to cell membreane.

29.1 Measurement of the average fluorescent intensity of seeded nanotubes on a cell

The average fluorescence intensity of seeds per cell and the average fluorescence intensity of nanotubes per cell were each used to quantify the amount of seeded nanotube specifically attached to suspended HEK293 cell by EGFR AMDA using the same methods described in Supplementary Note S27.

We prepared the control group (no BDC tag) and the experimental group as described

in Supplementary Note S28. Z-stack images from 8 locations in each sample were taken using a spinning disk confocal microscope. For each location, one stack image was chosen near the center of the cell to be analyzed to quantify the average fluorescence intensity of seeds/nanotubes per cell.

The total number of cells (N_{cell}) in the chosen stack image at each location was first counted manually as described in Supplementary Note 27.

The total fluorescence intensity of seeds ($I_{total,S}$) at each location was then quantified by processing the chosen stack image of seeds in the 647nm channel at each location using custom MATLAB scripts, as described in Supplementary Note S6. Here the fluorescence thresholds were set as:

$$Threshold_{min,S} = 1250 \quad (3.17)$$

$$Threshold_{max,S} = 5000. \quad (3.18)$$

Only pixels with intensities in this range ($1250 < I_{x,S} < 5000$) were counted. The average fluorescence intensity of seeds per cell at each location was then calculated using Equation 1.5.

The total fluorescence intensity of nanotube ($I_{total,NT}$) at each location was then quantified by processing the chosen stack image of seeds in the Cy3 channel at each location using custom MATLAB scripts as described in Supplementary Note S6. Here the fluorescence thresholds were set as:

$$Threshold_{min,NT} = 2200 \quad (3.19)$$

$$Threshold_{max,NT} = 5000. \quad (3.20)$$

Only pixels with intensities in this range ($2200 < I_{x,S} < 5000$) were counted. The average fluorescence intensity of nanotube per cell at each location was then calculated using Equation 3.16.

This process was performed on one image from the stacks captured at each of the 8 random locations for both the control and experiment group. Error bars for the average fluorescent intensity per cell for each sample represent standard deviations of intensity values per field of view (N=8 fields of view).

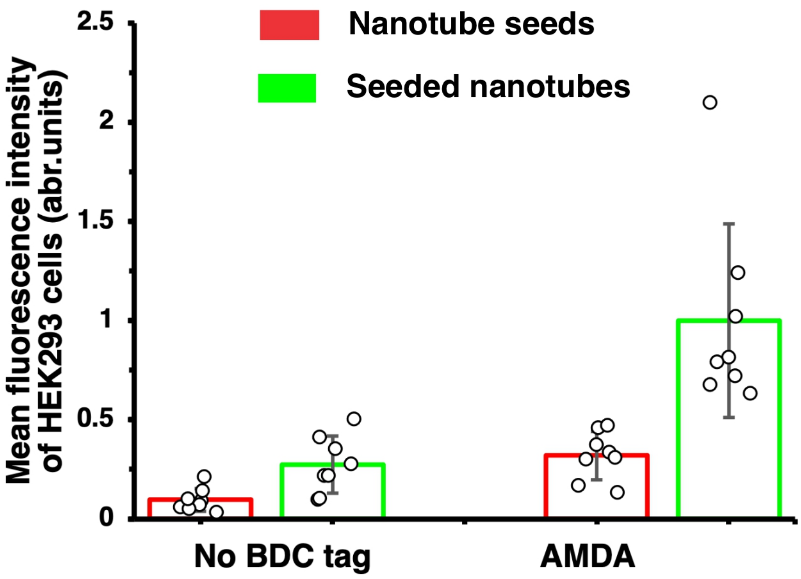

Supplementary Figure S14: The average fluorescence intensities of HeLa cells with seeded nanotubes or seeds attached via AMDA and AMDA with BDC tag addition omitted (Supplementary Note S28). Error bars are standard deviations of intensity values per field of view (N=8 fields of view).

29.2 Measurement of the average number of seeded nanotubes per cell

The average number of seeded nanotubes per cell was used to quantify the number of seeded nanotubes attached to the suspended HEK293 cells. The stacks were imaged from cell bottom to cell top at ten random locations on one slide. A 3-dimensional projection image was then generated from the stack of images collected at each location. The total number of both cells and the total number of seeded nanotubes attached to cells were each counted in this projection image. The average number of seeded nanotubes per HEK293 cell was then calculated by dividing the total number of seeded nanotubes divided by the total number of cells. This quantification method was applied to the stack images from 10 random locations.

Supplementary Note S30: Attachment of nanotube seeds and seeded nanotubes to integrin receptors on HeLa cells using integrin AMDA

The Integrin β1 antibody was used to attach the nanotube seeds and seeded nanotubes to the HeLa cell membranes. The procedure for Integrin AMDA, described below, was almost the same as for EGFR AMDA described in Supplementary Note 26.

(1) Seeded nanotube preparation:

PEG coated nanotube seeds were prepared as described in Supplementary Note S20 step 1. PEG coated seeded nanotubes were prepared as described in Supplementary Note S26 step 1.

(2) GFP labeled HeLa cell preparation:

Instead of using HeLa cell in step 2, the GFP labeled HeLa cell were used. To generate HeLa cells stably transduced with GFP, we used the plasmid backbone pLVTHM (Addgene; plasmid#12247). The non-specific scramble sequence (5'-GCACTACCAGAGCTAACTCAGATAGTACT-3') was subcloned into the backbone using MluI and ClaI as restriction sites. To produce lentivirus, 293T/17 cells were co-transfected with psPAX2, pMD2.G and the lentiviral plasmid. Lentivirus was harvested 48h after transfection and was concentrated via centrifugation (50,000 g for 2 hours at 4°C). Subsequently a 100X virus suspension was incubated overnight with 60-80% confluent HeLa cells for 24 h along with 8 µg/ml of Polybrene Transfection Reagent (Millipore Sigma).

GFP labeled HeLa cells were seeded in the 4 wells of 8-well chambers in the same manner as the HeLa cell described in step 2 of Supplementary Note S5. The wells with cell were transferred to the refrigerator (4°C) for 10 minutes the next morning.

(3) Incubation of cells with 1% BSA solution:

The solution in each well containing cells was replaced by 250 µL 1% BSA (DMEM) buffer and the cells were then incubated in the refrigerator (4°C) for 5 minutes.

(4) Staining of live cells with Integrin β1 primary antibody at 4°C:

10µL of Integrin β1 primary antibody stock at 200µg/mL was diluted to 4µg/mL by adding it to 490 µL cold 1% BSA (DMEM) buffer. After 5 minutes, the solutions in two of the wells containing cells were removed and 250 µL of diluted of Integrin β1 primary antibody solution was added to each well immediately. The solutions in the other two wells containing cells were each replaced by 250 µL 1% BSA (DMEM) buffer. The 4 wells of cells were then incubated in a refrigerator (4°C) for 60 minutes. After incubation, each well containing cells was washed by cold fresh DMEM on ice as described in Supplementary Note S26 step 5.

(5) Staining of live cells with biotinylated secondary antibody at 4°C:

The cells in the 4 wells were stained by biotinylated secondary antibody as described in Supplementary Note S26 step 6.

(6) Staining of live cells with neutravidin at 4°C:

1.5 μL of neutravidin stock (1 mg/mL in PBS buffer) was diluted to 3 μg/mL by adding it to 998.5 μL cold 1% BSA (DMEM) buffer. The solution in each well containing cells was removed and 250 μl of this diluted neutravidin solution was added to each well immediately. The cells were then incubated in a refrigerator (4°C) for 30 minutes. After incubation, the wells containing cells were washed by cold fresh DMEM 3 times in the manner as described in Supplementary Note S26 step 5 to remove the unattached neutravidin in solution.

(7) Coating of live HeLa cells with BDC tags at 4°C:

10 μL of the BDC tag stock (100 μM) was diluted to 1 μM by adding it to 990 μL cold 1% BSA (DMEM) buffer. The solution in each well containing cells was replaced by 250 μL of this diluted BDC tag solution. The cells were then incubated in a refrigerator (4°C) for 30 minutes. After incubation, the well containing cells were washed by cold fresh DMEM 3 times in the manner as described in Supplementary Note S26 step 5.

(8) Incubation of cells with seeds and seeded nanotubes:

The solution of PEG coated nanotube seeds was diluted to 40pM by cold 1% BSA (DMEM) -12mM $MgSO_4$ buffer. The solution in two of the wells with and without the addition of Integrin β1 primary antibody were replaced by 250 μL of this diluted seed solution. These cells with seeds were then incubated in a refrigerator (4°C) for 30 minutes.

The solution of PEG coated DNA seeded nanotubes was diluted 1-fold by a cold 1% BSA (DMEM)-12mM $MgSO_4$ buffer. The solutions in two of the other wells with and without addition of Integrin β1 primary antibody were replaced by 250 μL of this diluted seeded nanotube solution. These cells with nanotubes were then incubated in a refrigerator (4°C) for 2 hours.

After incubation, each well containing cells was washed by cold fresh DMEM-12mM $MgSO_4$ buffer 3 times in the manner as described in Supplementary Note S26 step 5 to remove the unattached seeded nanotubes in solution.

(9) Imaging of the cells under a spinning disk confocal microscope:

Each well containing cells was imaged as described in Supplementary Note S23 step 9.

The methods for quantifying the number of nanotube seeds and seeded nanotubes attached to the HeLa cells by integrin AMDA were the same as described in Supplementary Notes S22 and S27.

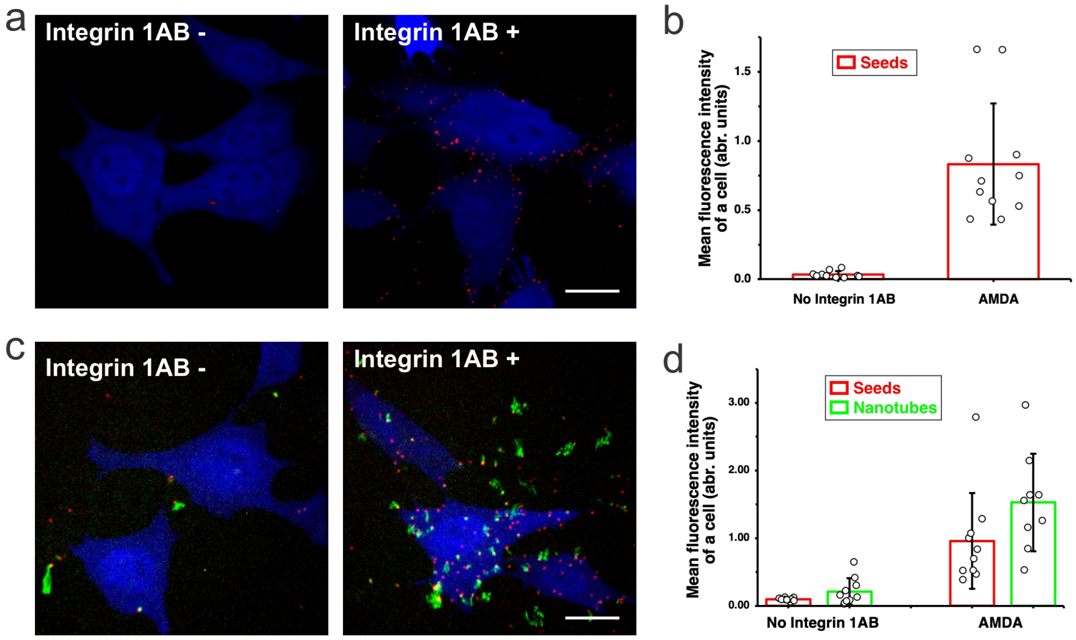

Supplementary Figure S15: Attachment of nanotube seeds and seeded nanotubes to integrin receptors on HeLa cells using integrin AMDA. Attachment and cell imaging were performed as described in Supplementary Note S30. Integrin primary antibodies were not added to the cells in the control groups. **(a)** 3D projection images of HeLa cells with PEG-coated nanotube seeds attached to integrin receptors on HeLa cells using integrin AMDA. HeLa cells were transfected with GFP (blue) and the nanotube seeds were labeled with atto647 (red). **(b)** The average fluorescence intensity in the seed channel per cell after integrin AMDA and after the control AMDA process where integrin antibodies were not added to cells. **(c)** 3D projection images of HeLA cells with PEG-coated seeded nanotubes attached to integrin receptors on HeLa cells. **(d)** The average fluorescence intensities in the nanotube seeds and seeded nanotube channels. Scale bars are 20µm. Error bars are standard deviations on intensity per field of view (N=10 fields of view).

Supplementary Note S31: Measurement of the rate of seed import into or detachment from HeLa cells

(1) Nanotube seed preparation:

Atto647 labeled PEG coated nanotube seeds with 6 BDC' strands on the ends of their barrels and 30 BDC' strands attached to their M13 scaffold loop region were prepared by combined the reagents in Supplementary Table S14 in Supplementary Note S26, then annealing as described in Supplementary Note S1 step 2, purifying them as in Supplementary Note S1 step 3 and coating them with PEG as described in Supplementary Note S3. The concentrations of the PEG-coated nanotube seeds were determined as described in Supplementary Note S1 step 3.

(2) GFP-labeled HeLa cell preparation:

The GFP labeled HeLa cell were seeded in a glass bottom 35mm μ-Dish as described below:
GFP labeled HeLa cell were cultured and passaged as HeLa cell described in the online methods. The cell concentration was determined by counted with a hemacytometer. The cells were then diluted to 2 x10^5 cell per mL by cell growth medium (DMEM medium containing 10% FBS and 1% penicillin-streptomycin). For each glass bottom 35mm μ-Dish (81148, Ibidi), 400 μL of this diluted cell solution was added to the inner well of the μ-Dish. The μ-dish was then carefully transferred to a cell culture incubator. Shaking the dish was avoided because this would result in an inhomogeneous distribution of the cells. After 1 hour of incubation, by which time the cells should have become attached to the glass surface, 1.6mL fresh cell growth medium was added to the dish to ensure optimal growth conditions. The μ-Dish was covered with the supplied lid and the cells were incubated in a cell culture incubator for overnight. The cells were used the next morning.

(3) Attachment of nanotube seeds to the cell membrane:

Nanotube seeds were attached to the GFP labeled HeLa cells through EGFR AMDA at 4°C as described in Supplementary Note S19 except that 3μg/mL neutravidin solution was added in step 7 and 32pM PEG coated nanotube seeds was added to the well with DBC tag added in step 9.

(4) Imaging of seed import or detachment from cells under a confocal microscope:

After attaching the nanotube seeds to the cells' membranes at 4°C, the wash medium (DMEM-12.5mM Mg^{2+}) was changed to normal growth medium with 12mM $MgSO_4$, and the cells were put in an incubator set to 37°C, 5% CO_2 humidity on a Nikon confocal microscope. Z stack images from cell bottom to cell top with a stack height of 1 μm were taken every 10 minutes for 70 minutes by NIS-Elements AR 5.02.01 (Nikon).

Supplementary Note S32: Measurement of the rate of seeded nanotube import into or detachment from HeLa cells

(1) Seeded nanotube preparation:

PEG-coated seeded nanotubes were prepared as described in Supplementary Note S26 step 1.

(2) GFP labeled HeLa cell preparation:

The GFP labeled HeLa cells were seeded in a glass bottom 35mm μ-Dish as described in Supplementary Note S31 step 2.

(3) Attachment of seeded nanotubes to the cells' membranes:

The seeded nanotubes were attached to GFP labeled HeLa cell through EGFR AMDA at 4°C as described in Supplementary Note S26, except that instead of using Alexa 488 labeled streptavidin in step 6, the cells were coated with neutravidin as described in Supplementary Note S30 step 6.

(4) Imaging of seeded nanotube import into or detachment from cells under a confocal microscope:

After attaching the seeded nanotubes to the cells' membranes at 4°C, the wash medium (DMEM-12.5mM Mg^{2+}) was changed to normal growth medium (the DMEM medium containing 10% FBS and 1% penicillin-streptomycin) with 12mM $MgSO_4$. The cells were put in an incubator set to 37°C, 5% CO_2 humidity on a Nikon confocal microscope. Z stack images from cell bottom to cell top with a stack height of 1 μm were taken every 30 minutes for 12 hours by NIS-Elements AR 5.02.01 (Nikon). We found that almost all of the seeded nanotubes anchored on the HeLa cells were no longer attached after to the cells after 6 hours. Previous studies of PEG coated seeded nanoubes showed that the length of the PEG coated seeded nanotube did not changed too much after incubating in the serum-supplemented medium (10% FBS and 12.5mM $MgCl_2$ in DMEM) at 37°C for 6 hours,[2] suggesting that the effects of nuclease mediated degradation were not driving this disappearance. Tracking nanotubes over shorter time frames (as in Figure 3g-h in the main text) rather suggested that nanotubes disappeared suddently from the cell surface, perhaps because they were endocytosed.

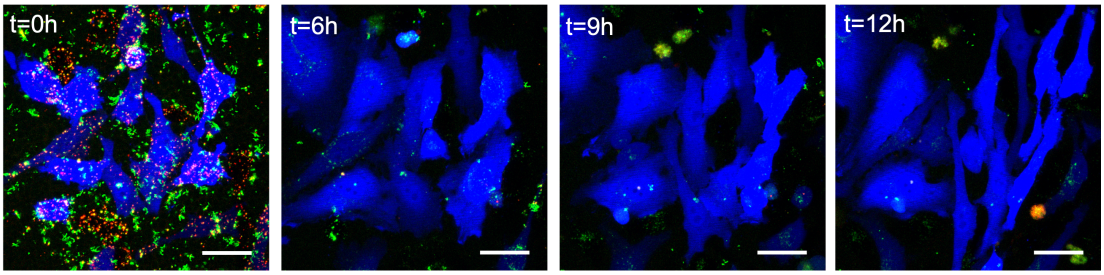

Supplementary Figure S16: Maximum projection images of HeLa cells at different times after seeded nanotube attachment using EGFR AMDA. HeLa cells were transfected with GFP (blue), the nanotube seeds were labeled with atto647 (red) and the nanotubes with Cy3. There was no obvious decrease in cell viability 12 hours after seeded nanotube attachment. Scale bars are 40μm. This experiment was repeated more

than three times independently with similar results.

Supplementary Note S33: Measuring the persistence time of seeds and seeded nanotubes on the HeLa cell membrane after EGFR AMDA

To quantitively measure the times over which nanotube seeds and seeded nanotubes persisted on the cell membrane after being attached, we tracked individual cells over time and measured the total number of nanotube seeds or seeded nanotubes on the membrane of each tracked cell at a set of time points (Supplementary Notes S31-32). At each time point, the total number of seeds on the cell membrane was calculated by summing the number of seeds in each of a stack of confocal micrographs. When counting nanotube seeds, the bottom and top stacks were excluded from this sum: it was hard to distinguish in the bottom stack image whether the seeds that were visible were attached to the cell membrane or to the slide, it was hard to determine in the top image whether the seeds were inside the cell or on the cell membrane. When counting seeded nanotubes, the bottom and top images were included in analysis: it was possible to determine whether the nanotubes were on the cell membrane (external to the cell) or inside the cell since nanotubes inside the membrane did not remain intact and nanotubes attached to the top of the cell could be distinguished because the nanotubes (but not their seed anchors) moved from frame to frame. Further, including these images was necessary to have sufficient data for analysis: the DNA nanotubes had an average length of 3±0.2μm (Supplementary Figure S5) and the average height of an adherent HeLa cell is not much larger than this. It would reduce the cell surface area that could be analyzed by at least 50% if we excluded all the bottom stacks which might contain nanotubes that conceivably might be attached to a slide rather than the cell.

Section 4: Nanotubes as flow meters on the live cell membrane

Supplementary Note S34: Simulation of nanotube position in a flow field

34.1 The flow in a fluid cell chamber

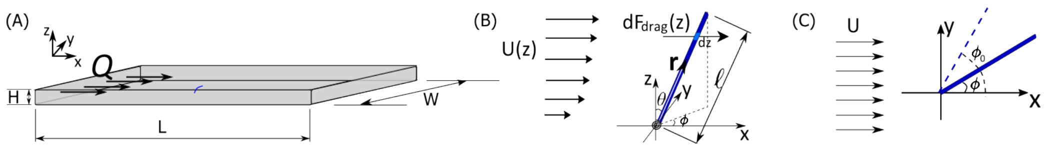

Supplementary Figure S17: (a) Diagram of a DNA nanotube in a rectangular chamber. **(b)** Diagram of DNA nanotube deflection under flow *Q*. The polar angle θ is the angle between the DNA nanotube and the *z*-axis, and ϕ is the azimuth angle between the plane of the DNA nanotube and the x-axis. **(c)** The rotation of a DNA nanotube about its anchor from the top view. ϕ_0 is the initial azimuth angle of a DNA nanotube without external flow; this is presumably different for different DNA nanotubes. ϕ is the azimuth angle of a DNA nanotube under a given flow. In the model the problem can be simplified such that $\theta = \pi/2$ and the fluid profile around the DNA nanotube is uniform.

To model the fluid cell chamber, we denote the height, width, and length of the chamber as *H*, *W*, and *L*, respectively (Supplementary Figure S17a). Given volumetric flow rate *Q*, the average cross-sectional velocity is

$$\bar{U} = \frac{Q}{A_0} \tag{4.1}$$

Where $A_0 = H \times W$ is the cross-sectional area of the chamber. Since $H << W, L$, we can assume that the flow is uniform in width and length and only varies along the height direction (*z*) ($0 < z < H$). For a Stokes flow, the velocity profile can be solved as (Supplementary Figure S17b)

$$U(z) = \frac{1}{2\mu}\frac{\partial p}{\partial x}(z^2 - Hz), \tag{4.2}$$

where μ is the dynamic viscosity of the fluid. Integrating Eq. 4.2 over the entire cross section gives the volumetric flow rate *Q*, so the pressure gradient can be expressed in *Q*, *i.e.,*

$$\frac{\partial p}{\partial x} = -\frac{12\mu}{H^3 W}Q. \tag{4.3}$$

Substituting Eq. 4.3 into Eq. 4.2 gives the velocity in terms of the volumetric flow rate,

$$U(z) = \frac{6Q}{H^3 W}(Hz - z^2) \tag{4.4}$$

This flow profile was used to calculate the fluid drag on a DNA nanotube.

The shear stress can be obtained from the flow profile:

$$\tau = \mu \frac{\partial U(z)}{\partial z} = \mu \frac{6Q}{H^3 W}(H - 2z) \quad (4.5)$$

From this equation, we can see that when z << H, the shear stress is approximated by the wall shear stress at the lower surface of the chamber:

$$\tau(z = 0) = \frac{6Q\mu}{H^2 W} \quad (4.6)$$

The heights of adherent HeLa cell are on average 7-10µm, very small compared with the flow chamber height (H=400 µm). We assumed that the shear stress on the HeLa cell surface was equal to the wall shear stress.

Supplementary Figure S18 shows the shear stress at the lower surface of the chamber as a function of *Q*, given the dimensions of the chamber used in experiments (H= 0.54mm, W= 3.8mm and L=17mm).

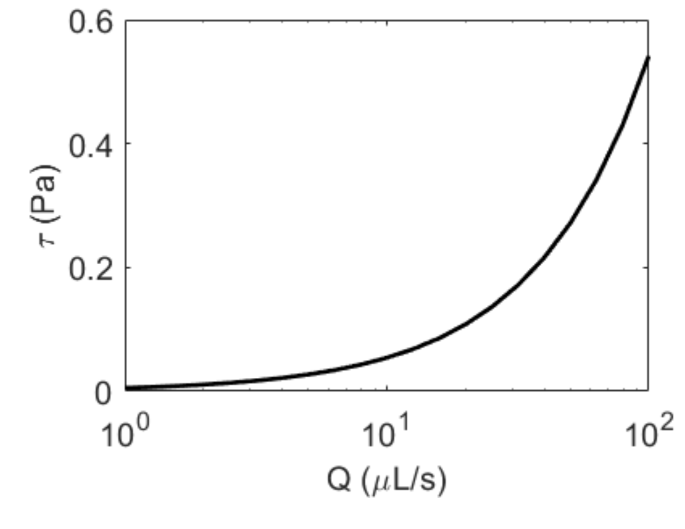

Supplementary Figure S18: Shear stress on the lower surface of the chamber as a function of Q.

34.2 A full model of the bending of a DNA nanotube in a flow field.

We developed a full model of the bending of a DNA nanotube in a flow field that predicts both the polar and azimuth angles of the nanotube and allows the flow profile to vary in the vertical direction.

To estimate the drag on a DNA nanotube, the following approximations are made. (1) Each DNA nanotube is a rigid rod that rotates at the pivot attached to the substrate. (2) Under a low Reynolds approximation, a DNA nanotube does not perturb the flow field. (3) When each DNA nanotube is tilted at angle θ with respect to the z-axis, the cross section of the DNA nanotube perpendicular to the flow is still approximated as circular so that the rod can be treated as a cylinder for each vertical segment dz.

The Stokes flow around a cylinder can be approximated by a stream function expanded in the Reynolds number, Re. The pressure and shear stress on the DNA nanotube are then calculated from the approximated stream function. An estimation of the fluid drag on each dz segment of the DNA nanotube is[18,19]

$$dF_{\text{drag}} = \frac{4\pi\mu U(z)}{1/2 - \gamma - \ln\left[\rho U(z) R / 4\mu\right]} dz \tag{4.7}$$

where R is the radius of the DNA nanotube, ρ is the fluid density, and $\gamma = 0.5772$ is the Euler constant. The force is in the x-direction, *i.e.,* $d\boldsymbol{F}_{\text{drag}} = (dF_{\text{drag}}, 0, 0)$.

There are two angles associated with DNA nanotube deflection. One is the angle between a DNA nanotube and the z-axis, θ, and the other is the angle between the plane of a DNA nanotube and the x-axis, ϕ. For any point $\boldsymbol{r}$ on a DNA nanotube, $|\boldsymbol{r}| = r \in [0, \ell]$, where ℓ is the length of a DNA nanotube, the direction of the point in terms of its Cartesian coordinates is given by

$$\boldsymbol{r} = (x, y, z) = (r \sin\theta \cos\phi, r \sin\theta \sin\phi, r \cos\theta) \tag{4.8}$$

The infinitesimal moment on a DNA nanotube for each dz segment is

$$d\mathbf{M} = \mathbf{r} \times d\mathbf{F}_{\text{drag}} = r\cos\theta dF_{\text{drag}}\mathbf{e}_{\text{y}} - r\sin\theta\sin\phi dF_{\text{drag}}\mathbf{e}_{\text{z}} = dM_y\mathbf{e}_{\text{y}} + dM_z\mathbf{e}_{\text{z}} \tag{4.9}$$

The force and momentum can be decomposed into spherical coordinates. In the expression of dF_{drag}, the infinitesimal element becomes $dz = \cos\theta\, dr - r\sin\theta\, d\theta$. When θ is fixed, the $d\theta$ component can then be dropped. In this case, the magnitude of the infinitesimal drag is

$$dF_{\text{drag}} = A_1 \frac{\left(Hr\cos\theta - r^2\cos^2\theta\right)\cos\theta dr}{A_2 - \ln\left(Hr\cos\theta - r^2\cos^2\theta\right)} \tag{4.10}$$

where

$$A_1 = \frac{24\pi\mu Q}{H^3 W}, \quad A_2 = \frac{1}{2} - \gamma - \ln\frac{3\rho R Q}{2H^3 W\mu}. \tag{4.11}$$

The drag is only in the x-direction, so it can be decomposed as

$$\hat{\mathbf{x}} = \sin\theta\cos\phi\hat{\mathbf{r}} + \cos\theta\cos\phi\hat{\boldsymbol{\theta}} - \sin\phi\hat{\boldsymbol{\phi}}. \tag{4.12}$$

The vector form of the infinitesimal drag in spherical coordinates is therefore

$$d\mathbf{F}_{\text{drag}} = dF_{\text{drag}}\left(\sin\theta\cos\phi\hat{\mathbf{r}} + \cos\theta\cos\phi\hat{\boldsymbol{\theta}} - \sin\phi\hat{\boldsymbol{\phi}}\right) = dF_{\text{drag},r}\hat{\mathbf{r}} + dF_{\text{drag},\theta}\hat{\boldsymbol{\theta}} + dF_{\text{drag},\phi}\hat{\boldsymbol{\phi}}. \tag{4.13}$$

The infinitesimal moment in spherical coordinates is

$$d\mathbf{M} = \mathbf{r}\times d\mathbf{F}_{\text{drag}} = rdF_{\text{drag},\theta}\hat{\boldsymbol{\phi}} - rdF_{\text{drag},\phi}\hat{\boldsymbol{\theta}}, \tag{4.14}$$

so we have $dM_\theta = -rdF_{\text{drag},\phi}$ and $dM_\phi = rdF_{\text{drag},\theta}$. The total moment is

$$\mathbf{M} = \int_0^\ell d\mathbf{M} \tag{4.15}$$

The component associated with the angle θ is

$$M_\theta = \int_0^\ell dM_\theta = -\int_0^\ell rdF_{\text{drag},\phi} = \int_0^\ell r\sin\phi dF_{\text{drag}} = A_1\int_0^\ell \frac{\left(Hr\cos\theta - r^2\cos^2\theta\right)r\cos\theta\sin\phi}{A_2 - \ln\left(Hr\cos\theta - r^2\cos^2\theta\right)}dr \tag{4.16}$$

and the component associated with the angle ϕ is

$$M_\phi = \int_0^\ell dM_\phi = \int_0^\ell rdF_{\text{drag},\theta} = \int_0^\ell r\cos\theta\cos\phi dF_{\text{drag}} = A_1\int_0^\ell \frac{\left(Hr\cos\theta - r^2\cos^2\theta\right)r\cos^2\theta\cos\phi}{A_2 - \ln\left(Hr\cos\theta - r^2\cos^2\theta\right)}dr. \tag{4.17}$$

Solving for θ and ϕ for a given Q and ℓ: A Static Approach

The two angles are coupled to the flow Q and should be solved with two sets of equations. The pivot may be modeled as a rotational spring that rotates away from the z-axis with rotational rigidity G_θ, i.e.,

$$M_\phi = \theta G_\theta. \tag{4.18}$$

Without flow, it is assumed that the DNA nanotube has an azimuth angle ϕ_0

(Supplementary Figure S17c) and the azimuth rotational rigidity is G_ϕ. Under flow, the angle becomes ϕ; the difference, $\phi_0 - \phi$, is contributed by the moment from the flow, i.e.,

$$M_\theta = (\phi_0 - \phi)G_\phi. \tag{4.19}$$

Equations 4.18 and 4.19 are nonlinear in θ and ϕ and there is no explicit expression of either equation. These two equations were solved using Newton iteration by finding the gradient on a numeric scheme. Let

$$\mathbf{X} = \begin{bmatrix} \theta \\ \phi \end{bmatrix}, \quad \mathbf{F} = \begin{bmatrix} f_1 \\ f_2 \end{bmatrix} \tag{4.20}$$

where

$$f_1 = M_\phi - \theta G_\theta, \quad f_2 = M_\theta - (\phi_0 - \phi)G_\phi. \tag{4.21}$$

The gradient is calculated from

$$\mathbf{\Omega} = \begin{bmatrix} \dfrac{\partial f_1}{\partial \theta} & \dfrac{\partial f_1}{\partial \phi} \\ \dfrac{\partial f_2}{\partial \theta} & \dfrac{\partial f_2}{\partial \phi} \end{bmatrix} = \begin{bmatrix} \dfrac{f_1(\theta+\Delta\theta,\phi) - f_1(\theta,\phi)}{\Delta\theta} & \dfrac{f_1(\theta,\phi+\Delta\phi) - f_1(\theta,\phi)}{\Delta\phi} \\ \dfrac{f_2(\theta+\Delta\theta,\phi) - f_2(\theta,\phi)}{\Delta\theta} & \dfrac{f_2(\theta,\phi+\Delta\phi) - f_2(\theta,\phi)}{\Delta\phi} \end{bmatrix} \tag{4.22}$$

Then

$$\mathbf{X}_{n+1} = \mathbf{X}_n - \mathbf{\Omega}^{-1}\mathbf{F} \tag{4.23}$$

from which the two angles can be solved.

Supplementary Figure S19 shows the rotational angles θ and ϕ as functions of flow rate, Q, for different G_θ and G_ϕ. Supplementary Figure S20 shows the rotational angles θ and ϕ as functions of DNA nanotube length, ℓ, for different G_θ and G_ϕ.

This solution provides deterministic solutions for the two angles for a given set of parameters. In reality, a distribution of such angles is seen. This distribution could arise because the initial value of the azimuth angle matters in determining the final angle. If we assume that ϕ_0 is randomly distributed for different DNA nanotubes, we can consider this distribution by producing a histogram of ϕ values under different flow rates for a random distribution of ϕ_0. Supplementary Figure S21 shows histogram of ϕ for 4 example Q values produced using this approach. Note that the exact distribution of ϕ depends on the other parameters as well.

Supplementary Figure S21 shows that even considering this distribution of angles,

DNA nanotubes are on average more aligned with the direction of the flow ($\phi = 0$) when the flow rate is larger.

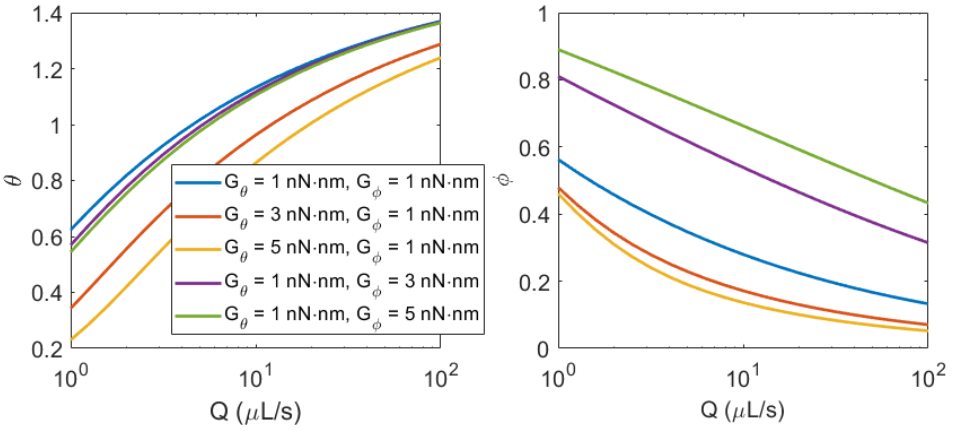

Supplementary Figure S19: Rotational angles θ and ϕ (in radians) as functions of Q for different G_θ and G_ϕ.

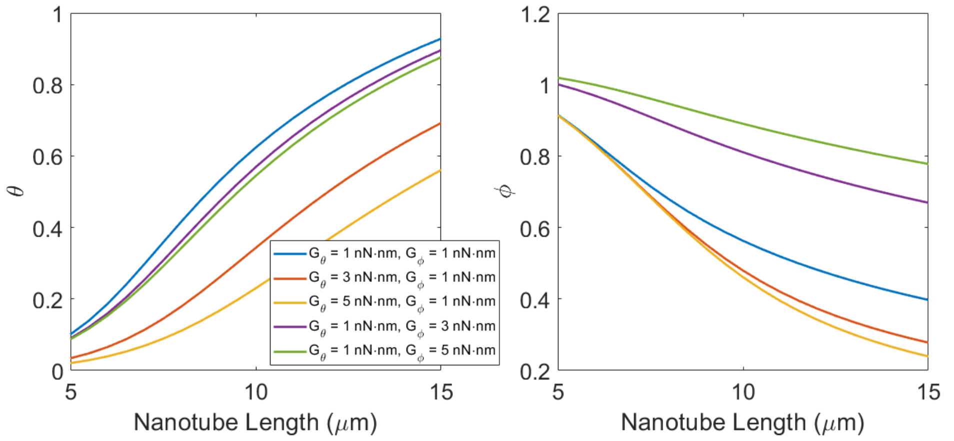

Supplementary Figure S20: Rotational angles θ and ϕ (in radians) as functions of ℓ for different G_θ and G_ϕ.

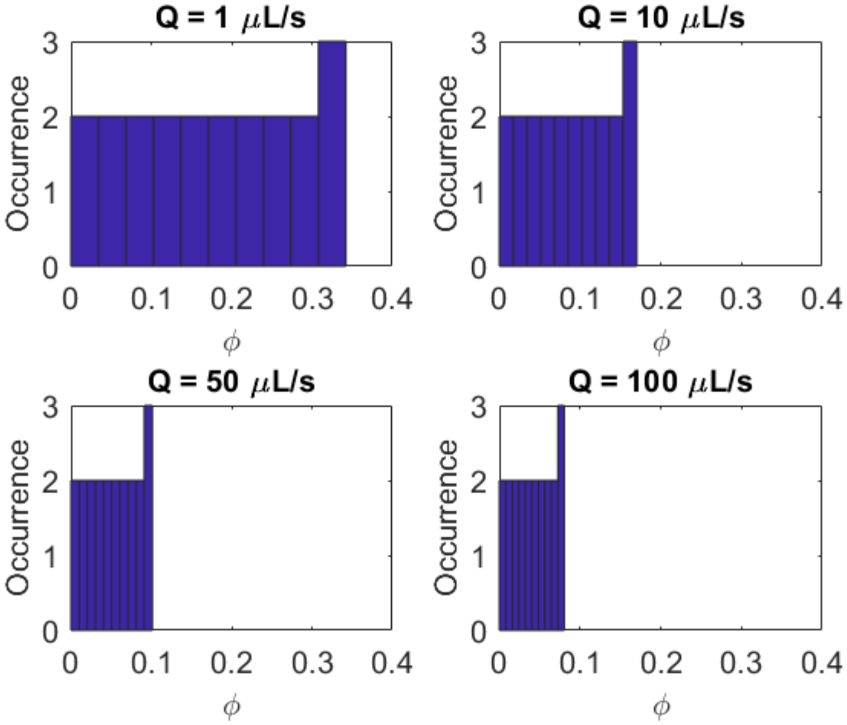

Supplementary Figure S21: Histograms of ϕ for different Q. Here $G_\theta = G_\phi = 1$ nN·nm.

Solving for θ and ϕ for given Q and ℓ: Dynamic approach

The dynamics of the two angles can be solved without assuming the system is under force balance. The spring contribution associated with the two angles can also be removed. The equations for motion are:

$$\gamma\dot{\theta} = M_\phi + R_\theta, \quad -\gamma\dot{\phi} = M_\theta + R_\phi \tag{4.24}$$

where γ is the damping coefficient of the DNA nanotube and R_θ and R_ϕ are random forces from thermal fluctuation. The distribution of R is given by

$$P(R_\theta) \propto e^{-\frac{R_\theta^2 \Delta t}{2k_B T\gamma}}, \quad P(R_\phi) \propto e^{-\frac{R_\phi^2 \Delta t}{2k_B T\gamma}} \tag{4.25}$$

where Δt is the time step used to numerically evolve Eq. 4.24. For each time step, t, random R_θ and R_ϕ are drawn from the distribution in Eq. 4.25. The initial value of the azimuth angle, ϕ_0, of each DNA nanotube is randomly drawn from a uniform distribution between $-\pi$ and π. The probability distribution of θ and ϕ is solved from a large number of DNA nanotubes for each volume flow rate Q. Supplementary Figure S22 shows the model prediction of the distribution of θ and ϕ and the MSD of θ and ϕ for different volume flow rates.

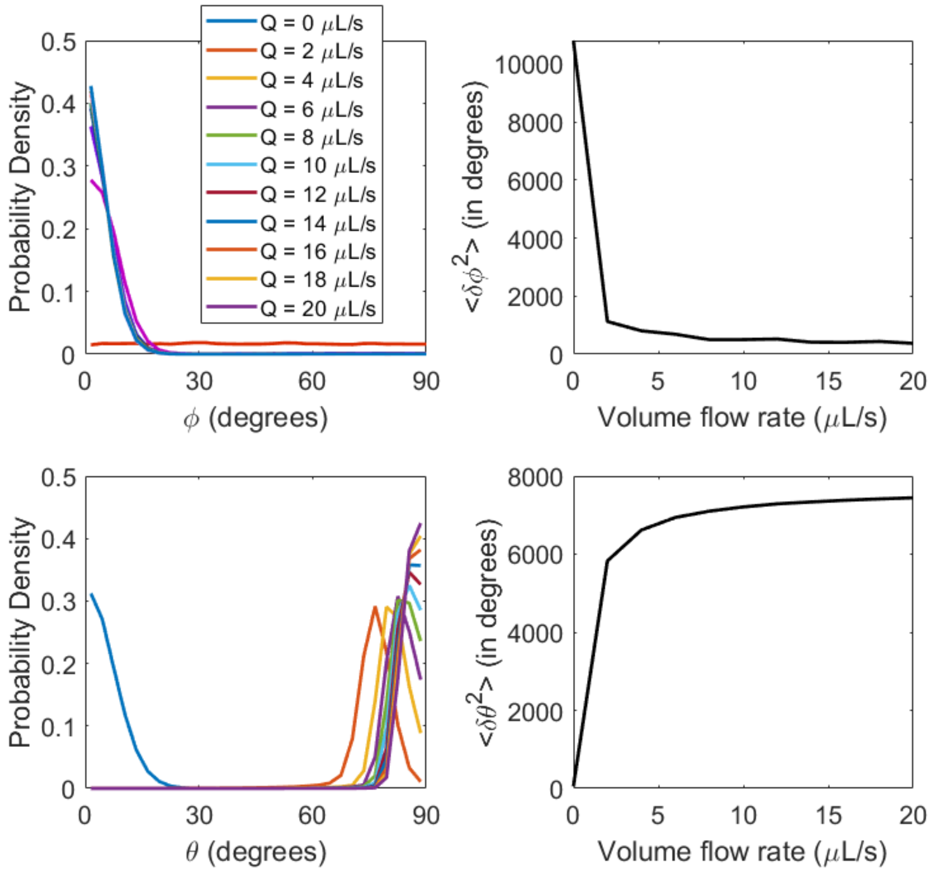

Supplementary Figure S22: Model of the distribution of θ and ϕ and the MSD of θ and ϕ for different volume flow rates**.**

Total force on a DNA nanotube

Here we would like to compute the total force from fluid viscous drag on a DNA nanotube with given length, ℓ. The magnitude of the total force on a DNA nanotube is independent of the direction of the force. Therefore, Eq. 4. 10 (force along the *r*-direction) can be used to obtain the force,

$$F_{\text{drag}} = \int_0^{\ell} dF_{\text{drag}} = A_1 \int_0^{\ell} \frac{\left(Hr\cos\theta - r^2\cos^2\theta\right)\cos\theta}{A_2 - \ln\left(Hr\cos\theta - r^2\cos^2\theta\right)} dr \qquad (4.26)$$

Supplementary Figure S23 shows the forces on a DNA nanotube as functions of DNA nanotube length for different polar angles and flow rates.

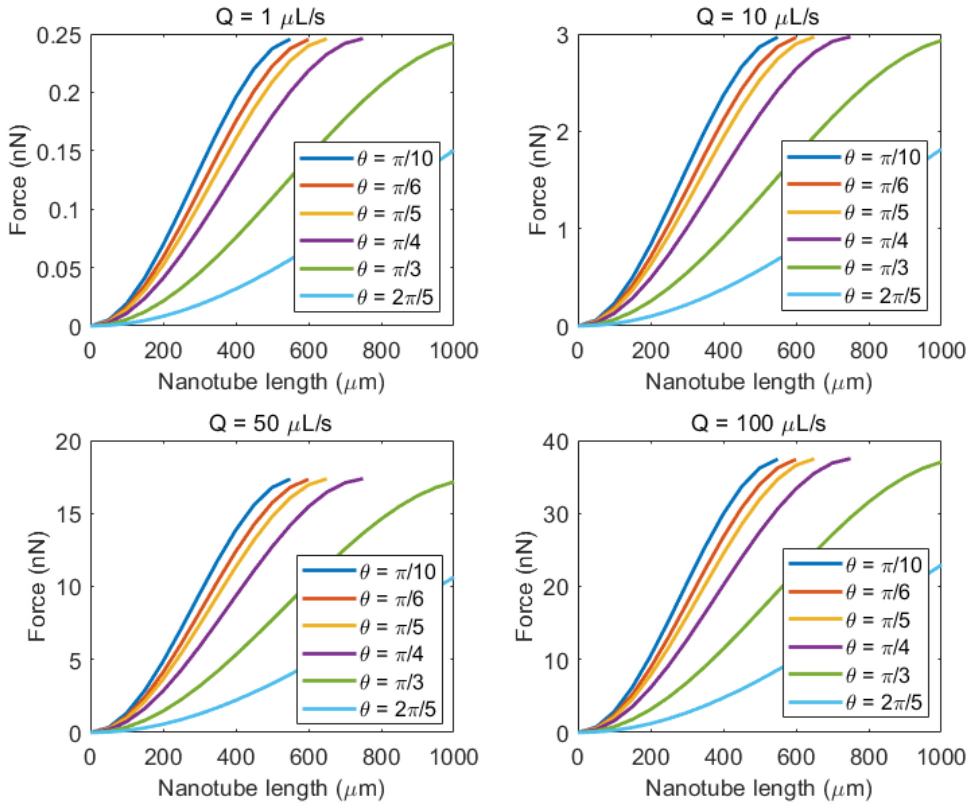

Supplementary Figure S23: Total force on a DNA nanotube as a function of DNA nanotube length for different polar angles and flow rates.

Supplementary Table S15: Default parameters in the full model

Parameters	H (mm)	W (mm)	L (mm)	Q (μL/s)	ℓ (μm)	R (nm)	μ (Pa s)	ρ (kg/m^3)	ϕ_0
Values	0.54	3.8	17	10	10	10	0.001	1000	π/6

34.3 A simplified model

Given that the length of the DNA nanotube is much smaller than the height of the chamber, it can be assumed that the flow around the DNA nanotube is uniform (Supplementary Figure S17c) and the flow velocity, U, is just the average velocity given in Eq. 4.**Error! Reference source not found.**. The over-bar on U will be thus be d ropped in what follows. Based on the predictions of the model shown in Supplementary Figure S22, it can be seen that the polar angle is close to $\pi/2$, except at very small volumetric flow rates. The problem can be simplified further given that the polar angle is always $\pi/2$. These simplifications make it possible to solve for the orientation of a DNA nanotube in a 2D plane.

Drag and torque on a DNA nanotube for low Re

Since we have assumed uniform flow, the viscous drag from the flow on the DNA nanotube is

$$\mathbf{F} = (\alpha\mu U\ell, 0) \tag{4.27}$$

where α is the coefficient of viscous drag on the DNA nanotube. The direction vector of the center of mass of the DNA nanotube is

$$\mathbf{r} = \left(\frac{1}{2}\ell\cos\phi, \frac{1}{2}\ell\sin\phi\right) \tag{4.28}$$

The torque on the DNA nanotube is

$$\mathbf{M} = \mathbf{r} \times \mathbf{F} = -\frac{1}{2}\alpha\mu U\ell^2\sin\phi \tag{4.29}$$

We will use this torque to calculate the motion of the DNA nanotube.

Solving for ϕ for a given Q and ℓ

The motion of the DNA nanotube is given by

$$\gamma\dot{\phi} = M + R \tag{4.30}$$

where γ is the damping coefficient of the DNA nanotube and R is a random force from thermal fluctuation. The distribution of R is given by

$$P(R) \propto e^{-\frac{R^2\Delta t}{2k_BT\gamma}} \tag{4.31}$$

where Δt is the time step used to numerically evolve Eq. 4.30. For each time step, t, a random R is drawn from the distribution in Eq. 4.31. The initial value of the azimuth angle, ϕ_0, of each DNA nanotube is randomly drawn from a uniform distribution between $-\pi$ and π. The probability distribution of ϕ is then sampled by solving for a

large number of DNA nanotubes with different ϕ_0 samples for each volume flow rate Q. Supplementary Figure S24 shows the model's predicted distribution of θ and ϕ and the MSD of θ and ϕ for different volume flow rates. For comparison, experimental data is attached in Figure 4h. A difference can be seen between this simplified model's prediction and experimental data.

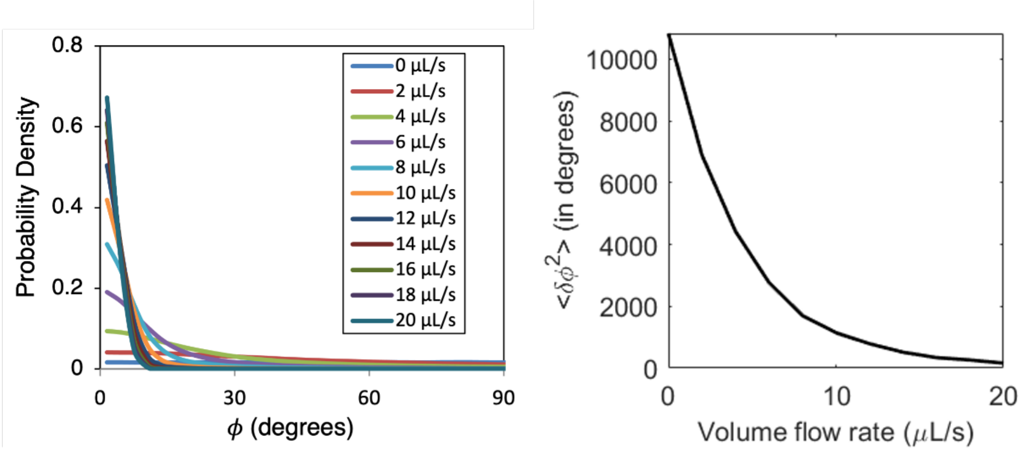

Supplementary Figure S24: Model prediction (a simplified model) of the distribution of ϕ and the MSD of ϕ for different volume flow rates.

Supplementary Table S16: Default parameters in the simplified model

Parameters	H (mm)	W (mm)	L (mm)	ℓ (µm)	R (nm)	μ (Pa s)	γ (kg m^2/s)	α
Values	0.54	3.8	17	10	10	0.001	3.5×10^{-18}	0.001

Supplementary Note S35: Determination of the shear stresses induced by different laminar flow rates in different flow cells

Shear stress was applied to nanotubes using laminar flows at controlled rates. The amount of shear stress applied by a given flow rate in a given channel calculated according to the formula provided by the channel manufacturer (Ibidi).

For the glass-bottom μ-slide channels (height 0.54 mm, length 17 mm and width 3.8 mm) that were used to study the effects of shear stress on nanotubes anchored to glass, the magnitude of shear stress was determined using the formula:

$$\tau = \eta \times 104.7 \times Q. \tag{4.32}$$

For the plastic-bottom μ-slide channels (height 0.4 mm, length 17 mm and width 3.8 mm) that were used to study the effects of shear stress on nanotubes anchored to cells, the magnitude of shear stress was determined using the formula:

$$\tau = \eta \times 176.1 \times Q, \tag{4.33}$$

where τ = shear stress (dyn/cm^2), η = dynamical viscosity (dyn s/cm^2), and Q = flow rate (mL/min) in each of the formulas. The difference between the two formulae is due to the slight difference in heights of the two channels.[20]

1xTAE-Mg^{2+} buffer and DMEM-12.5mM $MgSO_4$ buffer were used as flow perfusates for applying shear stress to nanotubes anchored to glass and to cell membranes, respectively. Both types of experiments were conducted at room temperature (19~21°C). We used the viscosity of water at 20 ºC for the viscosities of both TAE-Mg^{2+} buffer and the DMEM-12.5mM $MgSO_4$ buffer, *i.e.*

$$\eta = 0.01 \text{ dyn s/cm}^2. \tag{4.34}$$

The flow rates applied to the seeded nanotubes anchored to glass surfaces and to cells and their respective expected shear stresses are shown in Supplementary Table S17.

Supplementary Table S17: Fluid flow shear stresses and flow rates for nanotubes on cell membranes and glass surfaces

τ (dyn/cm^2)	Q in μ-slide VI 0.4 (nanotubes on cells) (mL/min)	Q in μ-slide VI 0.5 (nanotubes on glass) (mL/min)
0.05	0.03	0.05
0.11	0.06	0.10
0.21	0.12	0.20
0.42	0.24	0.40
0.63	0.36	0.61
0.85	0.48	0.81
1.1	0.6	1.0
1.3	0.72	1.2

1.7	0.96	1.6
2.1	1.2	2.0

Supplementary Note S36: Protocols for treating glass bottom channels to anchor seeded nanotube on their surfaces

(1) The glass bottom 6 channel μ slide (Ibidi, μ-slide VI 0.5) was cleaned by immersing in 10% NaOH and sonicating for 60min.

(2) The glass bottom 6 channel μ slide was then washed with water 3 times. For each wash cycle, the slide was totally immersed in fresh water. This step removed the 10% NaOH residue on the slide but not in the channels.

(3) To further remove the NaOH residue inside the channels, the water in the reservoirs at both ends of each channel was first removed while keeping the channel filled with water. The channel was washed with water 3 times by pipetting 160 μL water into one reservoir of the channel and aspirating from the other reservoir at the same time. It was critical that the channel not dry, so care was taken to not aspirate the water in the channel with the aspirator. Then each channel was washed with methanol 3 times in the same manner, thus keeping the channel filled with methanol at all times.

(4) For each channel, 280μL of a solution of 1mg/mL biotin PEG silane (MW 3400) solution in buffer containing 95% methanol, 4% acetic acid, and 1% water was prepared. 160 μL of this solution was added to one reservoir while simultaneously aspirating from the other reservoir and being careful to not aspirate the solution in the channel. The reservoirs were then refilled with 60μL biotin PEG silane solution. The channel was sealed with Parafilm to prevent evaporation and left overnight.

(5) The next day, the Parafilm on the channel was removed. The channel was washed with methanol 3 times and followed with water 3 times, both in the same manner as described in step 3. The water in each channel and reservoir was then blown off by nitrogen gas.

(6) The 6 channel μ slide in was then incubated in an oven at 70°C for 1 hour.

(8) For each channel, 280μL of 1% BSA solution in TNT buffer (10 mM Tris-HCl, 0.05% Tween-20, 0.1 M NaCl, pH 7.5) was prepared. 160 μL of this solution was added to one reservoir and aspirated from the other reservoir at the same time without aspirating the solution in the channel. Then both of the reservoirs were refilled with 60μL 1% BSA solution and incubated for 1.5 hours. After incubation, each channel was washed with TNT buffer 3 times in the same manner as described in step 3 to remove excess 1% BSA solution.

(9) For each channel, 280μL 0.2mg/mL neutravidin (31000, Thermo Fisher Scientific) in TNT buffer was prepared. 160 μL of this solution was added to one reservoir and aspirated from the other reservoir at the same time without aspirating the solution in the channel. Then both of the reservoirs were refilled with 60μL neutravidin solution and incubated for 2 hours. After incubation, each channel was washed with TNT

buffer 3 times in the same manner as described in step 3 to remove excess neutravidin solution. Then each channel was washed with 1 × TAE-Mg^{2+} buffer 3 times as the same manner described in step 3. Both of the reservoirs of each channel were refilled with 60 µL 1 × TAE Mg^{2+} buffer. The channel was then sealed with Parafilm until use.

Supplementary Note S37: Protocol for attaching seeded nanotubes to the glass bottom of a µ-slide channel.

(1) Seeded nanotube preparation:
PEG coated 6nt seeded nanotubes were prepared as described in Supplementary Note S26 step 1.

(2) Modification of the glass surface of the channel with PEG silane and attachment of neutravidin:

The glass bottom of the channel of the µ-slide was modified with neutravidin by creating a biotin-labeled PEG silane monolayer and attaching neutravidin using the protocol described in Supplementary Note S36.

(3) Attachment of BDC tag to the glass surface of the channel:

The TAE-Mg^{2+} buffer in both reservoirs of the channel were carefully removed. 100 µL 1µM BDC tag (in 1xTAE-Mg^{2+} buffer) solution was added to one reservoir of the channel and aspirated from the other reservoir at the same time without aspirating the solution in the channel. Then both of the reservoirs were filled with 25µL BDC tag solution and incubated for 10 minutes.

(4) Washing the channel with 1x TAE-Mg^{2+} buffer:

The channel was then washed with 1x TAE-Mg^{2+} buffer three times to remove the excess BDC tag in the channel. For each wash cycle, 160 µL 1xTAE-Mg^{2+} buffer was pipetted into one reservoir of the channel and aspirated from the other reservoir at the same time without aspirating the solution in the channel. Then both of the reservoirs were refilled with 25µL 1xTAE-Mg^{2+} buffer.

(6) Attachment of the seeded nanotubes to the glass surface of the channel:

100 µL of the seeded nanotubes prepared in step 1 was added to one reservoir of the channel and aspirated from the other reservoir at the same time without aspirating the solution in the channel. Then both of the reservoirs were filled with 25µL seeded nanotube solution and incubated for 5 minutes. The incubation time was kept short to prevent too many seeded nanotubes from attaching to the surface, as a high density of nanotubes would make it difficult to characterize individual nanotubes in micrographs. After incubation, unattached seeded nanotubes were washed away with 1xTAE-Mg^{2+} buffer in the manner described in step 3.

Supplementary Note S38: Characterizing the responses of seeded nanotubes anchored on a glass surface to differing amounts of shear stress induced by laminar fluid flow

PEG-coated seeded nanotubes were anchored to the glass surface of a μ slide channel as described in Supplementary Note S 37. A syringe pump (New Era, NE-1000) was used to provide uni-directional laminar flow through the channel when a syringe (DB 20ml syringe luer-lok tip, REF 305617) and tube adapter (Ibidi 10831) were connected to the channel on the μ-slide. 1xTAE-Mg^{2+} buffer was used as a flow perfusate. A series of flow rates in order from lowest to highest were applied to the nanotubes attached to the glass as follows: 0, 0.05, 0.10, 0.20, 0.40, 0.61, 0.81, 1.01, 1.21, 1.62 and 2.02 mL per minute. A spinning disk confocal microscope was used to capture images of the seeded nanotubes while flow was applied. For each flow rate, images were taken every 5 seconds for 145 seconds, so that 30 images for each flow rate were captured.

Supplementary Note S39: Protocol for attaching seeded nanotubes to EGFR receptors on HeLa cells using the EGFR AMDA in μ-slide channels

(1) Seeded nanotube preparation:

PEG coated 6nt seeded nanotubes were prepared as described in Supplementary Note S26, Step 1.

(2) HeLa cell seeding in channels:

HeLa cell were seeded in the channels of the μ-slide as described below:

HeLa cells were released from a flask surface using 0.05% Trypsin-EDTA, and the concentration of cells was counted by hemacytometer. The HeLa cells were then diluted to 9×10^5 cells per mL using cell growth medium (DMEM medium containing 10% FBS and 1% penicillin-streptomycin). 30 μL diluted cell suspension was added to the channel of a μ-slide (Ibidi, μ-slide VI 0.4) with a polymer coverslip bottom. Quickly dispensing the cell solution helped to avoid trapping air bubbles in the channel. The reservoirs of the channel were covered with the supplied lid. The μ-slide was transferred to a cell culture incubator set to 37 °C and 5 % CO2 and incubated for 30 minutes to let the cell attach to the surface. After 30 minutes, each luer reservoir was filled with 60 μL cell growth medium. The μ-slide with cells was transferred to the cell culture incubator and incubated overnight. The cell was ready be used in the next morning.

The next morning, the μ-slide was moved into a refrigerator (4°C) and incubated for 10 minutes. As in the protocol in Supplementary Note S15, all the buffers and AMDA reagent (antibodies, neutravidin and BDC tag) stocks used in the following steps were kept on ice and the cell were also kept on ice when not in the refrigerator.

(3) Exchanging the media in the channel with 1% BSA (DMEM) buffer:

The DMEM medium in the both of the reservoirs was carefully removed while keeping the channel filled with medium. For each channel, 300μL 1% BSA (DMEM) buffer was prepared. 160 μL of the solution was added to one reservoir and aspirated from the other reservoir at the same time using a cell culture aspirator, being careful not aspirate the buffer in the channel. Then both of the reservoirs were refilled with 60μL 1% BSA (DMEM) buffer. The μ-slide with cells was then put into a refrigerator (4°C) and incubated for 5 minutes.

(4) Staining of the HeLa cells in the channels with EGFR primary antibody:

For each channel, 150μL 2 μg/mL EGFR primary antibody solution in cold 1% BSA (DMEM) buffer was prepared. After the 5 minute incubation at the end of step 3, the solution in the both of the reservoirs was carefully removed while keeping the channel still filled with buffer. 100 μL diluted EGFR primary antibody solution was added to one reservoir and aspirated from the other reservoir at the same time without aspirating the solution in the channel. Then both of the reservoirs were refilled with 25μL diluted EGFR primary antibody solution. The μ-slide with cells was then put into a refrigerator (4°C) and incubated for 30 minutes.

(5) Washing of the channels with DMEM:

After the 30 minute incubation, the μ-slide with cells placed on ice. The EGFR primary antibody solution in both reservoirs of each channel was carefully removed and the cells in the channel were washed 3 times. For each wash cycles, 160 μL fresh, cold DMEM buffer was added to one reservoir and aspirated from the other reservoir at the same time without aspirating the buffer in the channel. The cells were incubated on ice for 5 minutes after the secondary and third wash cycles.

(6) Staining of HeLa cells in the channels with biotinylated secondary antibody:

The biotinylated secondary antibody stock was diluted 500-fold using cold 1% BSA (DMEM) buffer. 150μL of this solution was made for each channel. 100 μL diluted biotinylated secondary antibody solution was added to one reservoir and aspirated from the other reservoir at the same time without aspirating the solution in the channel. Then both of the reservoirs were refilled with 25μL diluted biotinylated secondary antibody solution. The μ-slide with cells was put into a refrigerator (4°C) and incubated for 30 minutes. The channels were then washed with DMEM as described in step 5 to remove excess biotinylated secondary antibody.

(7) Coating of the HeLa cells in the channels with Alexa 488-labeled streptavidin:

For each channel, 150μL 3 μg/mL Alexa 488-labeled streptavidin solution in cold 1% BSA (DMEM) buffer was prepared. 100 μL diluted Alexa 488-labeled streptavidin solution was added to one reservoir and aspirated from the other reservoir at the same time without aspirating the solution in the channel. Then both of the reservoirs were refilled with 25 μL diluted Alexa 488 labeled streptavidin solution. The μ-slide with cell was put into a refrigerator (4°C) and incubated for 30 minutes. After this 30 minute incubation, the channels were each washed with DMEM as described in step 5 to remove excess Alexa 488-labeled streptavidin.

(8) Coating of the HeLa cells in the channels with BDC tag:

150μL 1 μM BDC tag solution in cold 1% BSA (DMEM) buffer was prepared. 100 μL of this diluted BDC tag solution was added to one reservoir and aspirated from the other reservoir at the same time without aspirating the solution in the channel. Then both of the reservoirs were refilled with 25μL BDC tag solution. The μ-slide with cells was transferred to a refrigerator (4°C) and incubated for 30 minutes. After this incubation, each channel was washed with DMEM as described in step 5 to remove excess BDC tag.

(9) Attachment seeded nanotubes to HeLa cells:

PEG-coated DNA seeded nanotubes were diluted 1-fold by cold 1% BSA in DMEM-12mM $MgSO_4$ buffer. 220μL of this solution was prepared for each channel. 100 μL diluted seeded nanotube solution was added to one reservoir and aspirated from the other reservoir at the same time, using a cell culture aspirator, while being careful to not aspirate the solution in the channel. Both of the reservoirs were then refilled with 60μL diluted seeded nanotube solution. The μ-slide with cells was transferred to a refrigerator (4°C) and incubated for 2 hours. To prevent the seeded nanotubes from

settling during the incubation, 100μL seeded nanotube solution was carefully removed from one reservoir and added to the other 3 times every 30 minutes.

After the 2 hour incubation, the channel was washed with DMEM-12mM $MgSO_4$ as described in step 5 to remove the excess seeded nanotubes. Then the sample was ready for experiments in which fluid shear stress as applied within the fluid cell.

Supplementary Note S40: Characterizing the responses of seeded nanotubes anchored on HeLa cell membrane to shear stress induced by laminar fluid flow

HeLa cells were seeded in the channel of a μ-slide channel and PEG-coated 6nt seeded nanotubes were attached to EGFR receptors on HeLa cells using the EGFR AMDA protocol as described in Supplementary Note 39. DMEM-12mM $MgSO_4$ buffer was used as a flow perfusate. A series of flow rates in order from lowest to highest were applied to the cells as follows: 0, 0.03, 0.06, 0.12, 0.24, 0.36, 0.48, 0.6, and 0.72 mL per min. A spinning disk confocal microscope was used to capture images of the seeded nanotubes while flow was applied at the tops of cells. Here we defined the section of the cell farthest from the substrate in a stack of images captured usng the spinning disk confocal microscope in the cell labeling channel (Alexa488) as the cell top. The nanotubes attached to this part of the cell were imaged to measure shear stress. Images were taken every 5 seconds for 145 seconds and 30 images were taken for each flow rate.

Supplementary Note S41: Measuring the total angles of rotation of nanotubes under different amounts of shear stress induced by laminar fluid flow

At each time point for a given flow rate, an x-y plane image of nanotubes was captured using a spinning disk confocal microscope. The seeds and most of a nanotube remained in focus in a given x-y plane when flow was applied both when nanotubes were attached to glass and when nanotubes were attached to cells. These micrographs were captured in the same location, so that the orientations of the same set of nanotubes were tracked over time. For each flow rate studied for nanotubes attached to glass and on the surfaces of cells, the orientations of at least 15 nanotubes were measured.

The main criteria used for selecting a nanotube to be analyzed were that a nanotube be a) longer than 2 μm and b) not evidently sticking to a nearby surface or cell membrane. Nanotubes that overlapped with others or whose anchor locations moved during imaging were also excluded. We selected nanotubes longer than 2 μm because short nanotubes appeared as dots in X-Y projection images (*i.e.* their lengths and widths were commensurate in size), making it hard to measure their total rotation angles.

To measure the orientation of a given nanotube, the micrographs for each time point was cropped so that it contained only one of the tracked nanotubes. A Gaussian blur filter (radius:1.00) was applied in ImageJ to each of the 30 images of the nanotubes to reduce the image background. The resulting 30 images were then summed to produce a maximum time projection in ImageJ that showed all of the orientations of a given nanotube over time. Because the position of the nanotube seed was fixed, this set of overlaid images has a shape reminiscent of the sector of a circle whose angle of extent represented the size of the angular range traversed by the nanotubes in each of the images in the time series. The angle of this section, Φ, was termed the total angle of nanotube rotation. Φ was measured manually for each nanotube by measuring 1) the angle between the Y-axis and the lower edge of the sector Φ_{lower}, and 2) the angle between the -Y axis and the upper edge of the sector Φ_{upper}. The total angle of nanotube rotation is then determined using the formula:

$$\Phi = \Phi_{lower} + \Phi_{upper} - 180° \qquad (4.35)$$

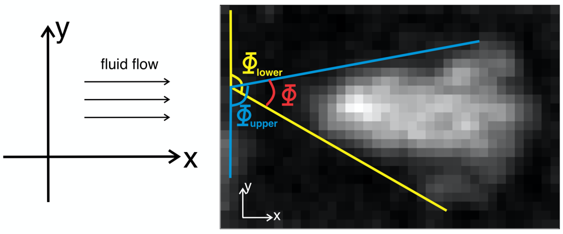

Supplementary Figure S25: Measurement of the total angle of nanotube rotation Φ. Left, schematic showing the direction of flow in the x-y plane of the micrographs collected of a nanotube. Right, examples of measuring Φ_{lower} and Φ_{upper} in an example maximum time projection image.

Section 5. Growing nanotubes on living cells by nanotube end-to-end joining

Supplementary Note S42: 6nt seeded nanotube end-to-end joining in solution

Nanotubes were grown from seed A and from seed B in two batches and then combined. When combined, the free ends of the nanotubes can join end-to-end. To visualize joined nanotubes, the nanotube monomers in the two batches were labelled with two different colors and the seeds were also labeled with different colors. For A seeded nanotubes, the seeds were labeled with atto488 and the nanotubes with atto647, for B seeded nanotubes, the seeds were labeled with atto647 and the nanotubes with Cy3. The experiment was performed as described below:

(1) Preparation of nanotubes grown from seed A:

To prepare these seeded nanotubes, Atto488 labeled nanotube seeds of type A that had 36 BDC' strands attached were first prepared. The reagents in Supplementary Table S18 were combined and then annealed as described in Supplementary Note S1 step 2. The nanotubes were purified and their concentration measured as described in Supplementary Note S1 step 3. The seeds were adjusted to 0.4nM by diluting them with 1x TAE-Mg^{2+} buffer.

Supplementary Table S18: 6nt nanotube seed A with 36 BDC tag attachment sites (Atto488 labeled)

Seed Assembly Mixture	Final desired concentration (nM or fold)	Stock concentration (nM or fold)	To add (µl)
H_2O	--	--	71.6 µl
TAE-Mg^{2+} buffer	1x	10x	10 µl
Seed staple strands mix	250nM	4167nM	6 µl
Seed A adapter strands mix (6nt)	100nM	4167nM	2.4 µl
M13mp18 scaffold strand	5nM	100nM	5 µl
biotin left attachment strands mix	20nM	1000nM	2 µl
30 Biotin attachment strands on unused M13 segment	10nM	1000nM	1 µl
66Seed labeling attachment strands mix	10nM	1000nM	1 µl
Labeling strand ATTO488	1000nM	100000nM	1 µl
Total			100 µl

The nanotube seeds assembled using this recipe had 6 BDC' strands (BDC tag attachment sites) at the left end of each seed's barrel (as illustrated) and an additional 30 BDC' strands (BDC tag attachment sites) in the middle of the unused section of each seed's M13 scaffold. These seeds were labeled with atto488 and nanotubes grow from the right sides of the seeds.

The seeded nanotubes were then assembled using the method described in Supplementary Note S7.2 except that atto647 labeled 6nt monomer (Supplementary Figure S35) were used in place of Cy3 labeled 6nt monomers. 3 aliquots of 19.7µL

nanotube monomer mix was prepared as described containing the reagents in Supplementary Table S19. They were annealed using the protocol in Supplementary Note S1 step 2; when the temperature reached 37°C, 0.3µL of the PEG coated seeds A prepared above (0.4nM) were added to each aliquot. All of samples were then incubated at 37°C for 24 hours.

Supplementary Table S19: Recipe for 6nt monomer (atto647 labeled) without PEG

Monomer mix	Final desired concentration (nM or fold)	Stock concentration (nM or fold)	To add (µl)
H_2O			14.7µl
TAE-Mg^{2+} buffer	1x	10x	2µl
Full 6nt monomer strands (atto647)	150nM	1000nM	3µl
Total			19.7µl

Full 6nt monomer strands (atto647): A mixture containing the same monomer strands as *Full 6nt monomer strands (Cy3),* except that the SEs_3-5'Cy3 was omitted and strands SEs_3-5'ATTO647 were added to the mixture each at concentration 1 µM (see Supplementary Figure S35).

(2) Preparation of nanotubes grown from seeds of type B:

Atto647 labeled nanotube seeds of type B with 36 BDC' strands were prepared by combining the reagents in Supplementary Table S14 in Supplementary Note S26, then annealing the mixture as described in Supplementary Note S1 step 2. The seeds were purified and their concentration measured as described in Supplementary Note S1 step 3. The concentration of the seeds was adjusted to 0.4nM by diluting them with 1x TAE-Mg^{2+} buffer.

3 aliquots of 19.7µL of monomer mix (where the monomers were labeled with Cy3) were prepared by combining the reagents in Supplementary Table S4. They were annealed as described in Supplementary Note S1 step 2. When the temperature reached 37°C, 0.3µL the PEG coated seeds of type B prepared above (0.4nM) were added to each aliquot of 19.7 µL monomers. All of the samples were then incubated at 37°C for 24 hours.

(3) Nanotube end-to-end joining in solution:

Both kinds of nanotubes were prepared so as to finishing their incubations at the same time. At this point, equal volumes (10 µL) of both types of seeded nanotubes were mixed in a test tube and incubated at 37°C. Samples of the resulting seeded nanotube mixture were imaged under epi-fluorescence microscope after 3.5 hours and after 26 hours of incubation. Images of 10 random locations were captured at each time point.

(4) Quantification of the fraction of nanotubes that had joined end-to-end:

In each captured image the total number of nanotubes and nanotube segments (one part of a joined nanotube of a single color) of each color was counted manually as

$N_{Cy3\ NT}$ and $N_{atto647\ NT}$ respectively. Both seeded and unseeded nanotubes were counted. Nanotubes only partially in the image were not counted. Nanotubes that were tangled together and thus hard to distinguish were also not counted.

The number of Cy3 labeled nanotubes that were end-to-end joined to an atto647 labeled nanotube ($N_{Cy3\text{-}atto647\ NT}$) were counted manually in merged images that showed both Cy3-labeled and atto647-labeled nanotubes in a single merged image. (The images were merged in ImageJ).

The fraction of Cy3 labeled nanotubes that end-to-end joined with atto647 labeled nanotubes at each location was then calculated as

$$Y_{Cy3} = \frac{N_{Cy3-atto647\ NT}}{N_{Cy3\ NT}} \tag{5.1}$$

The number of atto647 labeled nanotubes that end-to-end joined with a Cy3 labeled nanotube ($N_{atto647\text{-}Cy3\ NT}$) were also counted manually in the merged images.

The fraction of atto647 labeled nanotube been end-to-end joined with atto647 labeled nanotubes at each location was then calculated as

$$Y_{atto647} = \frac{N_{atto647-Cy3\ NT}}{N_{atto647\ NT}} \tag{5.2}$$

Y_{Cy3} and $Y_{atto647}$ were both calculated for each of the micrographs captured at each time point. The averages of these values over all micrographs for each time point were taken as the fraction of nanotubes that had end-to-end joined rate at each of the time points.

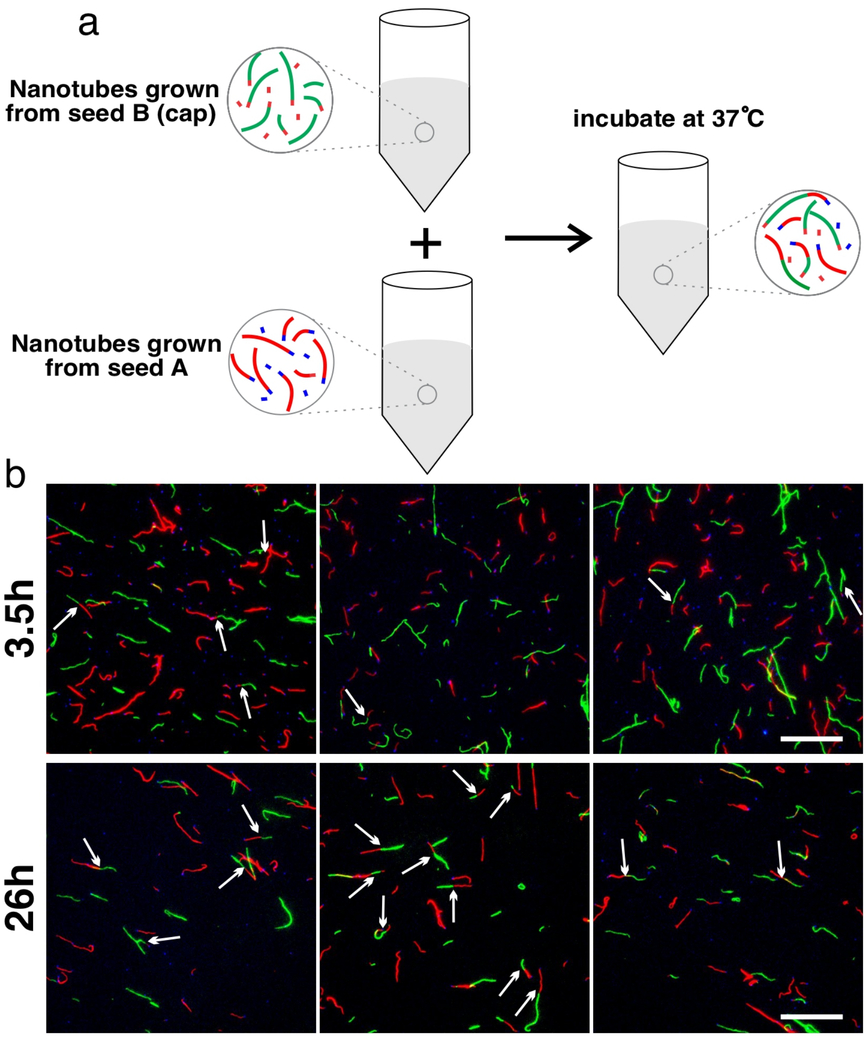

Supplementary Figure S26: Measuring the rate of end-to-end joining of DNA nanotubes in solution. **(a)** Experimental design. Nanotubes are grown from seeds of type A and from seeds of type B in two batches and then combined to allow them to undergo end-to-end joining. To make it possible to visualize which nanotubes joined, the nanotube monomers in the two batches were labelled with different dyes and the two types of seeds were also labeled with different dyes. The joining experiment was performed as described in Supplementary Note S42. Nanotubes grown from seed of type A and nanotubes grown from seed of type B were prepared as described in Supplementary Note S37 step1. **(b)** Example micrographs of nanotubes after 3.5 hours of incubation (upper panel) and 26 hours of incubation (lower panel). Seeds of type A were labeled with atto488 (blue) and nanotubes grown from seed of type A were labeled with atto647 (red). Seeds of type B were labeled with atto647 (red) and nanotubes grown from seeds of type B were labeled with Cy3 (green). Scale bars 10 µm. 7±2% of atto647 labeled nanotubes were joined to Cy3 labeled nanotubes after being incubated at 37°C for 3.5h. This percentage increased to 30±6% after incubation for 26h. 10±3% of Cy3 labeled nanotubes were joined to atto647 labeled nanotubes after being incubated at 37°C for 3.5h. This percentage increased to 30±6% after incubation for 26h.

Supplementary Note S43: Assembly of 4PEG nanotubes for nanotube joining

43.1. Assembly of 4PEG seeds and capping seeds

The components for unlabeled 4PEG nanotube seeds of type A were mixed according to the proportions given in Supplementary Table S20. The components for 4PEG nanotube seeds of type A labeled with atto647 were mixed according to the proportions given in Supplementary Table S21. The components for unlabeled 4PEG nanotube seeds of type B were mixed according to the proportions given in Supplementary Table S22. The seeds were then annealed from 90°C to 20°C in a thermocycler (Eppendorf Mastercycler) using the following annealing schedule, as described in Agrawal[3]:

- 5 mins at 90°C
- 90°C to 45°C at 1°C/min
- 45°C for 60 mins
- 45°C to 20°C at 1°C/10mins
- 20°C hold until sample retrieval

Then the seeds were purified as described in Supplementary Note S1 step 3. The process for determining the concentrations of the seeds used elsewhere in this work required that the seeds be fluorescently labeled. To determine the concentrations of the unlabeled seeds, after their purification, 1µL of 10x TAE-Mg^{2+} buffer was added to 9µL of 1 µM labeling strand and mixed well. This solution was then added to 10 µL of unlabeled seeds, incubated at room temperature for 10 minutes. The concentrations of these seeds were then measured as described in Supplementary Note S1 step 3. The seeds were coated with PEG as described in Supplementary Note S3.

Supplementary Table S20: Recipe for unlabeled seeds of type A for 4PEG nanotubes with 36 biotin attachment sites

Seed Assembly Mixture	Final desired concentration (nM or fold)	Stock concentration (nM or fold)	To add (µl)
H_2O	--	--	72.6 µl
TAE-Mg^{2+} buffer	1x	10x	10 µl
Seed staple strands mix	250nM	4167nM	6 µl
4nt anchored seed adapter strands mix	100nM	4167nM	2.4 µl
M13mp18 scaffold strand	5nM	100nM	5 µl
Extended biotin right attachment strands mix	20nm	1000nM	2 µl
30 Biotin attachment strands on unused M13 segment	10nM	1000nM	1 µl
66 Seed labeling attachment strands mix	10nM	1000nM	1 µl
Total			100 µl

4nt anchored seed adapter strands mix: A mixture containing all 24 Seed B adapter

strands for 4nt nanotubes (Supplementary Note 50.3 and Supplementary Figure S46) in water each at 4176nM (100µM/24).

Supplementary Table S21: Recipe for Atto647 labeled seeds of type A for 4PEG nanotubes with 36 biotin attachment sites.

Seed Assembly Mixture	Final desired concentration (nM or fold)	Stock concentration (nM or fold)	To add (µl)
H_2O	--	--	71.6 µl
TAE-Mg^{2+} buffer	1x	10x	10 µl
Seed staple strands mix	250nM	4167nM	6 µl
4nt anchored seed adapter strands mix	100nM	4167nM	2.4 µl
M13mp18 scaffold strand	5nM	100nM	5 µl
Extended biotin right attachment strands mix	20nm	1000nM	2 µl
30 Biotin attachment strands on unused M13 segment	10nM	1000nM	1 µl
66 Seed labeling attachment strands mix	10nM	1000nM	1 µl
Labeling strand ATTO647	1000nM	100000nM	1 µl
Total			100 µl

Supplementary Table S22: Recipe for unlabeled seeds of type B for 4PEG nanotubes with no biotin attachment sites

Seed Assembly Mixture	Final desired concentration (nM or fold)	Stock concentration (nM or fold)	To add (µl)
H_2O	--	--	75.6 µl
TAE-Mg^{2+} buffer	1x	10x	10 µl
Seed staple strands mix	250nM	4167nM	6 µl
4nt capped seed adapter strands mix	100nM	4167nM	2.4 µl
M13mp18 scaffold strand	5nM	100nM	5 µl
99Seed labeling attachment strands mix	10nM	1000nM	1 µl
Total			100 µl

4nt capped seed adapter strands mix: A mixture containing all 24 Seed A adapter strands for 4nt nanotubes (see Supplementary Note 50.3 and Supplementary Figure S45) in water each at concentration 4176nM (100µM/24).

43.2. Protocol for growing 4PEG nanotubes

The architectures of the two types of monomers that make up 4PEG nanotubes tubes are shown in Supplementary Figure S36b. The central strands of each type of monomer (REd_3-5'Cy3-3'amine and SEd_3-5'Cy3-3'amine) were modified with PEG following the procedures used to modify the central strand of the 6nt nanotube monomer as described in Supplementary Note 2. The resulting strands were termed REd_3_PEG and SEd_3_PEG. We used 180 nM of each type of monomer because we found that nanotubes did not form when using lower monomer concentrations.

To prepare 4PEG nanotubes, the reagents for nanotubes given in Supplementary Table S23 were combined and annealed from 90°C to 20°C using the same annealing schedule used to anneal 4PEG seeds. When the solution reached 20°C, 2 µl of PEG coated seed solution (0.4nM) was added to the 19.7 µl of monomer solution. The mixture was then incubated at 20°C for at least another 3 days to let the nanotubes grow from the seeds.

Supplementary Table S23: Recipe for 4nt monomers with PEG

Monomer solution	Final desired concentration (nM or fold)	Stock concentration (nM or fold)	To add (µl)
H_2O			13.38 µl
TAE-Mg^{2+} buffer	1x	10x	2 µl
REd_3_PEG	180 nM	10000 nM	0.36 µl
SEd_3_PEG	180 nM	10000 nM	0.36 µl
The remaining 4nt monomer strands (1,2,4,5)	180 nM	1000 nM	3.6 µl
Total			19.7 µl

The remaining 4nt monomer strands (1,2,4,5): A mixture containing the 8 SEd-REd monomer strands SEd_1, SEd_5, REd_1, and REd_5 (see Supplementary Figure S36b) in water each at a concentration of 1µM, and the SEd-REd monomer strands SEd_2, SEd_4, REd_2, REd_4 each at a concentration of 2 µM.

REd_3_PEG: the strand REd_3-5'Cy3-3'amine (Supplementary Figure S36b) conjugated with PEG in water.

SEd_3_PEG: The strand SEd_3-5'Cy3-3'amine (Supplementary Figure S36b) conjugated with PEG in water. This is actually the SEs_3_PEG since the SEd_3-5'Cy3-3'amine has the same sequence as SEs_3-5'Cy3-3'amine (Supplementary Note S49.2).

Each of the seeds prepared in 43.1 were used to grow seeded nanotubes using the method described above. To check that nanotubes grew from the unlabeled seeds, labeling strands were added after seeded nanotubes grown. For unlabeled seeds of type A, 0.1µL Labeling strand Atto647 (1µM) was added to 5µL of seeded nanotube solution and then incubated for 10 minutes at 20°C. After incubation, the anchored solution was diluted 11-fold with 1xTAE-Mg^{2+} buffer and them imaged under a fluorescent microscope (Supplementary Figure S27a). For the nanotubes grown from

unlabeled seeds of type B, 0.1µL Labeling strand Atto488 (1µM) was added to 5µL of nanotube solution and incubated 10 minutes at 20°C before being diluted and imaged under a fluorescent microscope (Supplementary Figure S27b).

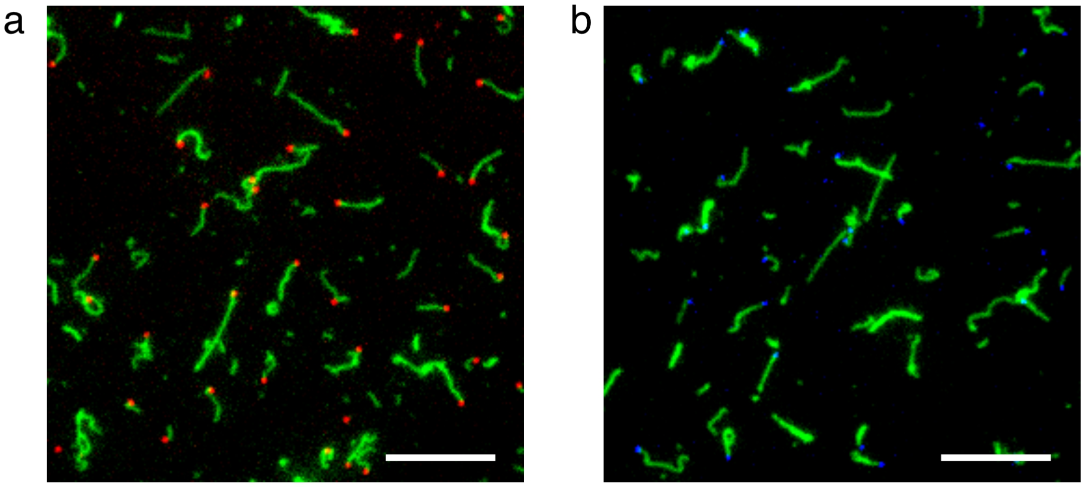

Supplementary Figure S27: Images of 4PEG nanotubes grow from seeds of type A (a) and 4PEG seeded nanotubes grown from seeds of type B (b). The seeds were not labeled with fluorescent dye during seed assembly or nanotube growth. Seeds were visualized by adding fluorescent labeling strands as described in 43.2 just before imaging. Red: atto647, green: Cy3, blue: atto488. Scale bar: 10µm. This experiment was repeated more than three times independently with similar results.

Supplementary Note S44: Assembly of inactive monomers used as nano-glue

We hypothesized that end-to-end joining of nanotubes did not occur because the monomer detachment rate was very low, allowing rough facets or facets with defective monomers that cannot join to persist (Supplementary Figure S28). As a result, 4PEG seeded nanotubes were used for joining because these monomers with higher attachment and detachment rate than the 6nt seeded nanotubes monomers. It was found that the capped seeded nanotubes could join to the ends of anchored seeded nanotubes with high yield in the presence of annealed monomers at high concentration (*i.e.* 150nM), because these monomers could serve as "glue" to fill in the gaps between rough facets. To prevent these monomers at higher concentration forming unseeded nanotubes during the annealing process which would deplete the free monomers, here we used the 4nt inactivated monomers (Supplementary Figure S38). One sticky end of the inactive monomer was blocked. These inactivated monomers could be annealed at high concentrations without forming unseeded nanotubes. An active strand could then be added to expose the blocked sticky ends to form functional monomers that can participate in nanotube end-to-end joining.

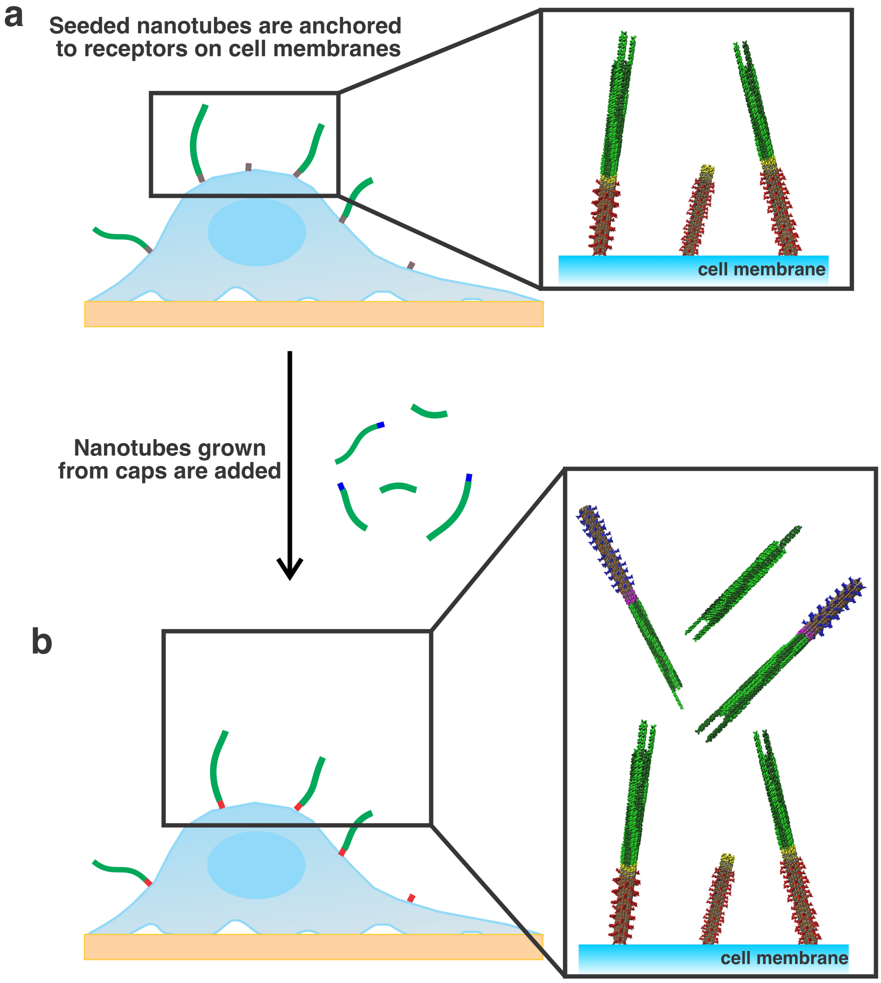

Supplementary Figure S28: Schematic of end-to-end joining of seeded nanotubes to nanotubes anchored to the cell membrane.

The 4nt inactive monomers were prepared by mixing the reagents listed in the recipes

below (Supplementary Tables S24 and S25) and annealed from 90°C to 20°C using the same annealing schedule that was used to anneal 4nt seeded nanotubes.[3]

Supplementary Table S24: Recipe for Atto488 labeled inactive monomers

Monomer solution	Final desired concentration (nM or fold)	Stock concentration (nM or fold)	To add (µl)
H_2O			0µl
TAE-Mg^{2+} buffer	1x	10x	5µl
Inactive monomer mix (atto488)	900nM	1000nM	45µl
Total			50µl

Inactive monomer mix (atto488): A mixture containing monomer strands including all of the SEd tile strands-- SEd_1, SEd_3-5'ATTO488 and SEd_5 (Supplementary Figure S37a), the REd tile strands--REd_1, REd_3-5'ATTO488, the strands HS_REd_4bpD1_5' and HS_REd_4bpD1_3' (Supplementary Figure S38a) in water each at a concentration of 1µM and the strands SEd_2, SEd_4, REd_2 and REd_4, each at a concentration of 2 µM.

Supplementary Table S25: Recipe for Atto647 labeled inactive monomers

Monomer solution	Final desired concentration (nM or fold)	Stock concentration (nM or fold)	To add (µl)
H_2O			0µl
TAE-Mg^{2+} buffer	1x	10x	5µl
Inactive monomer mix (atto647)	900nM	1000nM	45µl
Total			50µl

Inactive monomer mix (atto647): A mixture containing the same monomer strands as Inactive monomer mix (atto488), except that the SEd_3-5'ATTO488 and REd_3-5'ATTO488 were omitted and strands SEd_3-5'ATTO647 and REd_3-5'ATTO647 were in the mixture each at concentration 1 µM (see Supplementary Figure S37b and S38b).

Supplementary Note S45: Measuring the joining rate between 4PEG nanotubes anchored to a glass surface and 4PEG nanotubes in solution in the presence of additional monomers (nanoglue)

(1) Preparation of the anchored and capped 4PEG nanotubes

4PEG unlabeled anchored nanotube seeds with 36 biotin attachment sites were prepared by combined the reagents in Supplementary Table S20. 4PEG unlabeled capped nanotube seeds with no biotin attachment sites were prepared by combined the reagents in Supplementary Table S22. Both of these solutions were annealed from 90°C to 20°C as described in Supplementary Note S43, and purified as in Supplementary Note S1 step 3. The concentrations of both unlabeled anchored and capped seeds were determined as described in Supplementary Note S43 and then coated with PEG as described in Supplementary Note S3.

Cy3 labeled 4PEG anchored and capped seeded nanotubes were then prepared using these seeds as described in Supplementary Note S43.2.

(2) Preparation of the Atto488-labeled inactive monomers:

The Atto488-labeled inactive monomer solution contained atto488 labeled 4nt SEd tile (Supplementary Figure S38a) and Atto488 labeled REd inactive tile (Supplementary Figure S38a). 50 µl of a solution containing 900 nM Atto488-labeled inactive monomers in TAE-Mg^{2+} buffer was prepared by mixing the reagents listed in Supplementary Table S24 and annealing the resulting solution from 90°C to 20°C as described in Supplementary Note S43.

(3) Attachment of anchored 4PEG nanotubes to the glass bottom of the µ slide channel:

The glass bottom of a channel in a 6-channel µ slide were treated as described in Supplementary Note S36. The 4PEG anchored seeded nanotubes were attached to the glass bottom of the treated channel using the method described in Supplementary Note S37.

(4) Addition of activated monomers to capped seeded nanotubes:

0.27 µL of 100 µM activation strand in water (Supplementary Figure S38a) was added to 30 µL atto488-labeled inactive monomer prepared in step 2 and mixed well by pipette to activate the monomers. This activated monomer solution was then immediately mixed with a solution of 4PEG coated, capped nanotubes by combining the solutions listed in Supplementary Table S26.

Supplementary Table S26: Mixture of capped seeded nanotubes and nanotube monomers for end-to-end joining

Nanotubes and monomers	Final desired concentration (nM or fold)	Stock concentration (nM or fold)	To add (µl)
1%BSA(DMEM)-12mM $MgSO_4$			65µl
capped seeded nanotube			60µl

Atto488-labeled inactive monomers	150nM	900nM	25μl
Total			150μl

(5) Nanotube joining:

100 μL of the mixture prepared in step 4 was immediately added to one reservoir of the channel and aspirated from the other reservoir at the same time, without aspirating the solution in the channel. Then both of the reservoirs were refilled with 25μL of the mixture prepared in step 4. The sample was then covered with foil and incubated on a bench (about 19~21°C) for 4 hours.

(6) Washing the channel:

After incubation, the glass bottom channel was washed with 1x TAE-Mg^{2+} buffer three times before imaging to remove free nanotubes not joined and the extra activated monomers. For each wash cycle, 160 μL TAE-Mg^{2+} buffer was pipetted into one reservoir of the channel and aspirates from the other reservoir at the same time without aspirating the solution in the channel. Then both of the reservoirs were refilled with 25μL TAE-Mg^{2+} buffer.

(6) Imaging the nanotube joining on glass surface:

After the joining reaction, the nanotubes anchored to the glass surface were imaged under a spinning disk confocal microscope. To clearly visualize the structure of the nanotubes (*i.e.* the different colors along the nanotube that would indicate joining and monomer gluing), a gentle fluid flow was applied to stretch the nanotubes as described in the Supplementary Note S48. The fluid flow rate was 0.18 mL/min which should induce a shear stress of 0.32 dyn/$cm^{2.}$ 1x TAE-Mg^{2+} buffer was used as a flow perfusate.

Since the nanotubes were labeled with Cy3 and the activated monomers were labeled with atto488, a dual camera setup was used to simultaneously capture images in the Cy3 and atto488 channels on the confocal microscope to avoid misalignment between the image channels. Images were taken every 2 seconds for 18 seconds (10 frames) at each of 11 random locations on the μ slide surface.

(7) Quantification of the fraction of seeded nanotube on the surface that underwent end-to-end joining:

The fraction of seeded nanotubes that underwent end-to-end joining on glass surface when additional nanoglue monomers were added to the reaction was quantified by measuring the fraction of nanotubes that contained multiple fragments of Cy3-labeled nanotubes separated by visible atto488 segments.

The fluorescence micrographs of both color nanotubes at each location were merged together in ImageJ (see an example in Supplementary Figure S28). The anchored and capped nanotubes both labeled with Cy3 were colored green and the atto488 labeled activated monomer (nanoglue) were blue in these merged images. The total number of anchored nanotubes ($N_{anchored\ NT}$) was counted manually in these merged images.

Nanotubes that crossing the edge of image edges and nanotubes that were tangled together or overlaid and hard to distinguish were not counted. Since the anchored nanotubes were labeled with Cy3 (green), all the nanotubes started with green segment along the fluid flow direction were counted to get the $N_{anchored\ NT}$.

The number of anchored nanotubes that underwent end-to-end joining or grew ($N_{joined\ anchored\ NT}$) was counted by manually counting all anchored nanotubes with both green and blue segments.

The fraction of joined anchored nanotubes at each location was then calculated as:

$$Y_{joined\ anchored\ NT} = \frac{N_{joined\ anchored\ NT}}{N_{anchored\ NT}} \tag{5.3}$$

$Y_{joined\ anchored\ NT}$ was calculated for each of the 11 random locations.

(8) Measurement of the lengths of seeded nanotubes anchored to a glass surface after end-to-end joining:

The methods of measuring the lengths of the end-to-end joined seeded nanotubes after joining were similar to the methods described in Supplementary Note 7.3. The time lapse fluorescence micrographs of both color nanotubes at each location took in Step (6) were merged together in ImageJ. In the merged timelapse images at a given location, to maximize the accuracy of the length determination process, the contrast of each color in the merged image was enhanced using linear histogram stretching with ImageJ software. Since the nanotubes were moving under the fluid flow and thus sometimes moved in and out of focus, the timelapse image showing the longest contour image was selected for measurement. The length of an end-to-end joined nanotube was measured by manually drawing segmented lines along the nanotube curves, from the start tip to the end tip of nanotube, in ImageJ software. Nanotubes whose measured lengths were less than 0.5µm (about 3 pixels) were counted as having length 0. Nanotubes that were overlapping or were not entirely within the field of view were both excluded.

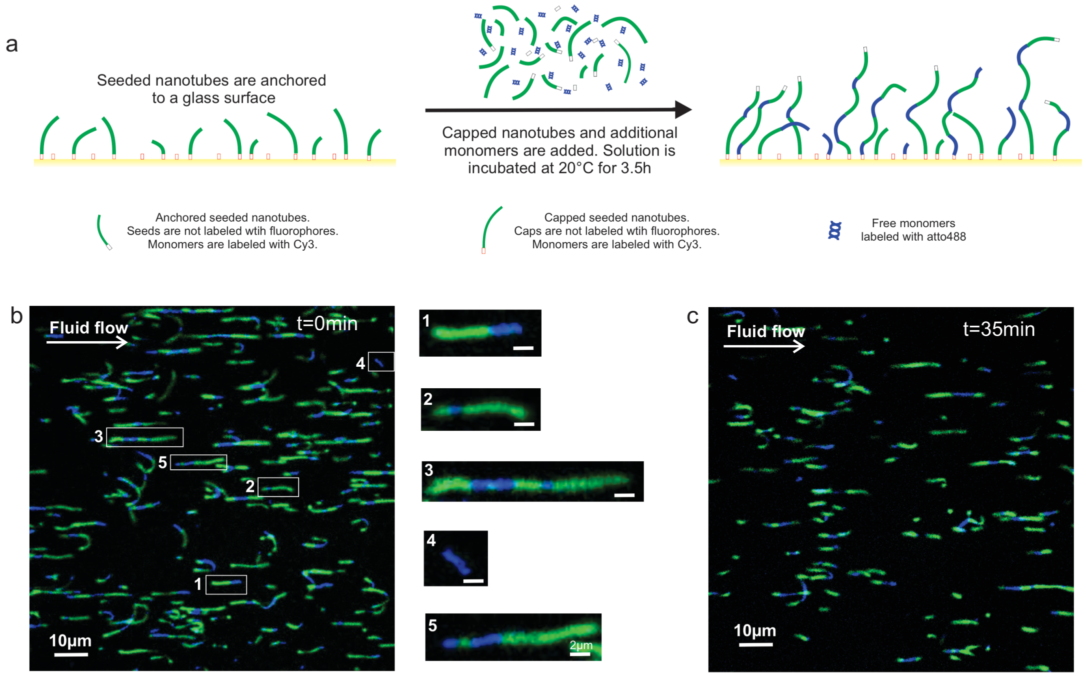

Supplementary Figure S29: End-to-end joining of DNA nanotubes anchored to a glass surface to nanotubes in solution. Experiments were performed as described in Supplementary Note S45. (a) Schematic of the process used to measure end-to-end joining of 4PEG nanotubes in the presence of extra monomers. (b) Confocal micrograph of nanotubes after joining under a gentle fluid flow (shear stress 0.32dyn/cm^2) at 20 °C. 86±3% anchored nanotubes were joined to capped nanotubes after incubation with capped nanotubes and 150nM additional monomers at 20ºC for 4 hours. Scale bars of zoomed-in images: 2μm. (c) Confocal micrograph of end-to-end joined nanotubes after applying fluid flow (shear stress 0.32dyn/cm^2) for 35 minutes at 20 °C. This experiment was repeated more than three times independently with similar results.

Supplementary Note S46: Joining of nanotubes anchored to the HeLa cell surface

(1) Preparation of the anchored and capped 4PEG nanotubes:

Both the anchored and capped 4PEG nanotubes were prepared as described in step 1 of Supplementary Note S45.

(2) Preparation of the Atto647-labeled inactive monomers:

The Atto647-labeled inactive monomers consisted of a mixture of atto647 labeled 4nt SEd tiles (Supplementary Figure S37b) and atto647 labeled REd inactive tiles (Supplementary Figure S38b). 50 µl of a solution containing 900 nM Atto647-labeled inactive monomers in TAE-Mg^{2+} buffer was prepared by mixing the reagents listed in Supplementary Table S25 and annealing the resulting solution from 90°C to 20°C as described in Supplementary Note S43.

(3) Attachment of the anchored seeded nanotubes to EGFR receptors on HeLa cells using EGFR AMDA in µ-slide channels:

The process of attaching the anchoted seeded nanotubes to EGFR receptors on HeLa cells using EGFR AMDA in µ-slide channels was performed as described in Supplementary Note S39, except that 3µg/mL neutravidin solution was added in step 7 instead of using Alexa 488-labeled streptavidin solution.

(4) Preparation of the mixture of activated monomer and the capped seeded nanotubes:

To activate the inactive monomers, 0.27 µL 100 µM activation strand in water was added to the 30µL 100µM Atto647 labeled inactive monomer solution prepared in step 2 and mixed well by pipette. Then 25 µL of this activated monomer solution was immediately mixed with 60µL 4PEG coated capped seeded nanotubes and 65µL 1% BSA (DMEM)-12.5mM buffer.

(5) Nanotube joining:

100 µL of the above mixture was immediately added to the channel using the method described in Supplementary Note S45, step 5. The sample was then covered with foil and incubated on a bench (about 19~21°C) for 4 hours. After incubation, the glass bottom channel was washed with DMEM-12mM $MgSO_4$ buffer three times in the manner described in Supplementary Note 45, step 6.

(6) Imaging the joined nanotubes on HeLa cells:

The sample was imaged under an epifluorescence microscope by using Andor SOLIS (Oxford Instruments) software, while a gentle fluid flow was applied using the method described in Supplementary Note S40. The fluid flow rate was 0.18mL/mL (shear stress 0.32 dyn/cm^2). DMEM-12mM $MgSO_4$ buffer was used as perfusate.

A series of 20 continuous images were captured each fluorescence channel. All the images from each channel were merged together to generate a series of 20 multicolor images such that the first image in the series was merged from the first of the three 20

single color image series and so on. The anchored and capped nanotubes labeled with Cy3 were color with green and the atto647 labeled activated monomer were red in this merged video (Supplementary Video S4). Since the nanotubes were moving during the period when the images were captured and the images from different channels were taken at different times, the nanotubes did not always appear connected in the merged images. Because the focal plane for imaging was at the slide surface, near the bottoms of the cells, the top of cell appeared blurry in these images.

(7) Quantifying the fraction of seeded nanotubes that underwent end-to-end joining on cell surface**:**

The methods used to quantify the fraction of seeded nanotubes that underwent end-to-end joining on the cell surface were essentially the same as those described in Supplementary Note S45 to quantify the fraction of seeded nanotubes that underwent end-to-end joining on a glass surface.

The total number of anchored nanotubes ($N_{anchored\ NT}$) was counted manually from Supplementary Video 4 by counting the number of nanotubes attached to the cell that started with a green segment; the presence of fluid flow made it easier to determine which segment this was. Nanotubes that were attached to the glass bottom of the channel, that crossed the edge of the video's frames or were overlaid with each other to the extent that they were hard to distinguish were all excluded. The number of anchored nanotubes that had undergone end-to-end joining ($N_{joined\ anchored\ NT}$) was also obtained by manually counting anchored nanotubes with both green and red segments. The fraction of anchored nanotubes joined at each location was calculated by Equation 5.3. 6 locations were analyzed using this procedure.

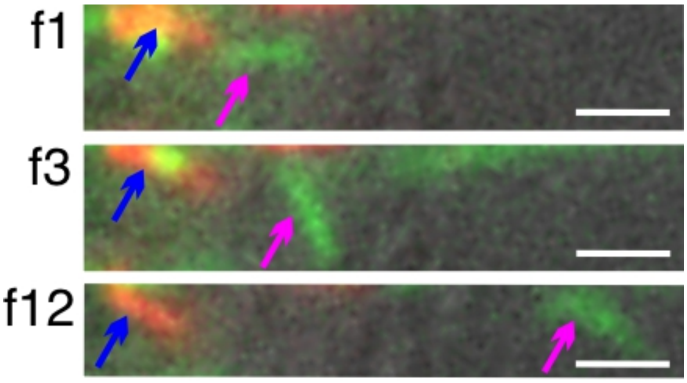

Supplementary Figure S30: Joined nanotubes can fracture in flow. Frames 1, 3 and 12 from a series of 30 images of the same nanotube captured in immediate succession. The blue and magenta arrows point to the locations of the red and green segments of a nanotube. The segments are connected in frame 1, but have disconnected by frame 3 and the green fragment is almost out of view by frame 12. The nanotube broke at the interface between the red and green segments. Scale bars 2.5μm.

(8) Measurement of the lengths of seeded nanotubes on the cell membrane afeter end-to-end joining:

The methods used to quantify the lengths of seeded nanotubes that underwent end-to-end joining on the cell surface were essentially the same as those described in Supplementary Note S45 used to quantify the fraction of seeded nanotubes that underwent end-to-end joining on a glass surface.

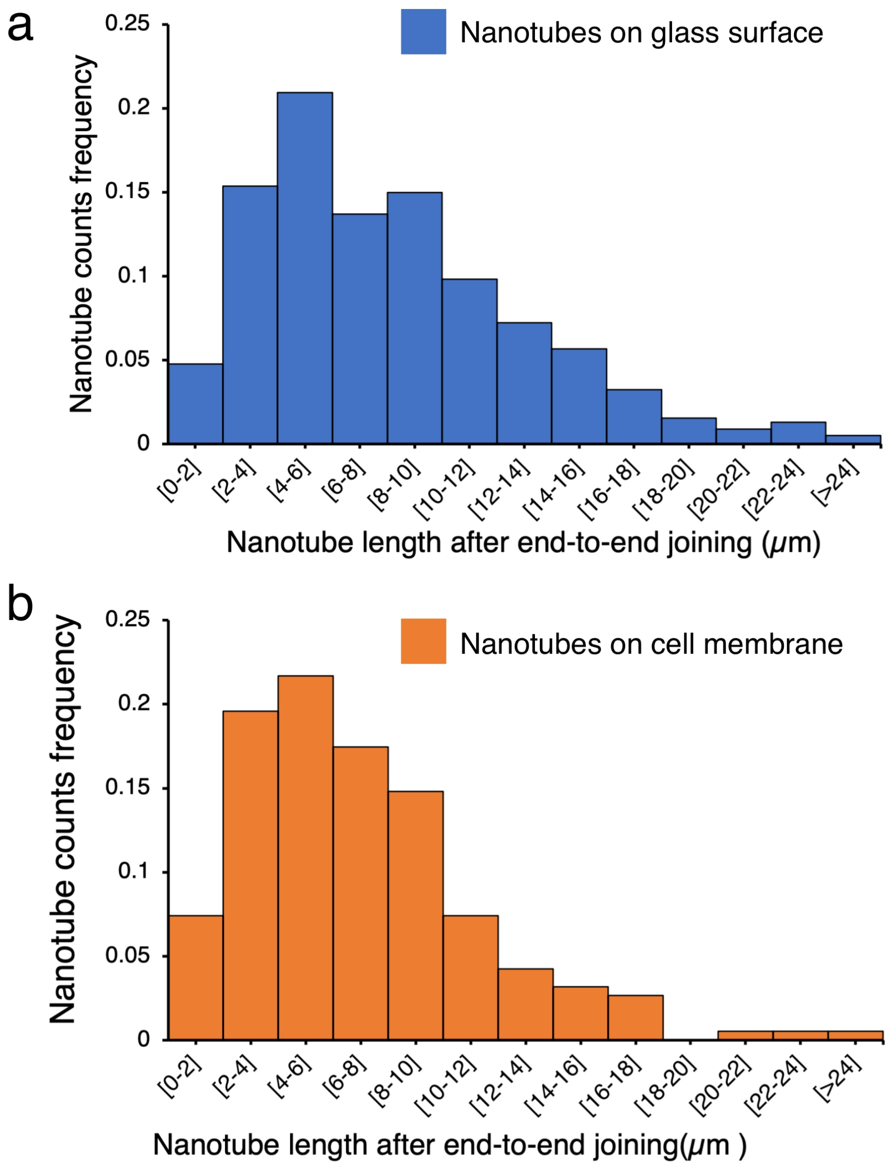

Supplementary Figure S31: Length distributions of seeded nanotubes after end-to-end joining. a. Histogram of the length distribution of 4PEG seeded nanotubes anchored on a glass surface after the nanotubes were incubated with capped nanotubes and 150 nM of additional monomers at 20°C for 3.5h (Supplementary Note S45). (N=774 nanotubes in eleven fields of view were measured). **b.** Histogram graph of length distribution of 4PEG seeded nanotubes anchored anchored on a cell membrane incubated with capped nanotubes and additional 150nM monomers at 20°C for 4h (Supplementary Note S46). (N=189 nanotubes in six fields of view were measured).

Supplementary Note S47: Visualizing nanotubes on the cell surface by adding methylcellulose to reduce thermal motion

The rate of thermal motion of nanotubes is reduced in a buffer with high viscosity. We added methylcellulose to cells with attached nanotubes to better visualize the structures of the joined nanotubes on the cell surface.

Before doing so, we first tested whether the addition of methylcellulose would break the nanotubes, using the experimental procedure described below. The seeded nanotubes were attached on the glass surface of the glass bottom dish and the buffer in the dish was exchanged to a buffer with added methylcellulose as follows:

(1) Seeded nanotube preparation:

The 4nt atto647 labeled anchored nanotube seeds with 36 biotin attachment sites were prepared by combining the reagents in Supplementary Table S21, annealing the mixture from 90°C to 20°C as described in Supplementary Note S43, purifying the seeds and measuring their concentration as in Supplementary Note S1 step 3, and then coating them with PEG as described in Supplementary Note S3.

Using these seeds, Cy3 labeled 4PEG anchored seeded nanotubes were prepared as described in Supplementary Note S43.2.

(2) Modifying the glass bottom dish with neutravidin:

To attach the seeded nanotubes to the glass surface, the glass bottom dish was treated by modifying the glass surface with a biotin-PEG-silane monolayer and neutravidin as described in Mohammed *et al*[1].

(3) Modifying the glass bottom dish with BDC tag:

The glass bottom of the dish was modified with BDC tag by adding 400 µL BDC tag (1 µM in 1xTAE-Mg^{2+} buffer) and incubating for 10min. The dish was then washed with 1xTAE-Mg^{2+} buffer 3 times to remove unattached BDC tag.

(4) Attachment of seeded nanotubes to the glass bottom dish:

400 µL of the PEG coated seeded nanotubes prepared in step 1 were diluted 1-fold by 1xTAE-Mg^{2+} buffer and this solution was then added to the dish and incubated for 10-20 minutes. After incubation, the dish was washed with 1xTAE-Mg^{2+} buffer 3 times to remove the unattached nanotubes. Then the seeded nanotubes attached on the glass bottom of the dish were imaged under an epi-fluorescence microscope. A series of 30 continuous images were taken for each fluorescence channel and all the images in each channel were merged together afterwards using the method described in Supplementary Note S46, step 6.

(5) Buffer exchange to introduce methylcellulose.

500 µL 0.6% methylcellulose solution was prepared by diluting 3% methylcellulose stock (HSC001) with Iscove's Modified Dulbecco's Medium (IMDM). Then additional $MgSO_4$ was added to adjust the Mg^{2+} to 12mM. The buffer in the dish was removed and replaced with this diluted methylcellulose solution containing 12mM

$MgSO_4$. The nanotubes in the dish were then imaged under an epi-fluorescence microscope; a series of 30 continuous images were taken in each fluorescence channel.

(6) Exchanging the buffer in the dish back to 1xTAE-Mg^{2+} buffer

Then the buffer in the dish was exchanged again so that the nanotubes were re-immersed in 1xTAE-Mg^{2+} buffer. The methylcellulose solution was slowly removed with a pipette and 500 µL fresh 1xTAE-Mg^{2+} buffer was added. The dish was washed by exchanging the 1xTAE-Mg^{2+} buffer 3 times to complete the removal of the methylcellulose in the dish. The seeded nanotubes were imaged under an epi-fluorescence microscope and a series of 30 continuous images were taken for each fluorescence channel.

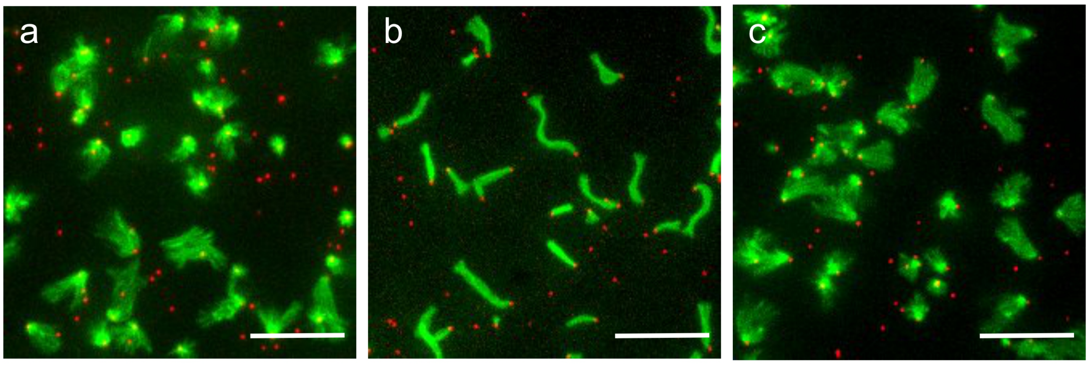

Supplementary Figure S32: The rate of thermal motion of seeded nanotubes anchored to a glass surface is reduced in 0.6% methylcellulose medium. A series of 30 continuous images of nanotubes anchored to a glass surface were captured to generate maximum time projection images in both methylcellulose containing medium and TAE-Mg^2 buffer. These images are the sum of 30 continuous captured images and thus show the nanotubes' paths and range of motion over the time needed to capture all of the component images. Maximum time projection image of seeded nanotubes (**a**) in 1x TAE-Mg^{2+} buffer, (**b**) after buffer was exchanged for 0.6% methylcellulose in IMDM with 12mM $MgSO_4$, (**c**) after the buffer was switched back to 1xTAE-Mg^{2+} from 0.6% methylcellulose in IMDM with 12mM $MgSO_4$ in (**b**). Scale bars 10µm. The extent of motion was reduced dramatically in methylcellulose and returned to a normal range after the methylcellulose was removed.

Supplementary Note S48: Visualizing joined nanotubes on the cell surface by adding methylcellulose to reduce the rates of nanotubes' thermal motion

We next used 0.6% methylcellulose solution to visualize nanotubes that had undergone end-to-end joining while anchored to cells.

(1) Seeded nanotube preparation:

4PEG anchored seeded nanotubes were prepared as described in Supplementary Note S47 step 1. 4PEG capped nanotubes were prepared as described in Supplementary Note S45 step 1.

(2) End-to-end joining of nanotubes anchored to HeLa cells:

HeLa cells with nanotubes attached to their EGFR receptors via EGFR AMDA were prepared in the channel of µ-slid and additional nanotubes were joined to the anchored nanotubes as described in Supplementary Note S46 steps 2-5 except that HeLa-GFP cells were used in place of HeLa cells in step 2 and the anchored nanotube seeds were labeled with atto647, *i.e.* they were the nanotubes prepared in step 1.

(3) Exchange of buffer to add methylcellulose:

After these cells were prepared, the buffer that the cells were incubated in was changed to 0.6% methylcellulose (IMDM) buffer with 12.5mM $MgSO_4$ by carefully removing the solution in both reservoirs of the channel and then slowly pipetting 160µL 0.6% methylcellulose (IMDM) with 12.5mM $MgSO_4$ into one reservoir of the channel and aspirating from the other reservoir at the same time without aspirating the solution inside the channel. Both reservoirs were then filled with 60µL 0.6% methylcellulose (IMDM) with 12.5mM $MgSO_4$ buffer.

(4) Imaging the nanotubes on the cell surface:

The cells were then imaged using a spinning disk confocal microscope as described in the step 7 of Supplementary Note S5. At multiple random locations, a stack of images was captured of the cells spanning from the bottoms to the tops of the cells with a stack height of 0.27 µm.

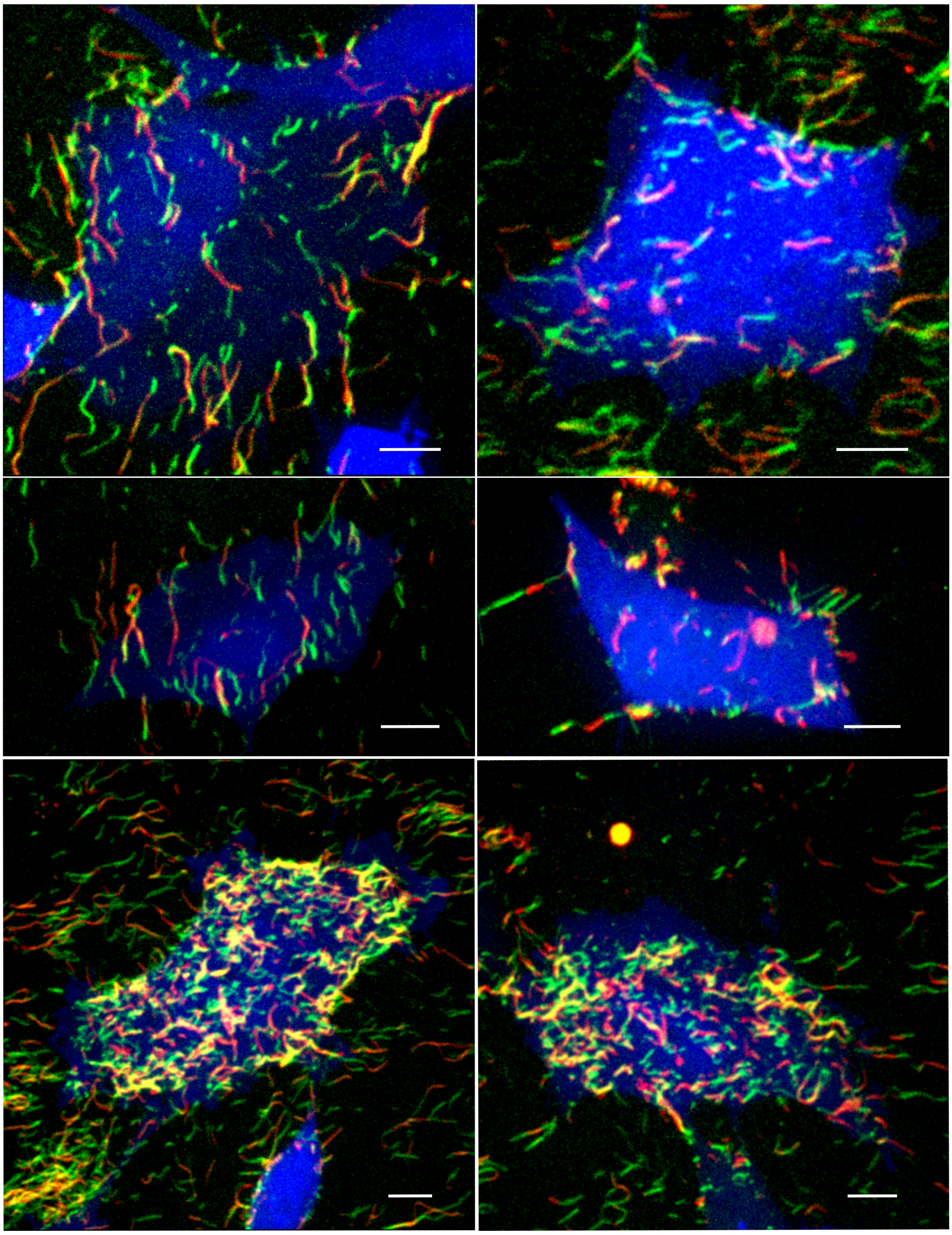

Supplementary Figure S33: 3-dimensional reconstructed images of HeLa cells with anchored nanotubes extended via end-to-end joining in 0.6% methylcellulose. Cells were prepared as described and a stack of micrographs captured as described in Supplementary Note S48. 3-d reconstructed images of cells produced from the captured image stacks are shown. The monomers of both anchored and added nanotubes are labeled with Cy3 (green). The seeds of the anchored, seeded nanotubes and the additional monomers were labeled with atto647 (red). The HeLa cells expressed GFP, which is shown in blue. Scale bars 10µm. This experiment was repeated more than three times independently with similar results.

Section 6. DNA seeded nanotube structure and sequence

Supplementary Note S49: Nanotube monomer structures and sequences

49.1 Nanotube monomers with 6 nucleotide sticky end overhangs (6nt monomers) with and without polyethylene glycol (PEG) modification

The structure of the monomers for the DAE-E nanotubes[21] used in this study was chosen to consider that the temperature for cell culture is 37 ºC. To ensure stability of the assembled nanotubes, the length of the sticky end overhangs was set at 6 nucleotides (Supplementary Figure S34a). These 6nt nanotubes consist of SEs tiles (Supplementary Figure S34a) and assembled when the monomers were incubated with nanotube seeds at 37ºC. To visualize these nanotubes under a fluorescence microscope, each SEs tile monomer was labeled with Cy3 by covalently attaching a Cy3 fluorophore at the 5'of the central strand SEs_3 (Supplementary Figure S34a).

To reduce nonspecific interactions between seeded nanotubes the membranes of live cells, nanotube seeds and nanotubes were each modified with polyethylene glycol (PEG). To coat DNA nanotubes with PEG, the central strand of the 6 nt monomer (SEs_3-5'Cy3) was conjugated with PEG *via* an amine modification (Supplementary Figure S34b, protocol in Supplementary Note S2).

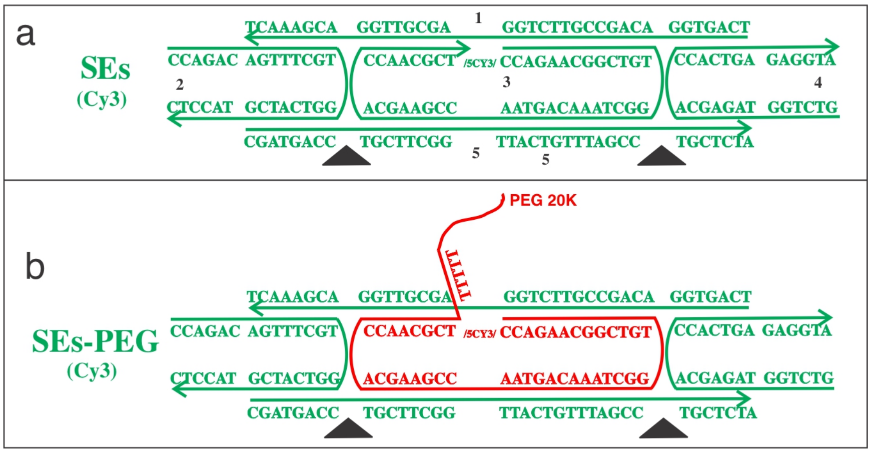

Supplementary Figure S34: The architecture of the Cy3 labeled 6nt nanotube monomer (the SEs tile) with no PEG (a) and with PEG modification (b). **a**. 6nt nanotube monomer without a PEG modification **b.** 6nt nanotube monomer with a PEG modification. The 6nt SEs monomers were labeled with Cy3 by on the central strand SEs_3. Black triangles indicate the locations of crossover points.

During experiments performed to measure the rate of end-to-end joining of 6nt nanotubes in solution, 6nt nanotubes with an atto647 rather than a Cy3 fluorophore were used. The atto647 fluorophore was covalently attached at the 5’of the central strand SEs_3. The structure of the atto647 labeled 6nt nanotube monomer is shown below:

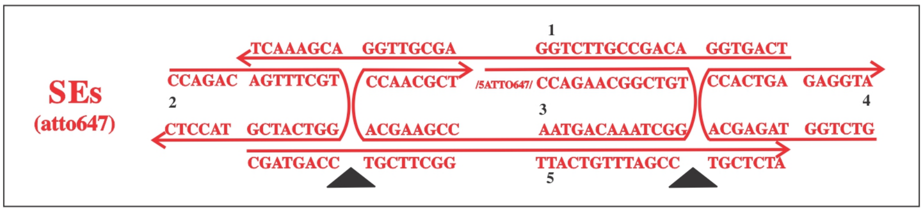

Supplementary Figure S35: The architecture of the atto647 labeled 6nt nanotube monomer. These SEs monomers were labeled with atto647 by labeling the central strands SEs_3. Black triangles indicate the locations of crossover points. These monomers replaced the central strand of the 6nt monomer with a strand whose sequence is the same as the original central strand but is labeled with atto647.

Supplementary Table S27 : Sequences of 6nt SEs nanotube monomer for SEs tiles. For the Cy3 labeled 6nt SEs monemer without PEG coating, the strands of SEs_1, SEs_2, SEs_3-5’Cy3, SEs_4, SEs_5 were mixed together with same concentration excepted that SEs_2 and SEs_4 with doubled concentration. For the Cy3 labeled 6nt SEs monemers with PEG coating, the mixture was the same as that of Cy3 labled 6nt SEs monemers without PEG coating except that the SEs_3-5’Cy3 was replaced by the conjugation of SEs_3-5’Cy3-3’amino and PEG. For the atto647 labeled 6nt SEs monemer, the mixture was the same as that of Cy3 labled 6nt SEs monemer without PEG coating except that the SEs_3-5’Cy3 was replaced by the strand SEs_3-5’ATTO 647.

Strand name	Sequence
**Cy3 labeled 6nt SEs nanotube monomer sequences for SEs tiles withinout PEG modification**	
SEs_1	TCAGTGGACAGCCGTTCTGGAGCGTTGGACGAAACT
SEs_2	CCAGACAGTTTCGTGGTCATCGTACCTC
SEs_3-5’Cy3	/Cy3/CCAGAACGGCTGTGGCTAAACAGTAACCGAAGCACCAACGCT
SEs_4	GTCTGGTAGAGCACCACTGAGAGGTA
SEs_5	CGATGACCTGCTTCGGTTACTGTTTAGCCTGCTCTA
**The central strand of the 6nt SEs monomers used to conjugated with PEG**	
SEs_3-5’Cy3-3’amino	/5Cy3/CCAGAACGGCTGTGGCTAAACAGTAACCGAAGCACCAACGCTTTTTT/3AmMO/
**The central strand of the 6nt SEs monomers labeled with atto647**	
SEs_3-5’ATTO 647	/5ATTO647N/CCAGAACGGCTGTGGCTAAACAGTAACCGAAGCACCAACGCT

/Cy3/ denotes Cy3 fluorophore covalently attached to the 5’ end of DNA.

/3AmMO/ denotes an amino group covalently attached to the 3’ end of DNA.

/5ATTO647N/ denotes atto647 fluorophore covalently attached to the 5’ end of DNA.

49.2 4nt monomers with and without PEG modification

The 4nt nanotube monomer included both SEd tile and REd tile shown in Supplementary Figure S36a. To coat the 4nt nanotube with PEG, both tiles were modified with PEG by conjugating PEG at the central strands SEd_3-5'Cy3 and REd_3-5'Cy3 as shown in Supplementary Figure S36b.

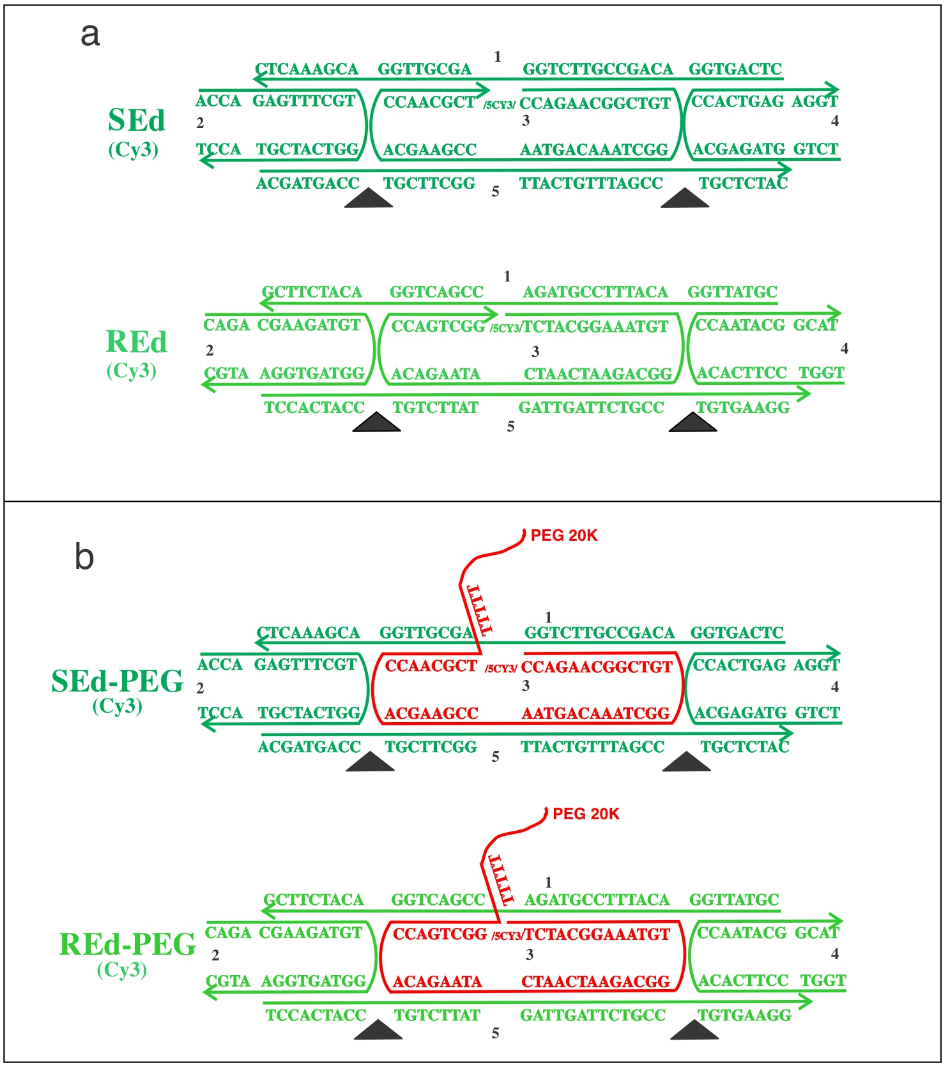

Supplementary Figure S36: The architectures of Cy3 labeled 4nt monomers: SEd and REd tile with and without PEG. **a**. SEd and REd tile without PEG modifications **b.** SEd and REd tile with PEG modifications. The 4nt monomers were labeled with Cy3 by labeling both the central strands SEd_3 and REd_3. Black triangles indicate the locations of crossover points.

The 4nt monomers were labeled with atto488 or atto647 by labeling both the central strands SEd_3 and REd_3 as shown below.

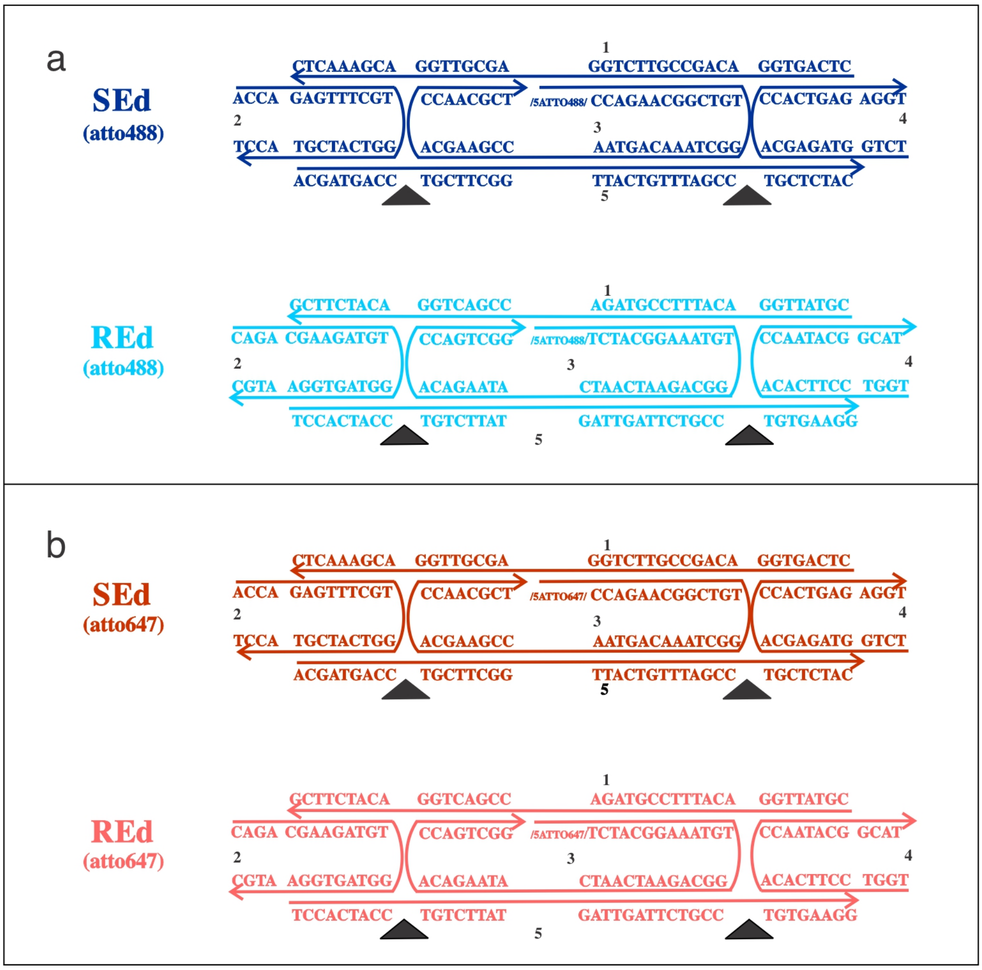

Supplementary Figure S37: Schematics showing the architectures of the 4nt nanotube monomers labeled with atto488 (**a**) and atto647 (**b**) by labeling the central strands (REd_3 and SEd_3) with atto488 or atto647. Black triangles indicate the locations of crossover points.

Supplementary Table S28 : Sequences for the 4nt nanotube monomer for SEd and REd tiles. For the Cy3 labeled 4nt monemer without PEG coating, the strands of SEs_1, SEs_2, SEs_3-5'Cy3, SEs_4, SEs_5, REs_1, REs_2, REs_3-5'Cy3, REs_4, REs_5 were mixed together with same concentration excepted that REs_2, REs_4, REs_2 and REs_4 with doubled concentration. For the Cy3 labeled 4nt monemer with PEG coating, the mixture was the same as that of Cy3 labled 4nt monemer without PEG coating except that the SEs_3-5'Cy3 and REs_3-5'Cy3 were replaced by the conjugations of PEG with SEs_3-5'Cy3-3'amino and REs_3-5'Cy3-3'amino respectivly. For the atto647 labeled 4nt monemer, the mixture was the same as that of Cy3 labled 4nt monemers without PEG coating except that the SEs_3-5'Cy3 and REs_3-5'Cy3 was replaced by the strand SEs_3-5'ATTO 647 and REd_3-5'ATTO 647. For the atto488 labeled 4nt monemer, the mixture was the same as that of Cy3 labled 4nt monemers without PEG coating except that the SEs_3-5'Cy3 and REs_3-5'Cy3 was replaced by the strand SEs_3-5'ATTO 488 and REd_3-5'ATTO 488.

Strand name	Sequence
**4nt Cy3 labeled nanotube monomer sequences without PEG modificaiton:**	
REd_1:	CGTATTGGACATTTCCGTAGACCGACTGGACATCTTCG
REd_2:	CAGACGAAGATGTGGTAGTGGAATGC
REd_3-5'Cy3	/5Cy3/TCTACGGAAATGTGGCAGAATCAATCATAAGACACCAGTCGG
REd_4:	TGGTCCTTCACACCAATACGGCA T
REd_5:	TCCACTACCTGTCTTATGATTGATTCTGCCTGTGAAGG
SEd_1:	CTCAGTGGACAGCCGTTCTGGAGCGTTGGACGAAACTC
SEd_2:	ACCAGAGTTTCGTGGTCATCGTACCT
SEd_3-5'Cy3:	/5Cy3/CCAGAACGGCTGTGGCTAAACAGTAACCGAAGCACCAACGCT
SEd_4:	TCTGGTAGAGCACCACTGAGAGGT
SEd_5:	ACGATGACCTGCTTCGGTTACTGTTTAGCCTGCTCTAC
**The central strand of the 4nt monomers used to conjugated with PEG**	
REd_3-5'Cy3-3'amine	/5Cy3/TCTACGGAAATGTGGCAGAATCAATCATAAGACACCAGTCGGTTTTT/3AmMO/,
SEd_3-5'Cy3-3'amine	/5Cy3/CCAGAACGGCTGTGGCTAAACAGTAACCGAAGCACCAACGCTTTTTT/3AmMO/
**The central strands of the 4nt monomers labeled with atto488:**	
REd_3-5'ATTO488	/5ATTO488N/TCTACGGAAATGTGGCAGAATCAATCATAAGACACCAGTCGG
SEd_3-5'ATTO488	/5ATTO488N/CCAGAACGGCTGTGGCTAAACAGTAACCGAAGCACCAACGCT
**The central strands of the 4nt monomers labeled with atto647:**	
REd_3-5'ATTO647	/5ATTO647N/TCTACGGAAATGTGGCAGAATCAATCATAAGACACCAGTCGG
SEd_3-5'ATTO 647	/5ATTO647N/CCAGAACGGCTGTGGCTAAACAGTAACCGAAGCACCAACGCT

SEd_3-5'Cy3-3'amine has the same sequence as SEs_3-5'Cy3-3'amine (Supplementary Note 1.1).

SEd_3-5'ATTO 647: has the same sequence as SEs_3-5'ATTO 647 (Supplementary Note 49.1).

/5ATTO488N/ denotes atto488 fluorophore covalently attached to the 5' end of DNA.

49.3 4nt inactive monomer design

To make it possible to add monomers that had not already assembled during experiments on nanotube joining, we used 4nt REd monomers (REd5) where one of the sticky ends was blocked. An activation strand was then added to the solution the inactive REd tiles were added to form active monomers. Because these monomers did not assemble spontaneously, it was possible to anneal these inactive monomers at very high concentrations without any unseeded nanotube growth.

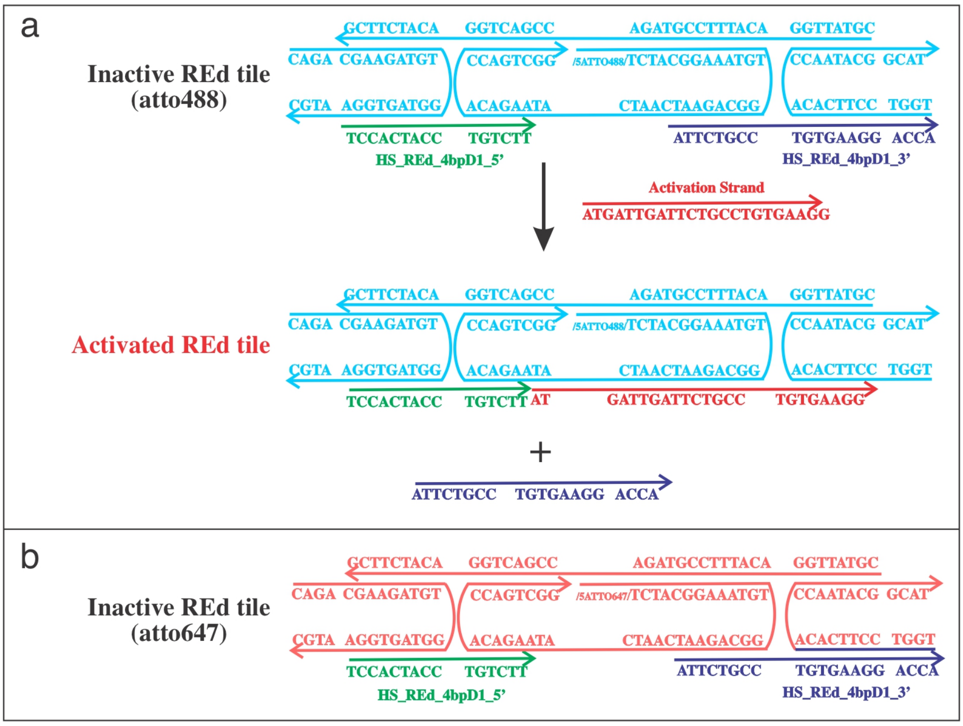

Supplementary Figure S38: The 4nt inactive REd monomer was activated by adding the "activation strand", which replaced the strand HS_REd_4ntD1_3' by strand displacement. Inactive REd monomers labelled with atto488 (**a**) and atto647 (**b**).

4nt Atto488-labelled nanotubes were formed from atto488-labeled inactive REd monomers (Supplementary Figure S38a), atto488-labeled 4nt SEd tile (Supplementary Figure S38a) and an *activation strand* (Supplementary Figure S38a). 4nt atto647-labelled nanotubes were formed from atto647 labeled inactive REd monomers (Supplementary Figure S38b), and atto647-labeled 4nt SEd tile (Supplementary Figure S33b) and an *activation strand* (Supplementary Figure S38a).

Supplementary Table S29 : Sequences for inactive 4nt REd monomer and the activation strand.

Strand name	Sequence
**Inactive tile strands**	
HS_REd_4bpD1_5'	TCCACTACCTGTCTT
HS_REd_4bpD1_3'	ATTCTGCCTGTGAAGGACCA
**Activation strand**	
Activation strand	ATGATTGATTCTGCCTGTGAAGG

Supplementary Note S50: Design and sequences of DNA nanotube seeds

DNA origami seeds both serve as nuclei and direct the assembly of DNA nanotubes and function as anchors for DNA nanotubes to attach them to specific receptors on cell membranes. The different parts of the DNA origami seeds were designed to realize these functions (Supplementary Figure S39). The detailed design of each part is shown below.

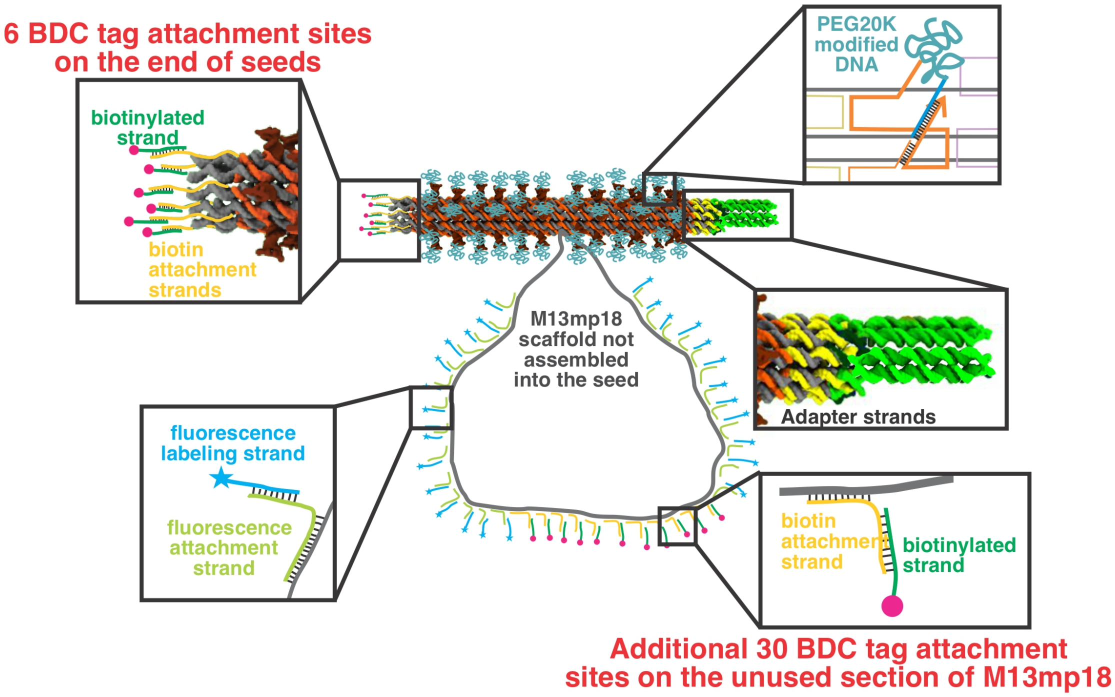

Supplementary Figure S39: Schematic for DNA nanotube seeds showing the positions of adapters, PEG modification sites, fluorescent labeling sites, and biotin attachment sites.

50.1 DNA nanotube seed staples for PEG-coated seeds:

The DNA nanotube seeds used in this work were adopted from Mohammed *et al.*[8] In the nanotube seed used in Mohammed *et al.*[8], the staples presented DNA hairpins at even helical turns so that all the hairpins were on one side of the unrolled seed.[8] We modified each of these staples by breaking the hairpin into two parts and extending one of the resulting sequences by 15 nucleotides (Supplementary Figures S40 and S41). These extended single stranded DNA tags could be used as binding sites for conjugates of DNA with other molecules. Here they were used as binding sites for PEG-DNA conjugate to coat the seeds with PEG.

To reduce the nonspecific attachment of seeds to the cell membrane, here we coated the nanotube seeds with PEG by using PEG-DNA conjugates. The sequence of the PEG-DNA conjugate was complementary with the 15nucleotide sequence extending from each staple strand. The unmodified seeds with binding sites were first be assembled and purified. Then the PEG-DNA conjugate was added to these unmodified seeds solution to bind to all the 72 binding sites extended from the seeds by DNA hybridization to coat the whole nanotube seeds with PEG (Supplementary Figures S40 and S41).

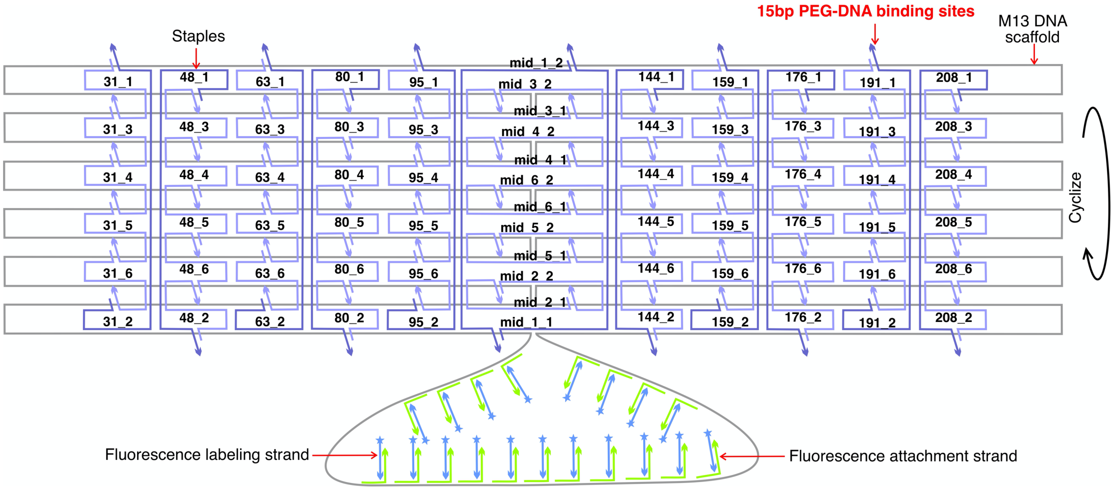

Supplementary Figure S40. Map showing the positions of the staple strands, PEG-DNA conjugate binding sites, and fluorescence labeling strand sites and fluorescence labeling strands on the seed.

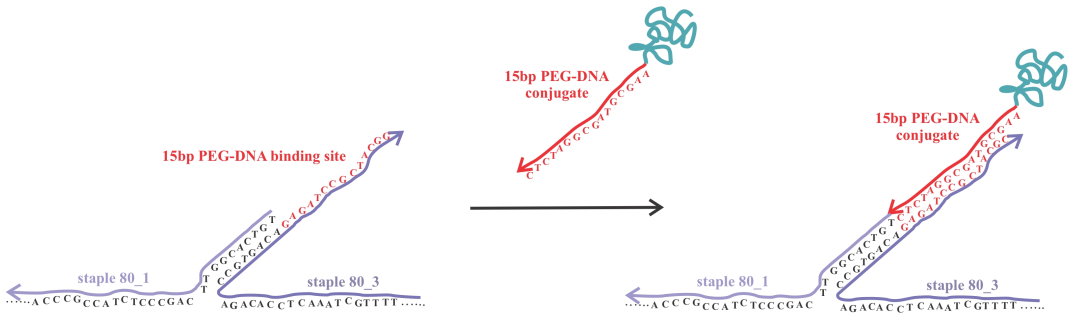

Supplementary Figure S41: Structure of the 15 nucleotide DNA overhang on a staple that serves as an attachment site for a PEG-DNA conjugate.

Supplementary Table S30 : Stable sequences of the DNA nanotube seeds with 15 nucleotide DNA overhang for PEG-DNA attachment and sequence for the amino-modified DNA strand used to produce the PEG-DNA conjugate that binds to seeds.

Strand name	Sequence
**Staple sequences for the DNA nanotube seed**	
31_1	CAGCTCACTTTCATTTTCCGTAACACTGAGTTTCAAAGGAACGTCCGTCCGAGATCCGACTACGC
31_2	GTCAACGCTTAGATTTAGCGCCAAAAGGAATTACCACCACCCGTGAGCTGGAGATCCGACTACGC
31_3	GGACGGACTTAACTAAAGATCTCCAAAAAAAAGGCTTTTGCGGTGGACACGAGATCCGACTACGC
31_4	GTGTCCACTTGGATCGTCGGGTAGCAACGGCTACAAGTACAAGGTGCCACGAGATCCGACTACGC
31_5	GTGGCACCTTCGGAGATTCGCGACCTGCTCCATGACGTAACAGGCAATGCGAGATCCGACTACGC
31_6	GCATTGCCTTAAGCTGCTACACCAGAACGAGTAGATCAGTTGGCGTTGACGAGATCCGACTACGC
48_1	GCAACTCGTTTACAAACTCCGCCACCCTCAGAGCGAGGCATACCGTCCACGAGATCCGACTACGC
48_2	GTGGACGGTTGTAAGAGCACAGGTAGAAAGATTCTAAATTGGGCGAACACGAGATCCGACTACGC
48_3	GCTGCACCTTGGAGCCTTAGCGGAGTGAGAATAGGTCACCAGCGAGTTGCGAGATCCGACTACGC
48_4	GGCTCGACTTTGAGGACTAGGGAGTTAAAGGCCGCTCCAAAAGGTGCAGCGAGATCCGACTACGC
48_5	GTGTCAGCTTCCGGAACGTACCAAGCGCGAAACAAGAGGCTTGTCGAGCCGAGATCCGACTACGC
48_6	GTG TTCGCTTGCTTGAGATTCATTACCCAAATCATTACTTAGGCTGACACGAGATCCGACTACGC
63_1	CAGCCGAGTTCCTCAGAAACAACGCCTGTAGCATAACTTTCACCACACCTGAGATCCGACTACGC
63_2	AGGACTGCTTACATTATTAACACTATCATAACCCACCGCCACCTCGGCTGGAGATCCGACTACGC
63_3	AGGTGTGGTTACAGTTTCTAATTGTATCGGTTTAGGTCGCTGGCGACATGGAGATCCGACTACGC
63_4	CATGTCGCTTAGGCTTGCAAAGACTTTTTCATGATGACCCCCGAACGATGGAGATCCGACTACGC
63_5	CATCGTTCTTAGCGATTAAGGCGCAGACGGTCAATGACAAGAGCCTCACAGAGATCCGACTACGC
63_6	TGTGAGGCTTACCGGATATGGTTTAATTTCAACTACGGAACAGCAGTCCTGAGATCCGACTACGC
80_1	TGTCACGGTTCAGCCCTCTACCGCCACCCTCAGATCGTTTACACCGCATCGAGATCCGACTACGC
80_2	GATGCGGTTTCAGACGACTTAATAAAACGAACTATTAATCATGCCAGACAGAGATCCGACTACGC
80_3	CAGCACAGTTCTTTCGAGTGGGATTTTGCTAAACTCCACAGACCGTGACAGAGATCCGACTACGC
80_4	ACACCGAGTTCCATTAAACATAACCGATATATTCTCAGCTTGCTGTGCTGGAGATCCGACTACGC
80_5	GGACAAGGTTGAACCGAACTAAAACACTCATCTTGGAAGTTTCTCGGTGTGAGATCCGACTACGC
80_6	TGTCTGGCTTTGTGAATTTCATCAAGAGTAATCTTCATAAGGCCTTGTCCGAGATCCGACTACGC
95_1	GCACCGCTTTAGGTTTAGATAGTTAGCGTAACGAAAATGAATCTGCCAACGAGATCCGACTACGC
95_2	GTCATGGCTTAATCTACGGATAAAAACCAAAATATACTCAGGAGCGGTGCGAGATCCGACTACGC
95_3	GTTGGCAGTTTTTCTGTAGTGAATTTCTTAAACAACAACCATGCTCAGACGAGATCCGACTACGC
95_4	GTCTGAGCTTCGCCCACGCGGGTAAAATACGTAAGAGGCAAAGTGCTGTCGAGATCCGACTACGC
95_5	GACAGCACTTAGAATACACTGACCAACTTTGAAAATAGGCTGCTGCATCCGAGATCCGACTACGC
95_6	GGATGCAGTTGCTGACCTACCTTATGCGATTTTAGGAAGAAAGCCATGACGAGATCCGACTACGC
mid_1_1	GGCAGACGTTCTTTTGCAATCCTGAATCTTACCATATAAGTACGGTCAGCGAGATCCGACTACGC
mid _1_2	GCTGACCGTTTAGCCCGGAATAGGTGTATCACCGGCGAGAGGCGTCTGCCGAGATCCGACTACGC
mid _2_1	CAGGTGGATTGTAATTGAACCAGTCAGGACGTTGAGAACTGGCGTCGGTCGAGATCCGACTACGC
mid _2_2	GACCGACGTTCTCATTATGCGCTAATATCAGAGAGTCAGAGGTCCACCTGGAGATCCGACTACGC
mid _3_1	GGATGCCATTCTTTTGATCTTTCCAGACGTTAGTTCTAAAGTCGTCCAGCGAGATCCGACTACGC
mid _3_2	GCTGGACGTTTTTGTCGTGATACAGGAGTGTACTATACATGGTGGCATCCGAGATCCGACTACGC
mid _4_1	CAGGACACTTGGAACCGCTGCGCCGACAATGACAGCTTGATACGGAGTACGAGATCCGACTACGC

mid _4_2	GTACTCCGTTCCGATAGTCTCCCTCAGAGCCGCCCCACCACCGTGTCCTGGAGATCCGACTACGC
mid _5_1	GCCTGCGGTTTAGCAAACTGTACAGACCAGGCGCGAGGACAGAGTGCAACGAGATCCGACTACGC
mid _5_2	GTTGCACTTTATGAACGGGTAGAAAATACATACACAGTATGTCCGCAGGCGAGATCCGACTACGC
mid _6_1	GCCTGACGTTGAATTAGACCAACCTAAAACGAAATGCCACTACCGTAAGCGAGATCCGACTACGC
mid _6_2	GCTTACGGTTCGAAGGCAGCCAGCAAAATCACCACCATTTGGCGTCAGGCGAGATCCGACTACGC
144_1	AGGAGCACTTGTTTTAACCCGTCGAGAGGGTTGAACGCTAACAGCGAACCGAGATCCGACTACGC
144_2	GGTTCGCTTTGAGCGTCTGAACACCCTGAACAAAGATAACCCGTTTGGACGAGATCCGACTACGC
144_3	CAGCCGTGTTAACCGCCACGTTCCAGTAAGCGTCGGTAATAAGTGCTCCTGAGATCCGACTACGC
144_4	GTGATGGCTTATTACCATATCACCGGAACCAGAGACCCTCAGCACGGCTGGAGATCCGACTACGC
144_5	TGGAGTGCTTGCAACATACCGTCACCGACTTGAGGTAGCACCGCCATCACGAGATCCGACTACGC
144_6	GTCCAAACTTACAAGAATAAGACTCCTTATTACGTAAAGGTGGCACTCCAGAGATCCGACTACGC
159_1	GATGGACTTTGATAAGTGGGGGTCAGTGCCTTGACAGTCTCTAGCGTCCTGAGATCCGACTACGC
159_2	GGCAACCGTTAATTAACTTTCCAGAGCCTAATTTACCAGGCGAGTCCATCGAGATCCGACTACGC
159_3	AGGACGCTTTGAATTTACCCCTCAGAGCCACCACTCTTTTCACGGAGGCAGAGATCCGACTACGC
159_4	TGCCTCCGTTTAATCAAATAGCAAGGCCGGAAACTAAAGGTGCGAGCACAGAGATCCGACTACGC
159_5	TGTGCTCGTTAATTATCATAAAAGAAACGCAAAGAAGAACTGCTGGTGCCGAGATCCGACTACGC
159_6	GGCACCAGTTGCATGATTTGAGTTAAGCCCAATAGACGGGAGCGGTTGCCGAGATCCGACTACGC
176_1	GATGGTCCTTGCCCGTATCGGGGTTTTGCTCAGTGCCAGTTAGCAGGAGCGAGATCCGACTACGC
176_2	GCTCCTGCTTCAAAATAAACAGGGAAGCGCATTAATAAGAGCCGGTCGCAGAGATCCGACTACGC
176_3	GAACAGCGTTCCGCCACCGCCAGAATGGAAAGCGGTAACAGTGGACCATCGAGATCCGACTACGC
176_4	GCTAGGCATTTGAAACCATTATTAGCGTTTGCCACCTCAGAGCGCTGTTCGAGATCCGACTACGC
176_5	GTGAGCCATTGAATAAGTGACGGAAATTATTCATGTCACCAATGCCTAGCGAGATCCGACTACGC
176_6	TGCGACCGTTAAGAAACAATAACGGAATACCCAAACACCACGTGGCTCACGAGATCCGACTACGC
191_1	GCAAGCGGTTAGGATTAGAAACAGTTAATGCCCCATAAATCCTGCCTTACGAGATCCGACTACGC
191_2	GAAGGTCGTTACATAAAAACAGCCATATTATTTAGAAGGATTCCGCTTGCGAGATCCGACTACGC
191_3	GTAAGGCATTTCATTAAAAGAACCACCACCAGAGTTCGGTCACGCTCATCGAGATCCGACTACGC
191_4	GATGAGCGTTTAGCCCCCTCGATAGCAGCACCGTAGGGAAGGCGACACCAGAGATCCGACTACGC
191_5	TGGTGTCGTTTAAATATTTTATTTTGTCACAATCCCGAGGAACCAGTGCGGAGATCCGACTACGC
191_6	CGCACTGGTTACGCAATAATGAAATAGCAATAGCAGAGAATACGACCTTCGAGATCCGACTACGC
208_1	GGACCGTGTTTTCGGAACCTGAGACTCCTCAAGATCCCAATCCGGACACAGAGATCCGACTACGC
208_2	TGTGTCCGTTCAAATAAGATAGCAGCCTTTACAGTATCTTACCCTGTTGCGAGATCCGACTACGC
208_3	GTGGCTCGTTAGCATTGATGATATTCACAAACAACTGCCTATCACGGTCCGAGATCCGACTACGC
208_4	CATCGCTCTTGCGACAGACGTTTTCATCGGCATTCCGCCGCCCGAGCCACGAGATCCGACTACGC
208_5	CAGGTGACTTATTCATATTTCAACCGATTGAGGGAATCAGTAGAGCGATGGAGATCCGACTACGC
208_6	GCAACAGGTTCGAAGCCCAGTTACCAGAAGGAAAAATAGAAAGTCACCTGGAGATCCGACTACGC

Sequence for the amino-modified DNA strand used to produce a PEG-DNA conjugate that binds to seeds

Seed PEG-attachment strand	/5AmMC6T/AAGCGTAGTCGGATCTC

50.2 Strands for fluorescently labeling DNA nanotube seeds:

To visualize the seeds using fluorescence and confocal microscopy, the seeds were labelled with fluorophores attached to fluorescent labeling strands that can bind to specific regions of the scaffold. This labeling strategy was adopted from Mohammed *et al.*[8], in which 100 fluorescent labeling attachment strands were designed to bind to regions of the M13mp18 scaffold that are not used to fold the seed. Each of these attachment strands includes two domains. One domain of an attachment strand binds to the section of the M13mp18 scaffold that is not folded by the staples for the seed, while the other domain of the attachment strand binds to a labeling strand with an ATTO fluorophore.

Supplementary Table S31 : Sequences of the fluorescent labeling strands and the fluorescence attachment strands on M13

Strand name	Sequence
**Fluorescent labeling strand sequences**	
Labeling strand ATTO488	/5ATTO488N/TTCTATCCACCTCACCA
Labeling strand ATTO647	/5ATTO647N/AACTATCCACCTCACCA
**Fluorescence attachment strand sequences**	
Unused_m13mp18_01_OLS	AAATTCTTACCAGTATAAAGCCAACTTTTTGGTGAGGTGGATAG
Unused_m13mp18_02_OLS	GCCTGTTTAGTATCATATGCGTTATTTTTTGGTGAGGTGGATAG
Unused_m13mp18_03_OLS	ACACCGGAATCATAATTACTAGAAATTTTTGGTGAGGTGGATAG
Unused_m13mp18_04_OLS	GATAAATAAGGCGTTAAATAAGAATTTTTGGTGAGGTGGATAG
Unused_m13mp18_05_OLS	TTTAATGGTTTGAAATACCGACCGTTTTTGGTGAGGTGGATAG
Unused_m13mp18_06_OLS	TTAGTTAATTTCATCTTCTGACCTATTTTTGGTGAGGTGGATAG
Unused_m13mp18_07_OLS	ACGCGAGAAAACTTTTTCAAATATATTTTTGGTGAGGTGGATAG
Unused_m13mp18_08_OLS	GATGCAAATCCAATCGCAAGACAAATTTTTGGTGAGGTGGATAG
Unused_m13mp18_09_OLS	TGGGTTATATAACTATATGTAAATGTTTTTGGTGAGGTGGATAG
Unused_m13mp18_10_OLS	ACTACCTTTTTAACCTCCGGCTTAGTTTTTGGTGAGGTGGATAG
Unused_m13mp18_11_OLS	AATTTATCAAAATCATAGGTCTGAGTTTTTGGTGAGGTGGATAG
Unused_m13mp18_12_OLS	TTAAGACGCTGAGAAGAGTCAATAGTTTTTGGTGAGGTGGATAG
Unused_m13mp18_13_OLS	TCCTTGAAAACATAGCGATAGCTTATTTTTGGTGAGGTGGATAG
Unused_m13mp18_14_OLS	TCGCTATTAATTAATTTTCCCTTAGTTTTTGGTGAGGTGGATAG
Unused_m13mp18_15_OLS	AGTGAATAACCTTGCTTCTGTAAATTTTTTGGTGAGGTGGATAG
Unused_m13mp18_16_OLS	GAAACAGTACATAAATCAATATATGTTTTTGGTGAGGTGGATAG
Unused_m13mp18_17_OLS	ATTTCATTTGAATTACCTTTTTTAATTTTTGGTGAGGTGGATAG
Unused_m13mp18_18_OLS	AGAAAACAAAATTAATTACATTTAATTTTTGGTGAGGTGGATAG
Unused_m13mp18_19_OLS	CAAAAGAAGATGATGAAACAAACATTTTTTGGTGAGGTGGATAG
Unused_m13mp18_20_OLS	GCGAATTATTCATTTCAATTACCTGTTTTTGGTGAGGTGGATAG
Unused_m13mp18_21_OLS	AATACCAAGTTACAAAATCGCGCAGTTTTTGGTGAGGTGGATAG
Unused_m13mp18_22_OLS	CAATAACGGATTCGCCTGATTGCTTTTTTTGGTGAGGTGGATAG
Unused_m13mp18_23_OLS	TAACAGTACCTTTTACATCGGGAGATTTTTGGTGAGGTGGATAG
Unused_m13mp18_24_OLS	CAGGTTTAACGTCAGATGAATATACTTTTTGGTGAGGTGGATAG
Unused_m13mp18_25_OLS	CAGAAATAAAGAAATTGCGTAGATTTTTTTGGTGAGGTGGATAG

Unused_m13mp18_26_OLS	CCATATCAAAATTATTTGCACGTAATTTTTGGTGAGGTGGATAG
Unused_m13mp18_27_OLS	TCTGAATAATGGAAGGGTTAGAACCTTTTTGGTGAGGTGGATAG
Unused_m13mp18_28_OLS	TATAATCCTGATTGTTTGGATTATATTTTTGGTGAGGTGGATAG
Unused_m13mp18_29_OLS	GATTATCAGATGATGGCAATTCATCTTTTTGGTGAGGTGGATAG
Unused_m13mp18_30_OLS	AAGGAGCGGAATTATCATCATATTCTTTTTGGTGAGGTGGATAG
Unused_m13mp18_31_OLS	CATTTTGCGGAACAAAGAAACCACCTTTTTGGTGAGGTGGATAG
Unused_m13mp18_32_OLS	TAATTTTAAAAGTTTGAGTAACATTTTTTTGGTGAGGTGGATAG
Unused_m13mp18_33_OLS	GTATTAAATCCTTTGCCCGAACGTTTTTTTGGTGAGGTGGATAG
Unused_m13mp18_34_OLS	TAGACTTTACAAACAATTCGACAACTTTTTGGTGAGGTGGATAG
Unused_m13mp18_35_OLS	ATAATACATTTGAGGATTTAGAAGTTTTTTGGTGAGGTGGATAG
Unused_m13mp18_36_OLS	CAACTAATAGATTAGAGCCGTCAATTTTTTGGTGAGGTGGATAG
Unused_m13mp18_37_OLS	TATCTAAAATATCTTTAGGAGCACTTTTTTGGTGAGGTGGATAG
Unused_m13mp18_38_OLS	ACTGATAGCCCTAAAACATCGCCATTTTTTGGTGAGGTGGATAG
Unused_m13mp18_39_OLS	GAATGGCTATTAGTCTTTAATGCGCTTTTTGGTGAGGTGGATAG
Unused_m13mp18_40_OLS	AGAATACGTGGCACAGACAATATTTTTTTTGGTGAGGTGGATAG
Unused_m13mp18_41_OLS	ATAGAACCCTTCTGACCTGAAAGCGTTTTTGGTGAGGTGGATAG
Unused_m13mp18_42_OLS	ATAAAAGGGACATTCTGGCCAACAGTTTTTGGTGAGGTGGATAG
Unused_m13mp18_43_OLS	GCAGATTCACCAGTCACACGACCAGTTTTTGGTGAGGTGGATAG
Unused_m13mp18_44_OLS	ATCGTCTGAAATGGATTATTTACATTTTTTGGTGAGGTGGATAG
Unused_m13mp18_45_OLS	ATGGAAATACCTACATTTTGACGCTTTTTTGGTGAGGTGGATAG
Unused_m13mp18_46_OLS	CCAGCCATTGCAACAGGAAAAACGCTTTTTGGTGAGGTGGATAG
Unused_m13mp18_47_OLS	CTGGTAATATCCAGAACAATATTACTTTTTGGTGAGGTGGATAG
Unused_m13mp18_48_OLS	GTAGAAGAACTCAAACTATCGGCCTTTTTTGGTGAGGTGGATAG
Unused_m13mp18_49_OLS	TGATTAGTAATAACATCACTTGCCTTTTTTGGTGAGGTGGATAG
Unused_m13mp18_50_OLS	AAATTAACCGTTGTAGCAATACTTCTTTTTGGTGAGGTGGATAG
Unused_m13mp18_51_OLS	CCGAGTAAAAGAGTCTGTCCATCACTTTTTGGTGAGGTGGATAG
Unused_m13mp18_52_OLS	GAAGTGTTTTTATAATCAGTGAGGCTTTTTGGTGAGGTGGATAG
Unused_m13mp18_53_OLS	GACAGGAACGGTACGCCAGAATCCTTTTTTGGTGAGGTGGATAG
Unused_m13mp18_54_OLS	AACAGGAGGCCGATTAAAGGGATTTTTTTTGGTGAGGTGGATAG
Unused_m13mp18_55_OLS	TCCTCGTTAGAATCAGAGCGGGAGCTTTTTGGTGAGGTGGATAG
Unused_m13mp18_56_OLS	GCTTTGACGAGCACGTATAACGTGCTTTTTGGTGAGGTGGATAG
Unused_m13mp18_57_OLS	CGCCGCTACAGGGCGCGTACTATGGTTTTTGGTGAGGTGGATAG
Unused_m13mp18_58_OLS	TAACCACCACACCCGCCGCGCTTAATTTTTGGTGAGGTGGATAG
Unused_m13mp18_59_OLS	TGGCAAGTGTAGCGGTCACGCTGCGTTTTTGGTGAGGTGGATAG
Unused_m13mp18_60_OLS	AAGCGAAAGGAGCGGGCGCTAGGGCTTTTTGGTGAGGTGGATAG
Unused_m13mp18_61_OLS	CGAACGTGGCGAGAAAGGAAGGGAATTTTTGGTGAGGTGGATAG
Unused_m13mp18_62_OLS	GATTTAGAGCTTGACGGGGAAAGCCTTTTTGGTGAGGTGGATAG
Unused_m13mp18_63_OLS	TAAATCGGAACCCTAAAGGGAGCCCTTTTTGGTGAGGTGGATAG
Unused_m13mp18_64_OLS	TTTTGGGGTCGAGGTGCCGTAAAGCTTTTTGGTGAGGTGGATAG
Unused_m13mp18_65_OLS	TACGTGAACCATCACCCAAATCAAGTTTTTGGTGAGGTGGATAG
Unused_m13mp18_66_OLS	AAACCGTCTATCAGGGCGATGGCCCTTTTTGGTGAGGTGGATAG
Unused_m13mp18_67_OLS	ACGTGGACTCCAACGTCAAAGGGCGTTTTTGGTGAGGTGGATAG
Unused_m13mp18_68_OLS	TTTGGAACAAGAGTCCACTATTAAATTTTTGGTGAGGTGGATAG

Unused_m13mp18_69_OLS	CCGAGATAGGGTTGAGTGTTGTTCCTTTTTGGTGAGGTGGATAG
Unused_m13mp18_70_OLS	AAATCCCTTATAAATCAAAAGAATATTTTTGGTGAGGTGGATAG
Unused_m13mp18_71_OLS	TGTTTGATGGTGGTTCCGAAATCGGTTTTTGGTGAGGTGGATAG
Unused_m13mp18_72_OLS	CTGGTTTGCCCCAGCAGGCGAAAATTTTTTGGTGAGGTGGATAG
Unused_m13mp18_73_OLS	TGAGAGAGTTGCAGCAAGCGGTCCATTTTTGGTGAGGTGGATAG
Unused_m13mp18_74_OLS	AGCTGATTGCCCTTCACCGCCTGGCTTTTTGGTGAGGTGGATAG
Unused_m13mp18_75_OLS	TTTCTTTTCACCAGTGAGACGGGCATTTTTGGTGAGGTGGATAG
Unused_m13mp18_76_OLS	GTTTGCGTATTGGGCGCCAGGGTGGTTTTTGGTGAGGTGGATAG
Unused_m13mp18_77_OLS	GAATCGGCCAACGCGCGGGGAGAGGTTTTTGGTGAGGTGGATAG
Unused_m13mp18_78_OLS	GAAACCTGTCGTGCCAGCTGCATTATTTTTGGTGAGGTGGATAG
Unused_m13mp18_79_OLS	TGCGCTCACTGCCCGCTTTCCAGTCTTTTTGGTGAGGTGGATAG
Unused_m13mp18_80_OLS	GAGTGAGCTAACTCACATTAATTGCTTTTTGGTGAGGTGGATAG
Unused_m13mp18_81_OLS	TAAAGTGTAAAGCCTGGGGTGCCTATTTTTGGTGAGGTGGATAG
Unused_m13mp18_82_OLS	TTCCACACAACATACGAGCCGGAAGTTTTTGGTGAGGTGGATAG
Unused_m13mp18_83_OLS	CTGTGTGAAATTGTTATCCGCTCACTTTTTGGTGAGGTGGATAG
Unused_m13mp18_84_OLS	ATTCGTAATCATGGTCATAGCTGTTTTTTTGGTGAGGTGGATAG
Unused_m13mp18_85_OLS	TAGAGGATCCCCGGGTACCGAGCTCTTTTTGGTGAGGTGGATAG
Unused_m13mp18_86_OLS	CAAGCTTGCATGCCTGCAGGTCGACTTTTTGGTGAGGTGGATAG
Unused_m13mp18_87_OLS	ACGACGTTGTAAAACGACGGCCAGTTTTTTGGTGAGGTGGATAG
Unused_m13mp18_88_OLS	TTGGGTAACGCCAGGGTTTTCCCAGTTTTTGGTGAGGTGGATAG
Unused_m13mp18_89_OLS	AGGGGGATGTGCTGCAAGGCGATTATTTTTGGTGAGGTGGATAG
Unused_m13mp18_90_OLS	CTCTTCGCTATTACGCCAGCTGGCGTTTTTGGTGAGGTGGATAG
Unused_m13mp18_91_OLS	CTGTTGGGAAGGGCGATCGGTGCGGTTTTTGGTGAGGTGGATAG
Unused_m13mp18_92_OLS	GCGCCATTCGCCATTCAGGCTGCGCTTTTTGGTGAGGTGGATAG
Unused_m13mp18_93_OLS	CGCTTCTGGTGCCGGAAACCAGGCATTTTTGGTGAGGTGGATAG
Unused_m13mp18_94_OLS	ATCGCACTCCAGCCAGCTTTCCGGCTTTTTGGTGAGGTGGATAG
Unused_m13mp18_95_OLS	GACGACGACAGTATCGGCCTCAGGATTTTTGGTGAGGTGGATAG
Unused_m13mp18_96_OLS	GTAACCGTGCATCTGCCAGTTTGAGTTTTTGGTGAGGTGGATAG

50.3 Adapter design and sequences

The design of the adapter sequences is based on the designs for DNA nanotube seed adapters in Mohammed *et al* [8]. The seed 6nt A adapters serve as templates for SEs nanotube growth at the right side of the seeds as illustrated, with corresponding attachment strands, for other molecules or structures on the left side of the seeds as illustrated. The seed 6nt B adapters were designed to serve as templates for the SEs nanotube growth from the left side of the seeds as illustrated, with attachment strands for other molecules or structures on the right side of the seeds as illustrated. While a variety of attachment chemistries were used on the ends of seeds not used to template growth in this work, biotin attachment strands are depicted as examples here.

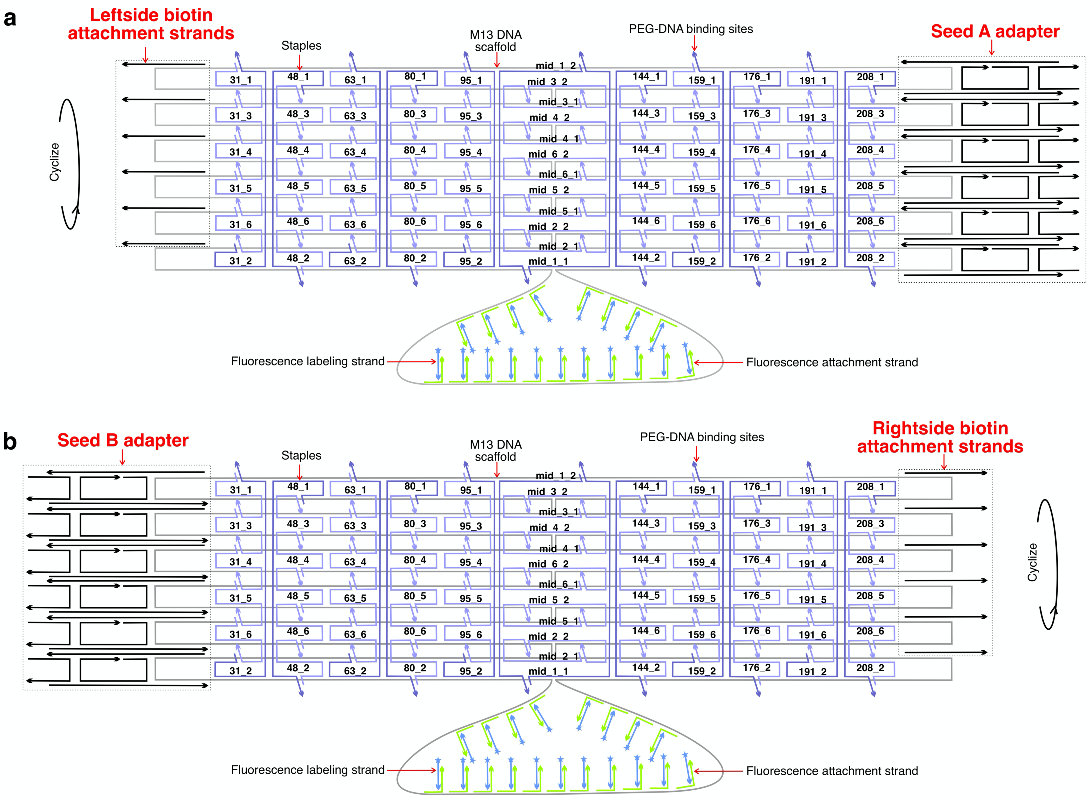

Supplementary Figure S42: Adapter organization on nanotube seeds. A. Map showing the positions of the seed A adapter and the corresponding left-side biotin attachment strands **b.** Map showing the positions of the seed B adapters and the corresponding right-side biotin attachment strands.

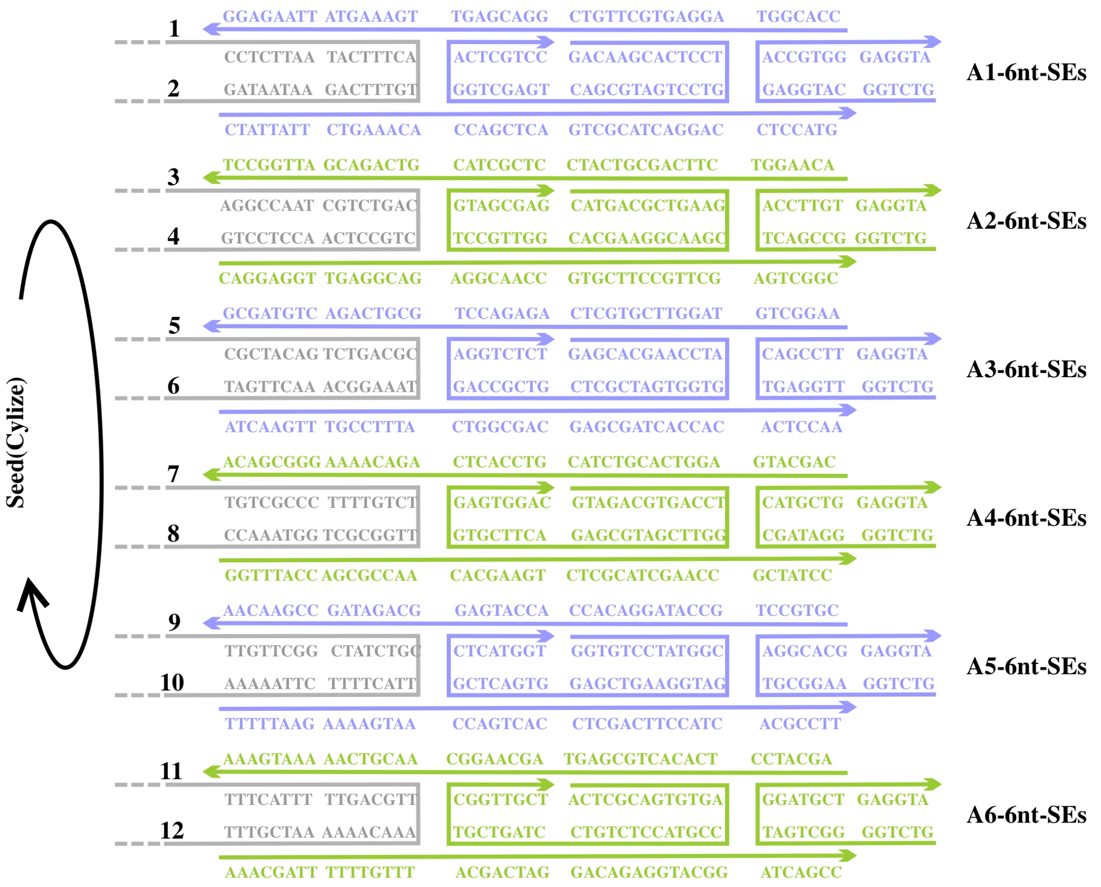

Supplementary Figure S43: Structures of the assembled seed A adapters for 6 nt nanotubes. The gray lines and corresponding sequences are parts of the M13mp18 scaffold.

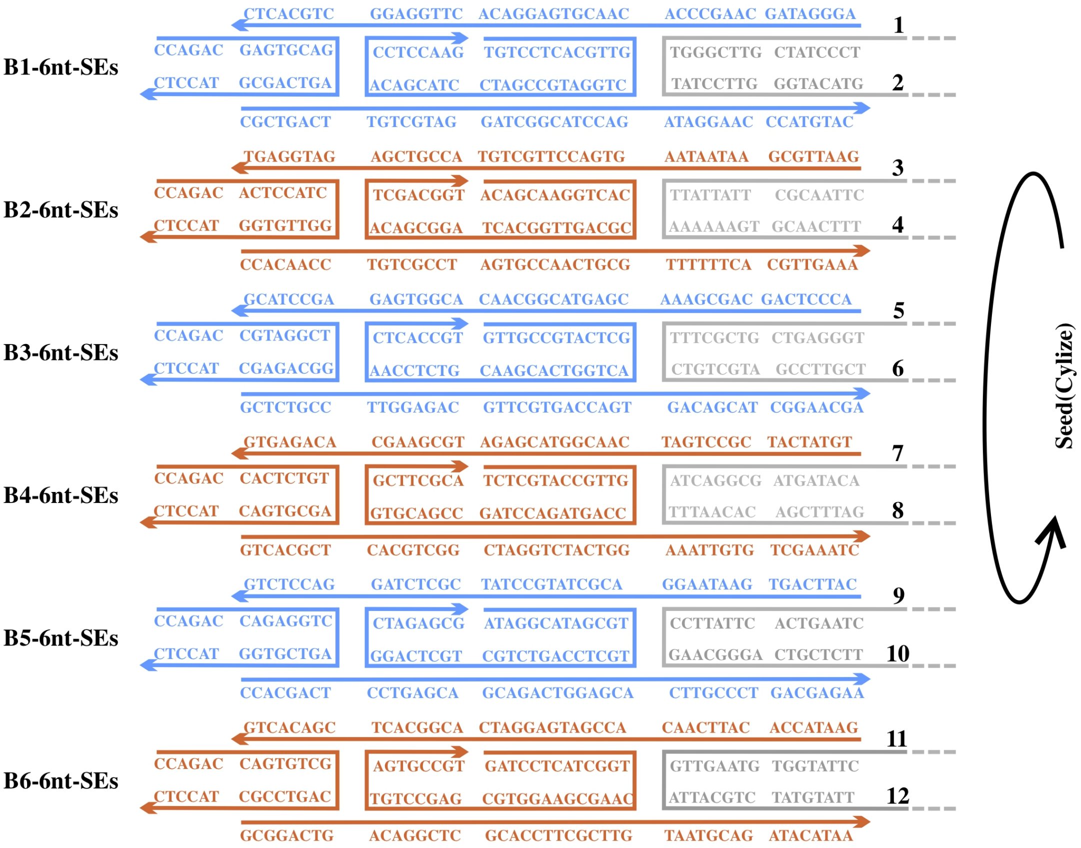

Supplementary Figure S44: Structures of the assembled seed B adapters for 6 nt nanotubes. The gray lines and corresponding sequences are parts of the M13mp18 scaffold.

Supplementary Table S32 : Sequences of 6nt seed A adapters and 6nt seed B adapters.

Strand name	Sequence
**6nt Seed A adapter sequences**	
A1-6nt-SEs_1	CCACGGTAGGAGTGCTTGTCGGACGAGTTGAAAGTATTAAGAGG
A1-6nt-SEs_3	GACAAGCACTCCTGTCCTGATGCGACTGAGCTGGACTCGTCC
A1-6nt-SEs_5	CTATTATTCTGAAACACCAGCTCAGTCGCATCAGGACCTCCATG
A1-6nt-SEs_4	GTCTGGCATGGAGACCGTGGGAGGTA
A2-6nt-SEs_1	ACAAGGTCTTCAGCGTCATGCTCGCTACGTCAGACGATTGGCCT
A2-6nt-SEs_3	CATGACGCTGAAGCGAACGGAAGCACGGTTGCCTGTAGCGAG
A2-6nt-SEs_5	CAGGAGGTTGAGGCAGAGGCAACCGTGCTTCCGTTCGAGTCGGC
A2-6nt-SEs_4	GTCTGGGCCGACTACCTTGTGAGGTA
A3-6nt-SEs_1	AAGGCTGTAGGTTCGTGCTCAGAGACCTGCGTCAGACTGTAGCG
A3-6nt-SEs_3	GAGCACGAACCTAGTGGTGATCGCTCGTCGCCAGAGGTCTCT
A3-6nt-SEs_5	ATCAAGTTTGCCTTTACTGGCGACGAGCGATCACCACACTCCAA
A3-6nt-SEs_4	GTCTGGTTGGAGTCAGCCTTGAGGTA
A4-6nt-SEs_1	CAGCATGAGGTCACGTCTACGTCCACTCAGACAAAAGGGCGACA
A4-6nt-SEs_3	GTAGACGTGACCTGGTTCGATGCGAGACTTCGTGGAGTGGAC
A4-6nt-SEs_5	GGTTTACCAGCGCCAACACGAAGTCTCGCATCGAACCGCTATCCG

A4-6nt-SEs_4	GTCTGGGGATAGCCATGCTGGAGGTA
A5-6nt-SEs_1	CGTGCCTGCCATAGGACACCACCATGAGGCAGATAGCCGAACAA
A5-6nt-SEs_3	GGTGTCCTATGGCGATGGAAGTCGAGGTGACTCGCTCATGGT
A5-6nt-SEs_5	TTTTTAAGAAAAGTAACGAGTCACCTCGACTTCCATCACGCCTT
A5-6nt-SEs_4	GTCTGG AAGGCGTAGGCACGGAGGTA
A6-6nt-SEs_1	AGCATCCTCACACTGCGAGTAGCAAGGCAACGTCAAAAATGAAA
A6-6nt-SEs_3	ACTCGCAGTGTGACCGTACCTCTGTCCTAGTCGTGCCTTGCT
A6-6nt-SEs_5	AAACGATT TTTTGTTTACGACTAGGACAGAGGTACGGATCAGCC
A6-6nt-SEs_4	GTCTGGGGCTGATGGATGCTGAGGTA
**6nt Seed B adapter sequences**	
B1-6nt-SEs_1	AGGGATAGCAAGCCCACAACGTGAGGACACTTGGAGGCTGCACTC
B1-6nt-SEs_3	TGTCCTCACGTTGCTGGATGCCGATCCTACGACACCTCCAAG
B1-6nt-SEs_5	CGCTGACTTGTCGTAGGATCGGCATCCAGATAGGAACCCATGTAC
B1-6nt-SEs_2	CCAGAC GAGTGCAGAGTCAGCG TACCTC
B2-6nt-SEs_1	GAATTGCGAATAATAAGTGACCTTGCTGTACCGTCGAGATGGAGT
B2-6nt-SEs_3	ACAGCAAGGTCACCGCAGTTGGCACTAGGCGACATCGACGGT
B2-6nt-SEs_5	CCACAACCTGTCGCCTAGTGCCAACTGCGTTTTTTCACGTTGAAA
B2-6nt-SEs_2	CCAGAC ACTCCATCGGTTGTGG TACCTC
B3-6nt-SEs_1	ACCCTCAGCAGCGAAACGAGTACGGCAACACGGTGAGAGCCTACG
B3-6nt-SEs_3	GTTGCCGTACTCGACTGGTCACGAACGTCTCCAACTCACCGT
B3-6nt-SEs_5	GCTCTGCCTTGGAGACGTTCGTGACCAGTGACAGCATCGGAACGA
B3-6nt-SEs_2	CCAGAC CGTAGGCTGGCAGAGC TACCTC
B4-6nt-SEs_1	TGTATCATCGCCTGATCAACGGTACGAGATGCGAAGCACAGAGTG
B4-6nt-SEs_3	TCTCGTACCGTTGCCAGTAGACCTAGCCGACGTGGCTTCGCA
B4-6nt-SEs_5	GTCACGCTCACGTCGGCTAGGTCTACTGGAAATTGTGTCGAAATC
B4-6nt-SEs_2	CCAGAC CACTCTGTAGCGTGAC TACCTC
B5-6nt-SEs_1	CATTCAGTGAATAAGGACGCTATGCCTATCGCTCTAGGACCTCTG
B5-6nt-SEs_3	ATAGGCATAGCGTTGCTCCAGTCTGCTGCTCAGGCTAGAGCG
B5-6nt-SEs_5	CCACGACTCCTGAGCAGCAGACTGGAGCACTTGCCCTGACGAGAA
B5-6nt-SEs_2	CCAGAC CAGAGGTCAGTCGTGG TACCTC
B6-6nt-SEs_1	GAATACCACATTCAACACCGATGAGGATCACGGCACTCGACACTG
B6-6nt-SEs_3	GATCCTCATCGGTCAAGCGAAGGTGCGAGCCTGTAGTGCCGT
B6-6nt-SEs_5	GCGGACTGACAGGCTCGCACCTTCGCTTGTAATGCAGATACATAA
B6-6nt-SEs_2	CCAGAC CAGTGTCGCAGTCCGC TACCTC

The design of seeds 4nt A adapters and B adapters were described in Agrawal *et al*[3] and are also shown here.

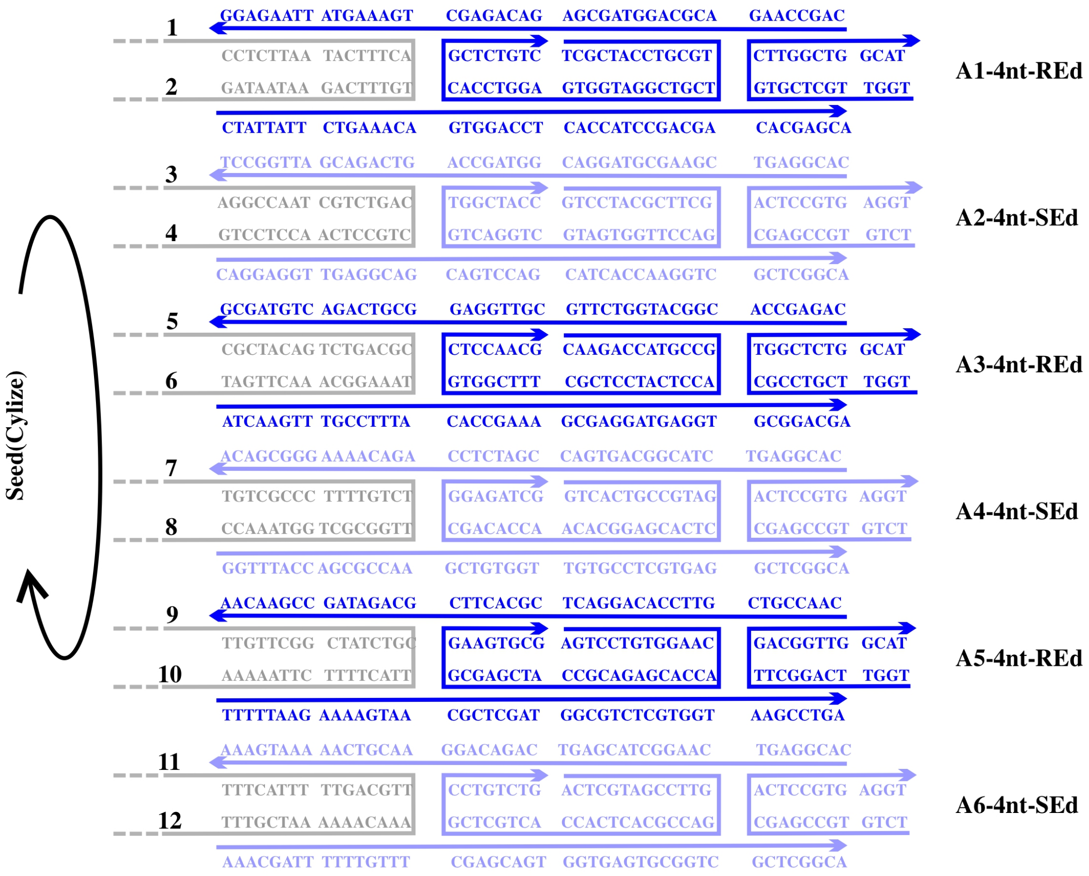

Supplementary Figure S45: Structures of the assembled seed A adapters for 4 nt nanotubes. The gray lines and corresponding sequences are parts of the M13mp18 scaffold.

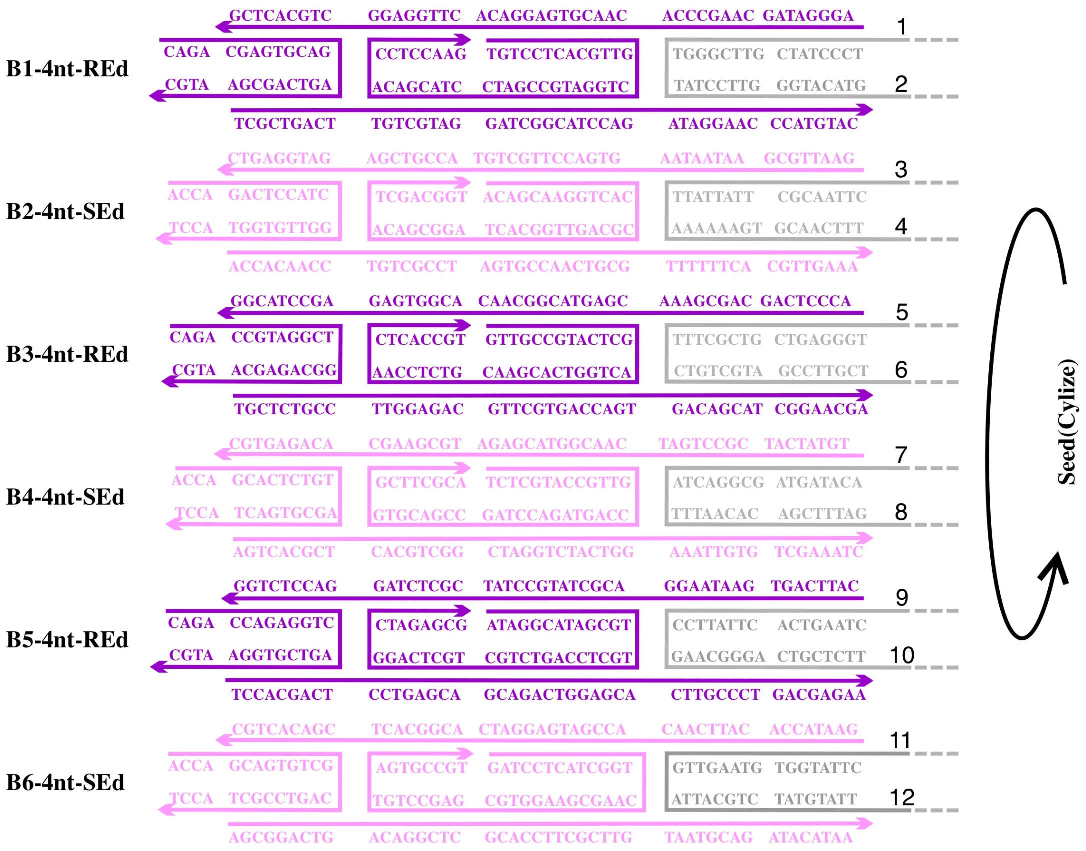

Supplementary Figure S46: Structures of the assembled seed B adapters for 4 nt nanotubes. The gray lines and corresponding sequences are parts of the M13mp18 scaffold.

Supplementary Table S33: Sequences of 4nt seed A adapters and 4nt seed B adapters.

4nt Seed A adapter sequences	
Strand name	Sequence
A-4bp-1REd_1	CAGCCAAGACGCAGGTAGCGAGACAGAGCTGAAAGTATTAAGAGG
A-4bp-1_2REd_3	TCGCT ACCTGCGTTCGTCGGA TGGTGAGGTCCACGCTCTGTC
A-4bp-1_2REd_5	CTATTATTCTGAAACAGTGGACCTCACCATCCGACGACACGAGCA
A-4bp-2REd_2	TGGTTGCTCGTGCTTGGCTGGCAT
A-4bp-3SEd_1	CACGGAGTCGAAGCGTAGGACGGTAGCCAGTCAGACGATTGGCCT GTCCT
A-4bp-3_4SEd_3	ACGCTTCGGACCTTGGTGA TGCTGGACTGTGGCT ACC
A-4bp-4SEd_5	CAGGAGGTTGAGGCAGCAGTCCAGCATCACCAAGGTCGCTCGGCA
A-4bp-3_4SEd_2	TCTGTGCCGAGCACTCCGTGAGGT
A-4bp-5REd_1	CAGAGCCACGGCA TGGTCTTGCGTTGGAGGCGTCAGACTGT AGCG
A-4bp-5_6REd_3	CAAGACCA TGCCGACCTCA TCCTCGCTTTCGGTGCTCCAACG
A-4bp-6REd_5	ATCAAGTTTGCCTTTACACCGAAAGCGAGGATGAGGTGCGGACGA
A-4bp-5_6REd_2	TGGTTCGTCCGCTGGCTCTGGCAT
A-4bp-7SEd_1	CACGGAGTCTACGGCAGTGACCGATCTCCAGACAAAAGGGCGACA
A-4bp-7_8SEd_3	GTCACTGCCGTAGCTCACGAGGCACAACCACAGCGGAGATCG
A-4bp-8SEd_5	GGTTTACCAGCGCCAAGCTGTGGTTGTGCCTCGTGAGGCTCGGCA

A-4bp-7_8SEd_2	TCTGTGCCGAGCACTCCGTGAGGT
A-4bp-9REd_1	CAACCGTCGTTCCACAGGACTCGCACTTCGCAGATAGCCGAACAA
A-4bp-9_10REd_3	AGTCCTGTGGAACACCACGAGACGCCA TCGAGCGGAAGTGCG
A-4bp-10SRd_5	TTTTTAAGAAAAGTAACGCTCGATGGCGTCTCGTGGTAAGCCTGA
A-4bp-9_10SRd_2	TGGTTCAGGCTTGACGGTTGGCAT
A-4bp-11SEd_1	CACGGAGTCAAGGCTACGAGTCAGACAGGAACGTCAAAAATGAAA
A-4bp-11_12SEd_3	ACTCGT AGCCTTGGACCGCACTCACCACTGCTCGCCTGTCTG
A-4bp-12SEd_5	AAACGA TTTTTTGTTTCGAGCAGTGGTGAGTGCGGTCGCTCGGCA
A-4bp-11_12SEd_2	TCTGTGCCGAGCACTCCGTGAGGT
**4nt Seed B adapter sequences**	
B-4bp-1REd_1	AGGGATAGCAAGCCCACAACGTGAGGACACTTGGAGGCTGCACTCG
B-4bp-1_2REd_3	TGTCCTCACGTTGCTGGATGCCGATCCTACGACACCTCCAAG
B-4bp-2REd_5	TCGCTGACTTGTCGTAGGATCGGCATCCAGATAGGAACCCATGTAC
B-4bp-1_2REd_4	CAGACGAGTGCAGAGTCAGCGAA TGC
B-4bp-3SEd_1	GAATTGCGAATAATAAGTGACCTTGCTGTACCGTCGAGATGGAGTC
B-4bp-3_4SEd_3	ACAGCAAGGTCACCGCAGTTGGCACTAGGCGACATCGACGGT
B-4bp-4SEd_5	ACCACAACCTGTCGCCTAGTGCCAACTGCGTTTTTTCACGTTGAAA
B-4bp-3_4SEd_4	ACCAGACTCCA TCGGTTGTGGT ACCT
B-4bp-5REd_1	ACCCTCAGCAGCGAAACGAGTACGGCAACACGGTGAGAGCCTACGG
B-4bp-5_6REd_3	GTTGCCGTACTCGACTGGTCACGAACGTCTCCAACTCACCGT
B-4bp-6REd_5	TGCTCTGCCTTGGAGACGTTCGTGACCAGTGACAGCA TCGGAACGA
B-4bp-5_6REd_4	CAGACCGTAGGCTGGCAGAGCAATGC
B-4bp-7SEd_1	TGTATCATCGCCTGATCAACGGTACGAGATGCGAAGCACAGAGTGC
B-4bp-7_8SEd_3	TCTCGT ACCGTTGCCAGT AGACCT AGCCGACGTGGCTTCGCA
B-4bp-8SEd_5	AGTCACGCTCACGTCGGCTAGGTCTACTGGAAATTGTGTCGAAATC
B-4bp-7_8SEd_4	ACCAGCACTCTGT AGCGTGACT ACCT
B-4bp-9REd_1	CATTCAGTGAATAAGGACGCTATGCCTATCGCTCTAGGACCTCTGG
B-4bp-9_10REd_3	ATAGGCATAGCGTTGCTCCAGTCTGCTGCTCAGGCT AGAGCG
B-4bp-10REd_5	TCCACGACTCCTGAGCAGCAGACTGGAGCACTTGCCCTGACGAGAA
B-4bp-9_10REd_4	CAGACCAGAGGTCAGTCGTGGAA TGC
B-4bp-11SEd_1	GAATACCACATTCAACACCGATGAGGATCACGGCACTCGACACTGC
B-4bp-11_12SEd_3	GATCCTCATCGGTCAAGCGAAGGTGCGAGCCTGTAGTGCCGT
B-4bp-12SEd_5	AGCGGACTGACAGGCTCGCACCTTCGCTTGTAATGCAGATACATAA
B-4bp-11_12SEd_4	ACCAGCAGTGTCGCAGTCCGCT ACCT

50.4 Biotin attachment strand design for AMDA

Seeded nanotubes were attached to cells *via* the hybridization of biotin attachment linker strands presenting BDC' tags, BDC' strands on seeds to BDC strands presenting the complementary BDC tag on cells. Six BDC' strands were attached to seeds on their right side, and in most experiments, 30 BDC' strands were also attached to the center of the unused M13mp18 scaffold (Supplementary Figure S47).

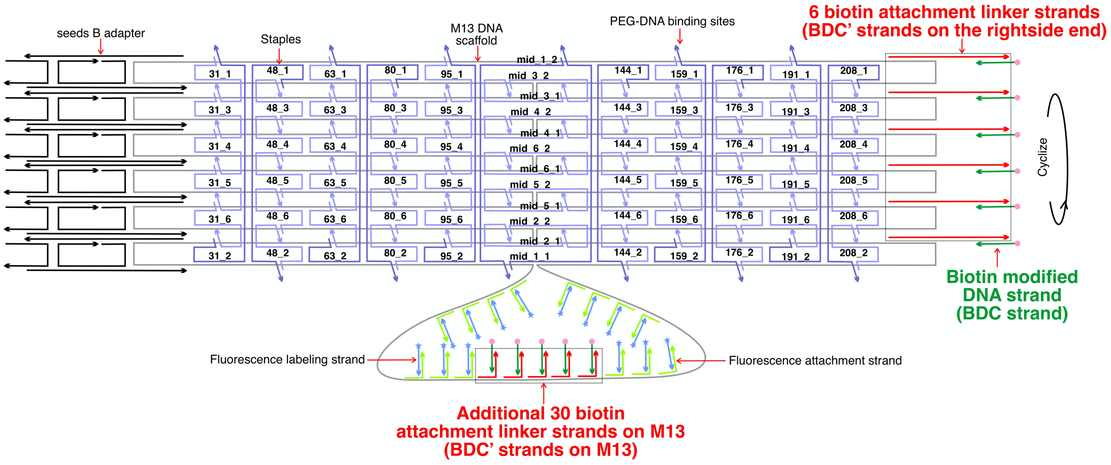

Supplementary Figure S47: Schematic showing the positions of the BDC strand attachment sites on the PEG coated seeds. There are six binding sites for BDC attachment strands on the right barrel end of the seeds (opposite the end where nanotube growth occurs), and 30 binding sites for 30 BDC attachment strands can be attached at the center of the region of the M13 scaffold not used to form the seed structure.

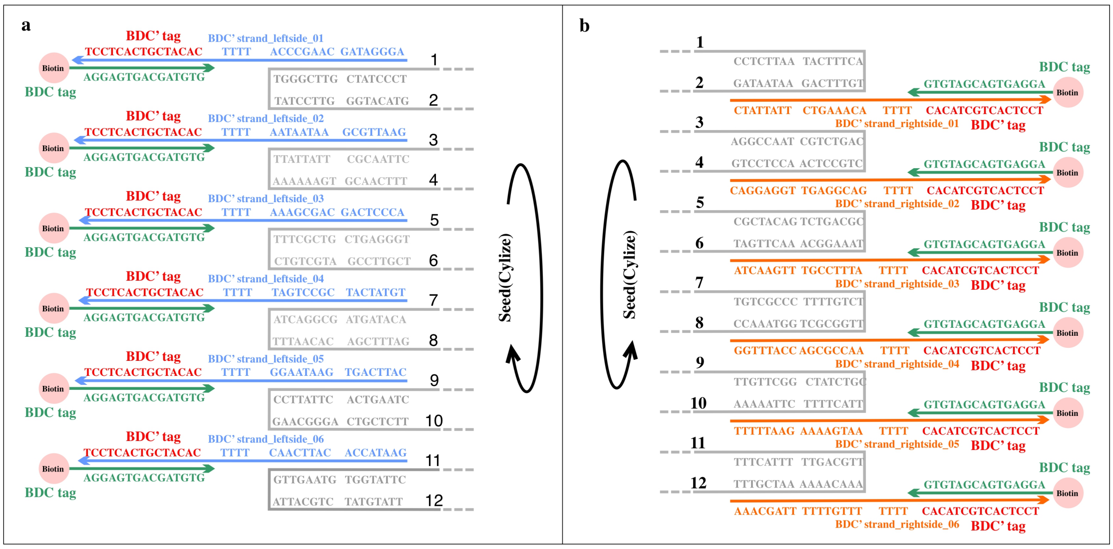

Supplementary Figure S48: Structure of the BDC tag attachment sites at the left ends of nanotube seeds A (a) and right ends of nanotube seed B (b). The gray lines and corresponding sequences are parts of the M13mp18 scaffold. These attachment strands have one domain complementary to BDC tag and another domain complementary to the M13 scaffold at the left or right end of seeds.

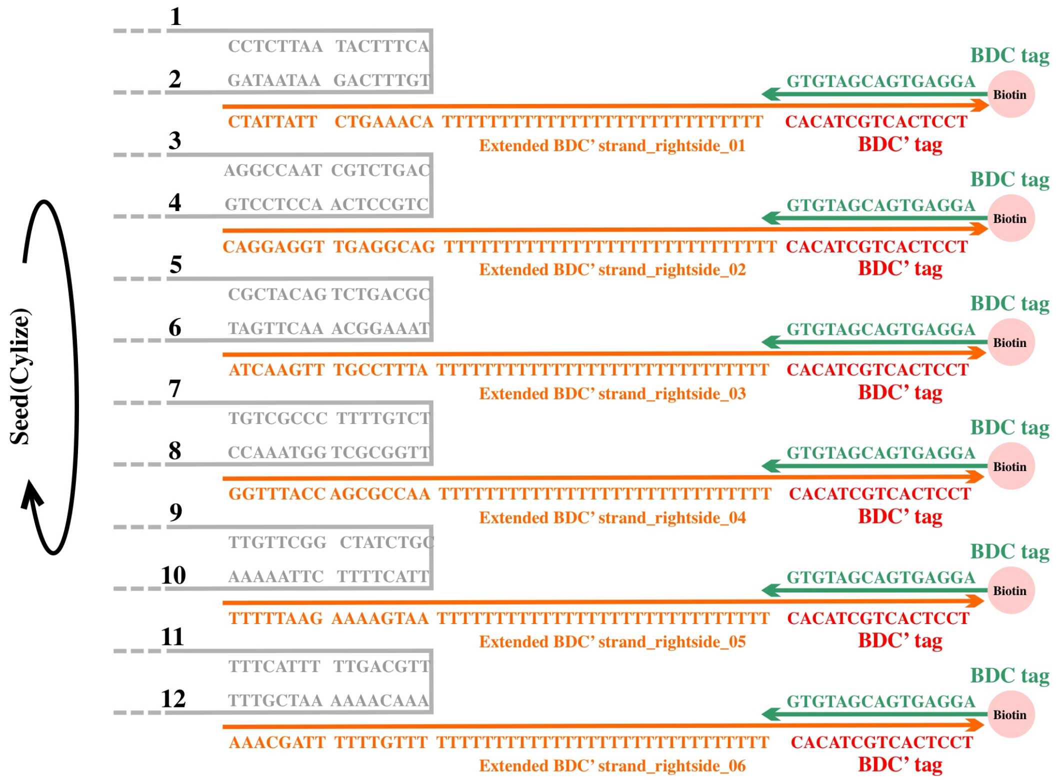

Supplementary Figure S49: Structure of the extended BDC tag attachment sites at the right ends of nanotube seeds. The gray lines and corresponding sequences are parts of the M13mp18 scaffold.

Supplementary Table S34 : Sequences of the biotin attachment strands which included the biotin left-side attachment strands for A seeds, the biotin right-side attachment strands for B seeds, the extended biotin attachment strand sequences for B seeds and the additional 30 biotin attachment strands on extra M13.

Strand name	Sequence
**Biotin left-side attachment strand sequences for A seeds**	
BDC' strand_leftside_01	AGGGATAGCAAGCCCATTTTCACATCGTCACTCCT
BDC' strand_leftside_02	GAATTGCGAATAATAATTTTCACATCGTCACTCCT
BDC' strand_leftside_03	ACCCTCAGCAGCGAAATTTTCACATCGTCACTCCT
BDC' strand_leftside_04	TGTATCATCGCCTGATTTTTCACATCGTCACTCCT
BDC' strand_leftside_05	CATTCAGTGAATAAGGTTTTCACATCGTCACTCCT
BDC' strand_leftside_06	GAATACCACATTCAACTTTTCACATCGTCACTCCT
**Biotin right-side attachment strand sequences for B seeds**	
BDC' strand_rightside_01	CTATTATTCTGAAACATTTTCACATCGTCACTCCT
BDC' strand_rightside_02	CAGGAGGTTGAGGCAGTTTTCACATCGTCACTCCT
BDC' strand_rightside_03	ATCAAGTTTGCCTTTATTTTCACATCGTCACTCCT
BDC' strand_rightside_04	GGTTTACCAGCGCCAATTTTCACATCGTCACTCCT
BDC' strand_rightside_05	TTTTTAAGAAAAGTAATTTTCACATCGTCACTCCT
BDC' strand_rightside_06	AAACGATTTTTTGTTTTTTTCACATCGTCACTCCT

Extended biotin attachment strand sequences for B seeds	
Extended BDC' strand_rightside_01	CTATTATTCTGAAACATTTTTTTTTTTTTTTTTTTTTTTTTTTTCACATCGTCACTCCT
Extended BDC' strand_rightside_02	CAGGAGGTTGAGGCAGTTTTTTTTTTTTTTTTTTTTTTTTTTTTCACATCGTCACTCCT
Extended BDC' strand_rightside_03	ATCAAGTTTGCCTTTATTTTTTTTTTTTTTTTTTTTTTTTTTTTCACATCGTCACTCCT
Extended BDC' strand_rightside_04	GGTTTACCAGCGCCAATTTTTTTTTTTTTTTTTTTTTTTTTTTTCACATCGTCACTCCT
Extended BDC' strand_rightside_05	TTTTTAAGAAAAGTAATTTTTTTTTTTTTTTTTTTTTTTTTTTTCACATCGTCACTCCT
Extended BDC' strand_rightside_06	AAACGATTTTTTGTTTTTTTTTTTTTTTTTTTTTTTTTTTTTTTCACATCGTCACTCCT

Sequences of the additional 30 biotin attachment strands on extra M13	
BDC' strand on unused_m13mp18_36	CAACTAATAGATTAGAGCCGTCAATTTTTCACATCGTCACTCCT
BDC' strand on unused_m13mp18_37	TATCTAAAATATCTTTAGGAGCACTTTTTCACATCGTCACTCCT
BDC' strand on unused_m13mp18_38	ACTGATAGCCCTAAAACATCGCCATTTTTCACATCGTCACTCCT
BDC' strand on unused_m13mp18_39	GAATGGCTATTAGTCTTTAATGCGCTTTTCACATCGTCACTCCT
BDC' strand on unused_m13mp18_40	AGAATACGTGGCACAGACAATATTTTTTCACATCGTCACTCCT
BDC' strand on unused_m13mp18_41	ATAGAACCCTTCTGACCTGAAAGCGTTTTCACATCGTCACTCCT
BDC' strand on unused_m13mp18_42	ATAAAAGGGACATTCTGGCCAACAGTTTTCACATCGTCACTCCT
BDC' strand on unused_m13mp18_43	GCAGATTCACCAGTCACACGACCAGTTTTCACATCGTCACTCCT
BDC' strand on unused_m13mp18_44	ATCGTCTGAAATGGATTATTTACATTTTTCACATCGTCACTCCT
BDC' strand on unused_m13mp18_45	ATGGAAATACCTACATTTTGACGCTTTTTCACATCGTCACTCCT
BDC' strand on unused_m13mp18_46	CCAGCCATTGCAACAGGAAAAACGCTTTTCACATCGTCACTCCT
BDC' strand on unused_m13mp18_47	CTGGTAATATCCAGAACAATATTACTTTTCACATCGTCACTCCT
BDC' strand on unused_m13mp18_48	GTAGAAGAACTCAAACTATCGGCCTTTTTCACATCGTCACTCCT
BDC' strand on unused_m13mp18_49	TGATTAGTAATAACATCACTTGCCTTTTTCACATCGTCACTCCT
BDC' strand on unused_m13mp18_50	AAATTAACCGTTGTAGCAATACTTCTTTTCACATCGTCACTCCT
BDC' strand on unused_m13mp18_51	CCGAGTAAAAGAGTCTGTCCATCACTTTTCACATCGTCACTCCT
BDC' strand on unused_m13mp18_52	GAAGTGTTTTTATAATCAGTGAGGCTTTTCACATCGTCACTCCT
BDC' strand on unused_m13mp18_53	GACAGGAACGGTACGCCAGAATCCTTTTTCACATCGTCACTCCT
BDC' strand on unused_m13mp18_54	AACAGGAGGCCGATTAAAGGGATTTTTTTCACATCGTCACTCCT
BDC' strand on unused_m13mp18_55	TCCTCGTTAGAATCAGAGCGGGAGCTTTTCACATCGTCACTCCT
BDC' strand on unused_m13mp18_56	GCTTTGACGAGCACGTATAACGTGCTTTTCACATCGTCACTCCT
BDC' strand on unused_m13mp18_57	CGCCGCTACAGGGCGCGTACTATGGTTTTCACATCGTCACTCCT
BDC' strand on unused_m13mp18_58	TAACCACCACACCCGCCGCGCTTAATTTTCACATCGTCACTCCT
BDC' strand on unused_m13mp18_59	TGGCAAGTGTAGCGGTCACGCTGCGTTTTCACATCGTCACTCCT
BDC' strand on unused_m13mp18_60	AAGCGAAAGGAGCGGGCGCTAGGGCTTTTCACATCGTCACTCCT
BDC' strand on unused_m13mp18_61	CGAACGTGGCGAGAAAGGAAGGGAATTTTCACATCGTCACTCCT
BDC' strand on unused_m13mp18_62	GATTTAGAGCTTGACGGGGAAAGCCTTTTCACATCGTCACTCCT
BDC' strand on unused_m13mp18_63	TAAATCGGAACCCTAAAGGGAGCCCTTTTCACATCGTCACTCCT
BDC' strand on unused_m13mp18_64	TTTTGGGGTCGAGGTGCCGTAAAGCTTTTCACATCGTCACTCCT

BDC' strand on unused_m13mp18_65	TACGTGAACCATCACCCAAATCAAGTTTTCACATCGTCACTCCT
**Universal biotin attachment strand sequence**	
biotin modified DNA strand (BDC tag)	/5BiosG/AGGAGTGACGATGTG

50.5 Amino attachment linker strands for SpyTag

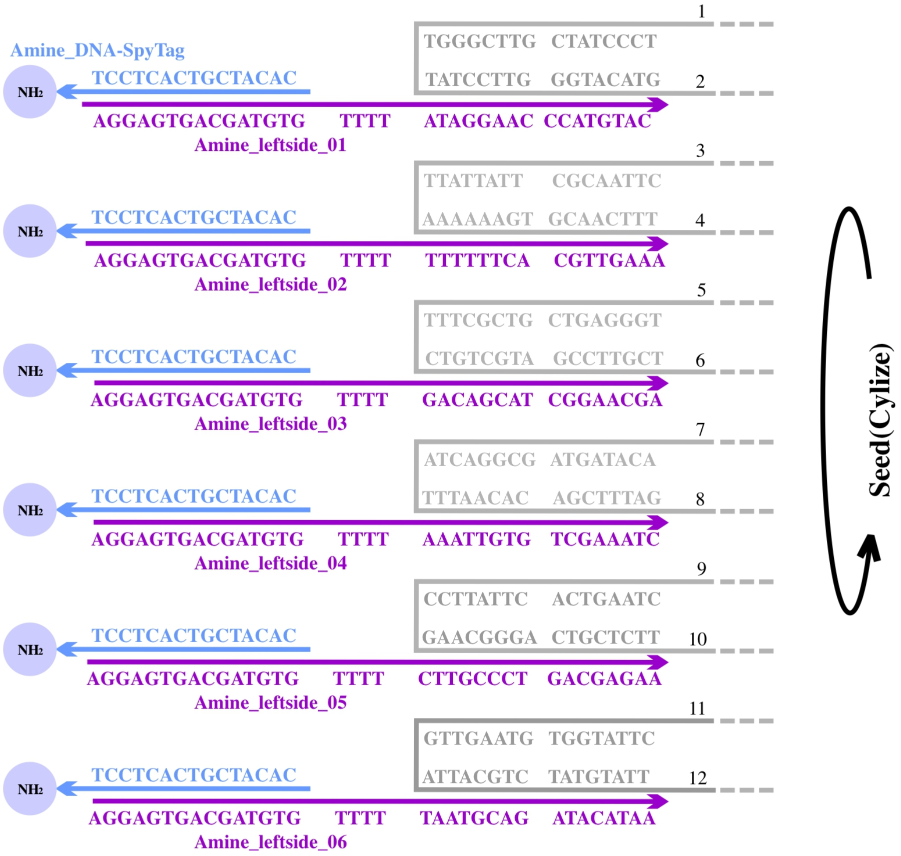

Supplementary Figure S50: Structure of the amino attachment sites at the left ends of nanotube seeds. The gray lines and corresponding sequences are parts of the M13mp18 scaffold. These attachment strands have one domain complementary to the SpyTag-DNA conjugate and another domain complementary to the M13 scaffold at the left end of seeds.

Supplementary Table S35 : Sequences of the amino attachment strands and amino modified DNA for conjugating with SpyTag

**Amino attachment strand sequences**	
Amine_leftside_01	AGGAGTGACGATGTGTTTTATAGGAACCCATGT AC
Amine_leftside_02	AGGAGTGACGATGTGTTTTTTTTTTCACGTTGA AA
Amine_leftside_03	AGGAGTGACGATGTGTTTTGACAGCATCGGAACGA
Amine_leftside_04	AGGAGTGACGATGTGTTTTAAATTGTGTCGAAATC
Amine_leftside_05	AGGAGTGACGATGTGTTTTCTTGCCCTGACGAGAA
Amine_leftside_06	AGGAGTGACGATGTGTTTTTAATGCAGATACATAA
**Amino-DNA sequence for conjugation with SpyTag**	
Amino_DNA_SpyTag	CACATCGTCACTCCT /3AmMO/

Supplementary Note S51: Sequence of the the plasmid DNA Integrin-SpyCatcher-GFP

AAGTACGCCCCCTATTGACGTCAATGACGGTAAATGGCCCGCCTGGCATTATGCCCAGTACA
TGACCTTATGGGACTTTCCTACTTGGCAGTACATCTACGTATTAGTCATCGCTATTACCATG
GTGATGCGGTTTTGGCAGTACATCAATGGGCGTGGATAGCGGTTTGACTCACGGGGATTTCC
AAGTCTCCACCCCATTGACGTCAATGGGAGTTTGTTTTGGCACCAAAATCAACGGGACTTTC
CAAAATGTCGTAACAACTCCGCCCCATTGACGCAAATGGGCGGTAGGCGTGTACGGTGGGAG
GTCTATATAAGCAGAGCTGGTTTAGTGAACCGTCAGATCCGCTAGCGCCACCATGAATTTAC
AACCAATTTTCTGGATTGGACTGATCAGTTCAGTTTGCTGTGTGTTTGCTGCGGCCGC**T**

ATGGTTGATACCTTATCAGGTTTATCAAGTGAGCAAGGTCAGTCCGGTGATATGACAATTGA
AGAAGATAGTGCTACCCATATTAAATTCTCAAAACGTGATGAGGACGGCAAAGAGTTAGCTG
GTGCAACTATGGAGTTGCGTGATTCATCTGGTAAAACTATTAGTACATGGATTTCAGATGGA
CAAGTGAAAGATTTCTACCTGTATCCAGGAAAATATACATTTGTCGAAACCGCAGCACCAGA
CGGTTATGAGGTAGCAACTGCTATTACCTTTACAGTTAATGAGCAAGGTCAGGTTACTGTAA
ATGGCAAAGCAACTAAAGGTGACGCTCATATTGAC

GCGGCCGCTAAGACTGTGATGCCTTACATTAGCACAACACCAGCTAAGCTCAGGAACCCTTG
CACAAGTGAACAGAACTGCACCAGCCCATTTGAGTGTCCCACTGGTCCAGACATCATTCCAA
TTGTAGCTGGTGTGGTTGCTGGAATTGTTCTTATTGGCCTTGCATTACTGCTGATATGGAAG
CTGTTAATGATAATTCATGACAGAAGGGAG

GTACCGGTCGCCACC

ATGGTGAGCAAGGGCGAGGAGCTGTTCACCGGGGTGGTGCCCATCCTGGTCGAGCTG
GACGGCGACGTAAACGGCCACAAGTTCAGCGTGTCCGGCGAGGGCGAGGGCGATGCC
ACCTACGGCAAGCTGACCCTGAAGTTCATCTGCACCACCGGCAAGCTGCCCGTGCCC
TGGCCCACCCTCGTGACCACCCTGACCTACGGCGTGCAGTGCTTCAGCCGCTACCCC
GACCACATGAAGCAGCACGACTTCTTCAAGTCCGCCATGCCCGAAGGCTACGTCCAG
GAGCGCACCATCTTCTTCAAGGACGACGGCAACTACAAGACCCGCGCCGAGGTGAAG
TTCGAGGGCGACACCCTGGTGAACCGCATCGAGCTGAAGGGCATCGACTTCAAGGAG
GACGGCAACATCCTGGGGCACAAGCTGGAGTACAACTACAACAGCCACAACGTCTAT
ATCATGGCCGACAAGCAGAAGAACGGCATCAAGGTGAACTTCAAGATCCGCCACAAC
ATCGAGGACGGCAGCGTGCAGCTCGCCGACCACTACCAGCAGAACACCCCCATCGGC
GACGGCCCCGTGCTGCTGCCCGACAACCACTACCTGAGCACCCAGTCCGCCCTGAGC
AAAGACCCCAACGAGAAGCGCGATCACATGGTCCTGCTGGAGTTCGTGACCGCCGCC
GGGATCACTCTCGGCATGGACGAGCTGTACAAG

References:

1 Mohammed, A. M., Šulc, P., Zenk, J. & Schulman, R. Self-assembling DNA nanotubes to connect molecular landmarks. *Nature Nanotechnology* **12**, 312, doi:10.1038/nnano.2016.277 https://www.nature.com/articles/nnano.2016.277#supplementary-information (2016).

2 Li, Y. & Schulman, R. DNA Nanostructures that Self-Heal in Serum. *Nano Letters* **19**, 3751-3760, doi:10.1021/acs.nanolett.9b00888 (2019).

3 Agrawal, D. K. *et al.* Terminating DNA Tile Assembly with Nanostructured Caps. *ACS Nano* **11**, 9770-9779, doi:10.1021/acsnano.7b02256 (2017).

4 Martin, T. G. & Dietz, H. Magnesium-free self-assembly of multi-layer DNA objects. *Nature Communications* **3**, 1103, doi:10.1038/ncomms2095 (2012).

5 Perrault, S. D. & Shih, W. M. Virus-Inspired Membrane Encapsulation of DNA Nanostructures To Achieve In Vivo Stability. *ACS Nano* **8**, 5132-5140, doi:10.1021/nn5011914 (2014).

6 Wilkinson, M. H. F. & Schut, F. *Digital Image Analysis of Microbes: Imaging, Morphometry, Fluorometry and Motility Techniques and Applications*. (Wiley, 1998).

7 Tinevez, J.-Y. *et al.* TrackMate: An open and extensible platform for single-particle tracking. *Methods* **115**, 80-90, doi:https://doi.org/10.1016/j.ymeth.2016.09.016 (2017).

8 Mohammed, A. M. & Schulman, R. Directing Self-Assembly of DNA Nanotubes Using Programmable Seeds. *Nano Letters* **13**, 4006-4013, doi:10.1021/nl400881w (2013).

9 Schaffter, S. W., Scalise, D., Murphy, T. M., Patel, A. & Schulman, R. Feedback regulation of crystal growth by buffering monomer concentration. *Nature Communications* **11**, 6057, doi:10.1038/s41467-020-19882-8 (2020).

10 Stephanopoulos, N. *et al.* Bioactive DNA-Peptide Nanotubes Enhance the Differentiation of Neural Stem Cells Into Neurons. *Nano Letters* **15**, 603-609, doi:10.1021/nl504079q (2015).

11 Zakeri, B. *et al.* Peptide tag forming a rapid covalent bond to a protein, through engineering a bacterial adhesin. *Proceedings of the National Academy of Sciences* **109**, E690-E697, doi:10.1073/pnas.1115485109 (2012).

12 Benedetto, S. *et al.* Quantification of the expression level of integrin receptor αvβ3 in cell lines and MR imaging with antibody-coated iron oxide particles. *Magnetic Resonance in Medicine* **56**, 711-716, doi:10.1002/mrm.21023 (2006).

13 Schaffter, S. W. *et al.* Reconfiguring DNA Nanotube Architectures via Selective Regulation of Terminating Structures. *ACS Nano* **14**, 13451-13462, doi:10.1021/acsnano.0c05340 (2020).

14 Dirks, R. M., Bois, J. S., Schaeffer, J. M., Winfree, E. & Pierce, N. A.

Thermodynamic Analysis of Interacting Nucleic Acid Strands. *SIAM Review* **49**, 65-88, doi:10.1137/060651100 (2007).

15 Zadeh, J. N. *et al.* NUPACK: Analysis and design of nucleic acid systems. *Journal of Computational Chemistry* **32**, 170-173, doi:10.1002/jcc.21596 (2011).

16 van der Walt, S. *et al.* scikit-image: image processing in Python. *PeerJ* **2**, e453, doi:10.7717/peerj.453 (2014).

17 Canny, J. A Computational Approach to Edge Detection. *IEEE Transactions on Pattern Analysis and Machine Intelligence* **PAMI-8**, 679-698, doi:10.1109/TPAMI.1986.4767851 (1986).

18 Proudman, I. & Pearson, J. R. A. Expansions at small Reynolds numbers for the flow past a sphere and a circular cylinder. *Journal of Fluid Mechanics* **2**, 237-262, doi:10.1017/S0022112057000105 (1957).

19 Lagrée, P.-Y. Small Re flows, ε= Re≪1. *Paris: CNRS and UPMC university* (2013).

20 https://ibidi.com/img/cms/support/AN/AN11_Shear_stress.pdf.

21 Rothemund, P. W. K. *et al.* Design and Characterization of Programmable DNA Nanotubes. *Journal of the American Chemical Society* **126**, 16344-16352, doi:10.1021/ja044319l (2004).
