# Supplementary material for: Growth and site-specific organization of micron-scale biomolecular devices on living mammalian cells
Source: Nat Commun. 2021 Sep 30;12:5729. doi: 10.1038/s41467-021-25890-z (PMC8484582; doi:10.1038/s41467-021-25890-z)
Supplement: Supplementary file 2 — Reporting Summary [file 41467_2021_25890_MOESM2_ESM.pdf]

nature research

Corresponding author(s): Rebecca Schulman

Last updated by author(s): 08/13/2021

Reporting Summary

Nature Research wishes to improve the reproducibility of the work that we publish. This form provides structure for consistency and transparency in reporting. For further information on Nature Research policies, see Authors & Referees and the Editorial Policy Checklist .

Statistics

For all statistical analyses, confirm that the following items are present in the figure legend, table legend, main text, or Methods section.

n/a	Confirmed	
☐	☒	The exact sample size (*n*) for each experimental group/condition, given as a discrete number and unit of measurement
☐	☒	A statement on whether measurements were taken from distinct samples or whether the same sample was measured repeatedly
☒	☐	The statistical test(s) used AND whether they are one- or two-sided<br>*Only common tests should be described solely by name; describe more complex techniques in the Methods section.*
☒	☐	A description of all covariates tested
☐	☒	A description of any assumptions or corrections, such as tests of normality and adjustment for multiple comparisons
☐	☒	A full description of the statistical parameters including central tendency (e.g. means) or other basic estimates (e.g. regression coefficient) AND variation (e.g. standard deviation) or associated estimates of uncertainty (e.g. confidence intervals)
☒	☐	For null hypothesis testing, the test statistic (e.g. *F*, *t*, *r*) with confidence intervals, effect sizes, degrees of freedom and *P* value noted<br>*Give P values as exact values whenever suitable.*
☒	☐	For Bayesian analysis, information on the choice of priors and Markov chain Monte Carlo settings
☒	☐	For hierarchical and complex designs, identification of the appropriate level for tests and full reporting of outcomes
☒	☐	Estimates of effect sizes (e.g. Cohen's *d*, Pearson's *r*), indicating how they were calculated

Our web collection on statistics for biologists contains articles on many of the points above.

Software and code

Policy information about availability of computer code

Data collection	Andor SOLOS Version 4.31.30024.0 (Oxford Instruments), NIS-Elements AR 5.02.01 (Nikon), ZEN 2 (blue edition), DB FACSDiva Softwarev8.0
Data analysis	ImageJ Fiji (version 2.0.0-rc-59/1.51n), Microsoft Excel for Mac (version 16.37 (20051002)), Matlab R2017b (9.3.0.713579) and Matlab R2019a, Python (2.7.16), scikit-image (0.14.2), scipy (1.2.1), matplotlib (2.2.3), numpy (1.16.5), Canny algorithm 0.19.0.dev0 (sci-kit image), ZEN2.3 SP1 software (Zeiss), NIS Elements Viewer 4.11.0, FLOWJO 10.4, TrackMate7 software (v3.4.2) incorporated into ImageJ Fiji.

For manuscripts utilizing custom algorithms or software that are central to the research but not yet described in published literature, software must be made available to editors/reviewers. We strongly encourage code deposition in a community repository (e.g. GitHub). See the Nature Research guidelines for submitting code & software for further information.

Data

Policy information about availability of data

All manuscripts must include a data availability statement. This statement should provide the following information, where applicable:

- Accession codes, unique identifiers, or web links for publicly available datasets
- A list of figures that have associated raw data
- A description of any restrictions on data availability

The raw data and code that support the findings of this study are available can be accessed (https://doi.org/10.7281/T1/CUEL0W). Source data are provided with this paper.

Field-specific reporting

Please select the one below that is the best fit for your research. If you are not sure, read the appropriate sections before making your selection.

- [x] Life sciences
- [ ] Behavioural & social sciences
- [ ] Ecological, evolutionary & environmental sciences

For a reference copy of the document with all sections, see nature.com/documents/nr-reporting-summary-flat.pdf

Life sciences study design

All studies must disclose on these points even when the disclosure is negative.

Sample size

No sample size calculation was performed. All experiments were performed using sample size based on standard protocols in the field.

Data exclusions

The experiments for which data were excluded ans the reason for the exclusion were described in detail in the Supplementary Note S6, S7.3, S19, S22, S24, S27, S29, S33, S41, S42, S45, S46. All data exclusion criteria were pre-established.

Replication

All experiments were performed in triplicate cell cultures on different days at minimum. We specified the number of biological replicates in the respective figure legends. All replication attempts were successful.

Randomization

We plated the cell in random positions in multi-well plates and randomly assigned them to different experiment groups. We randomly took fluorescence images under microscope.

Blinding

No blinding was conducted due to the nature of the experiments. The data collection and analysis were not necessary be blinded because most of our experiments here, including the nanotube attachment and growth on cell membrane and the nanotube bending under fluid flow, can be distinguished with the control experiments just by looking at them under the confocal microscope.

Reporting for specific materials, systems and methods

We require information from authors about some types of materials, experimental systems and methods used in many studies. Here, indicate whether each material, system or method listed is relevant to your study. If you are not sure if a list item applies to your research, read the appropriate section before selecting a response.

Materials & experimental systems

n/a	Involved in the study	
	x	Antibodies
	x	Eukaryotic cell lines
x		Palaeontology
x		Animals and other organisms
x		Human research participants
x		Clinical data

Methods

n/a	Involved in the study	
x		ChIP-seq
	x	Flow cytometry
x		MRI-based neuroimaging

Antibodies

Antibodies used

EGFR monoclonal antibody (H11) from Thermo Fisher, product MA5-13070, used at 1:100 dilution. The Alexa fluor 647-conjugated secondary antibody from Thermo Fisher, product A-21236, used at 1:100 dilution. The biotin-conjugated secondary antibody from Thermo Fisher, product 31800, used in 1:500 dilution. Integrin β1 Antibody (K-20) from Santa Cruz Biotechnology, product sc-18887, used in 1:50 dilution.

Validation

The antibodies used in this work are all commercial and have been validated. Detailed information can be found on the website from the manufacturer or related publications listed below:
EGFR monoclonal antibody (H11): https://www.thermofisher.com/antibody/product/EGFR-Antibody-clone-H11-Monoclonal/MA5-13070
Alexa fluor 647-conjugated secondary antibody:https://www.thermofisher.com/antibody/product/Goat-anti-Mouse-IgG-H-L-Highly-Cross-Adsorbed-Secondary-Antibody-Polyclonal/A-21236
Biotin-conjugated secondary antibody: https://www.thermofisher.com/antibody/product/Goat-anti-Mouse-IgG-H-L-Secondary-Antibody-Polyclonal/31800
Integrin β1 Antibody (K-20): https://www.scbt.com/p/integrin-beta1-antibody-k-20

Eukaryotic cell lines

Policy information about cell lines

Cell line source(s)	HeLa and HEK293 from ATCC.
Authentication	No further authentication was done for cell lines.
Mycoplasma contamination	Beyond initial testing for mycoplasma contamination which were negative, cell lines were not tested for the rest of the experiments and data collection.
Commonly misidentified lines (See ICLAC register)	No commonly misidentified lines.

Flow Cytometry

Plots

Confirm that:

- [x] The axis labels state the marker and fluorochrome used (e.g. CD4-FITC).
- [x] The axis scales are clearly visible. Include numbers along axes only for bottom left plot of group (a 'group' is an analysis of identical markers).
- [x] All plots are contour plots with outliers or pseudocolor plots.
- [x] A numerical value for number of cells or percentage (with statistics) is provided.

Methodology

Sample preparation	HEK293 Cells were grown in tissue culture. The cells were trypsinized and ATTO488 labeled DNA nanotube seeds were attached to these suspended cells through the EGFR antibody AMDA methods as described in the text.
Instrument	FACSCanto flow cytometer (BD Biosciences, USA)
Software	BD FLOWJO software was used for analysis
Cell population abundance	No post-sorting was performed due to the nature of the experiments.
Gating strategy	Negative control (blank) was used to establish gates for each sample by measure the FSC and SSC. Gates were drawn to collect cells according to the FSC and SSC. The same gate was applied to all the samples.

- [x] Tick this box to confirm that a figure exemplifying the gating strategy is provided in the Supplementary Information.
