# Supplementary material for: Growth and site-specific organization of micron-scale biomolecular devices on living mammalian cells
Source: Nat Commun. 2021 Sep 30;12:5729. doi: 10.1038/s41467-021-25890-z (PMC8484582; doi:10.1038/s41467-021-25890-z)
Supplement: Supplementary file 9 — Description of Additional Supplementary Files [file 41467_2021_25890_MOESM9_ESM.pdf]

Title: Supplementary Movie 1
Description: DNA nanotubes anchored to EGFR on HeLa cell membranes using AMDA. Experiments were performed as described in Supp. Note S26. Nanotubes were labeled with Cy3 (green), nanotube seeds with atto647 (red). Streptavidin-Alex488(blue)-conjugated EGFR antibodies are visible on the cell surface. The scale bar is 10µm. The movie was captured at 0.5Hz (2 seconds per frame) under a Zeiss AxioObserver Yokogawa CSU-X1 spinning disk confocal microscope with a 60x oil objective. A Gaussian blur filter (radius:0.5) in ImageJ was applied to all the images of the movie to reduce image background.

Title: Supplementary Movie 2:
Description: DNA nanotubes anchored to EGFR on the cell membrane of a suspended HEK293 cell via AMDA. Experiments were performed as described in Supp. Note S28. Nanotubes were labeled with Cy3 (green), nanotube seeds with atto647 (red), and treptavidin-Alex488(blue)-conjugated EGFR antibodies are visible on the cell surface. The scale bar is 5µm. The movie was generated using ZEN2.3 SP1 software (Zeiss) from a multicolor z-stack of confocal micrographs taken at 0.27 µm apart in height from cell bottom to cell top.

Title: Supplementary Movie 3:
Description: DNA nanotubes anchored to the tops of HeLa cell membrane via AMDA in the presence of fluid shear stresses of 0 dyn per cm^2 (a), 0.05 dyn per cm^2 (b), 0.2 dyn per cm^2 (c), and 1 dyn per cm^2 (d). Experiments were performed as described in Supp. Note S39 and S40. Nanotubes were labeled with Cy3 (green), nanotube seeds with atto647 (red), and streptavidin-Alex488(blue)-conjugated EGFR antibodies are present on the cell surface. The scale bar is 10µm. The movie was captured at 0.2Hz (5 seconds per frame). A Gaussian blur filter (radius:1.00) in ImageJ was applied to all the images to reduce image background.

Title: Supplementary Movie 4:
Description: End-to-end joined nanotubes anchored on HeLa cells under a gentle shear stress (0.32 dyn/cm^2). Experiments were performed as described in Supp. Note S46. Nanotubes were labeled with Cy3 (green), the “glue” monomers with atto647 (red). Seeds on the anchored and capped seeded nanotubes were not fluorescently labeled. The cells were visualized using bright field (gray) which is overlaid with the fluorescence signals. The scale bar is 10µm. The movie was generated by taking 20 continuous cycles in which fluorescence images in the Cy3, atto647 channels and a bright field image were captured without latency.

Title: Supplementary Movie 5:
Description: End-to-end joined nanotubes on the membrane of live HeLa cell membrane in 0.6% v/v methylcellulose (IMDM) media. Experiments were performed as described in Supp. Note S48. Nanotubes were labeled with Cy3 (green), anchored nanotube seeds and “glue” monomers with atto647 (red) and HeLa cells were transfected with GFP (blue). The nanotube caps were unlabeled. The scale bar is 10µm. The movie was generated using ZEN2.3 SP1 (Zeiss) from a multicolor z-stack of confocal micrographs taken at 0.27 µm apart in height from cell bottom to cell top.
